# Synthesis of Amino Core Compounds of Galactosyl Phytosyl Ceramide Analogs for Developing iNKT-Cell Inducers

**DOI:** 10.3390/molecules17033058

**Published:** 2012-03-12

**Authors:** Yin-Cheng Huang, Li-Wu Chiang, Kai-Shiang Chang, Wen-Chin Su, Yi-Hsian Lin, Kee-Ching Jeng, Kun-I Lin, Kuo-Yen Liao, Ho-Lein Huang, Chung-Shan Yu

**Affiliations:** 1 Department of Neurosurgery, Chang Gung Memorial Hospital and Department of Medicine, Chang Gung University, Taoyuan 33305, Taiwan; 2 Department of Biomedical Engineering and Environmental Sciences, National Tsing-Hua University, No. 101 sec.2, Guang-Fu Rd., Hsinchu 30043, Taiwan; 3 Department of Medical Research, Taichung Veterans General Hospital, Taichung 40705, Taiwan; 4 Department of Obstetrics and Gynecology, Chang Bing Show Chwan Memorial Hospital, Lukang Zhen, Changhua 50544, Taiwan; 5 Institute of Nuclear Engineering and Science, National Tsing-Hua University, Hsinchu 30043, Taiwan

**Keywords:** phytosphingosine, library, cancer, immune, glycosylation

## Abstract

1-Aminophytosphingosine and 6-aminogalactosyl phytosphingosine were prepared in 61% and 40% yield libraries with 44 carboxylic acids showed that a 4-butylbenzoic acid-derived product exe, respectively. Glycosylation using benzoyl-protected lipid resulted in better α-selectivity for ceramide analogs, but the yield was less than that obtained with benzyl moieties. Screening the amide rted less cytotoxicity. These analogs were purified for validation of immunological potencies and the α-GalCer analog but not the sphingosine analog stimulated human iNKT cell population.

## 1. Introduction

α-Galactosyl ceramide (α-GalCer) [1,2], also called KRN7000, has attracted great attention due to its antitumor effects [3,4,5]. The bioactivity was initiated through the initial binding of α-GalCer to CD1d receptor expressed on antigen presenting cells [6,7], followed by presenting to invariant natural killer T (iNKT) cells [8,9]. This signifies the release of several cytokines such as IFNγ and IL-4 which are categorized as belonging to the TH1 and TH2 pathways, respectively [10,11]. Whereas both types of cytokine could be elicited through α-GalCer, the recent focus has centered on the skewing effect of the TH1/TH2 ratio to direct toward a possible medical indication [12,13]. For example, preferential TH1 signaling is related to cancer therapy, whereas TH2 is associated with antimicrobial activity [14]. However, human clinical trials of α-GalCer [15] encountered reduced levels of iNKT cell populations similar to a recent animal study [16]. This might be partially due to the deglycosylated ceramide which mediated the subsequent apoptosis/necrosis cascade.

**Scheme 1:**
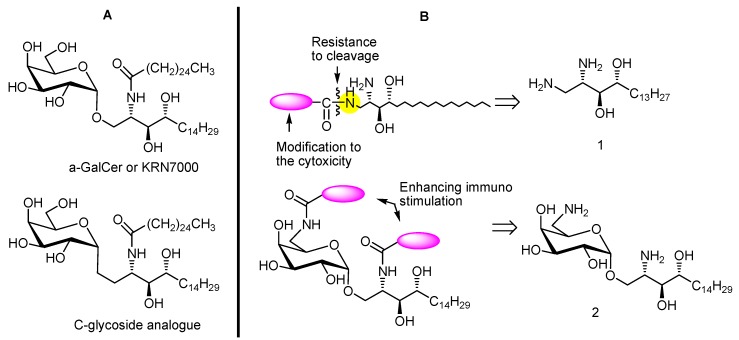
Panel (**A**) α-GalCer and structural analogue with stable glycosidic bond may resist metabolic cleavage. Panel (**B**) Structural modification using amide may resist metabolic cleavage. The library moieties to be prepared may modify the cytotoxicity as well as immunostimulating effect.

Numerous approaches to structural modification of the sugar head [7,12,17,18,19,20] and truncation of the sphingosine backbone [19,21] or acyl chain [22,23] as well as incorporation of unsaturation in the acyl chain [24] have generated some bioactive leads. For example, some of the truncated compounds are active in the TH2-biased pathway [19,21,25], whereas only rarer cases lead towards the TH1-biased pathway [24,26]. α-GalCer analogs with C-modified glycosidic linkages have been shown to possess this feature, probably due to their inertness to metabolic cleavage of the glycosidic bond [24]. Hence, an amide bond with reasonable inertness might provide an alternative to the glycosidic bond. Consequently an amide library derived from 1-amino phytosphingosine analogs **1** with variation of acyl groups was prepared and screened to find which structural features had moderate cytotoxicities. With such a structural type in hand, compounds that incorporated this acyl group into α-galactosyl sphingosine **2** at the sugar 6-amino and (or) the 2-amino group of the sphingoid base were evaluated for immunostimulating potency. The concept for the design of our synthesis and screening is outlined in Scheme 1B.

The structure activity relationship (SAR) of α-GalCer complexed with the CD1d receptor shows that the 6-OH group of the galactose portion is not required for hydrogen bonding [27,28], thus providing a possibility for structural modification [26,29,30,31]. Some variants are tolerated by TCR-glycolipid-CD1d interaction [31,32]. Various modifications at C-6 of the sugar portion using the amino group [26,29,33] in both synthetic and library fashion for SAR elaboration have been reported in the literature [12,20]. For diversifying the compound pools, a library approach could provide a straightforward manner. Recent development of α-GalCer libraries including the solution-phase-synthesis approach of Wong [12] and the solid phase synthesis approach of Howell [20] have generated a number of compounds. Both purity and identity can be achieved in this approach.

Recently, 6′-azidogalactosyl 2-aminosphingosine analogs and their relevant galactosyl ceramide analogs were prepared by using a delicate synthetic design [33]. By employing sophisticated glycosylation conditions [34,35,36], a reactive silyl protected 1-iodogalactoside as donor could be coupled with less reactive acceptors to provide α-GalCer in a good yield and in exclusive α-stereoselective fashion.

In addition, glycosylation using imidates [37], thiosugars [38], and fluorosugars [31] have been well-documented. These results indicate that the glycosylation is very sensitive and depends heavily on the matching reactivities between donors and acceptors [34]. Satisfactory yield and α-selectivity could be achieved through glycosylation of an armed donor and disarmed acceptor. The present work comprised three parts: (1) the preparation of a novel 1,2-diamino phytosphingosine; (2) preparation of 6-azido thiogalactoside with ester-type and ether-type donors for obtaining glycosylated compounds in both acceptable yield and stereoselectivity; and (3) the *in-situ* screening [39,40,41] of the cellular cytotoxicity and the validation of the purified compounds [42].

## 2. Results and Discussion

**Scheme 2:**
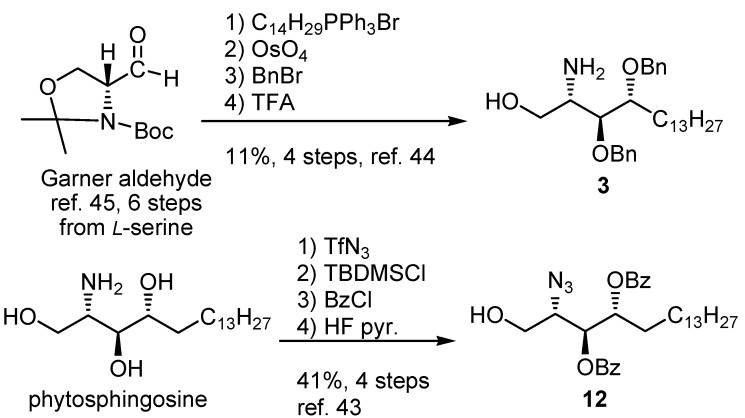
Preparation of the starting materials **3**, **12** for the present study.

Both commercially available [43] and well-protected phytosphingosine [44] obtained from the Garner aldehyde [45] were used as starting materials to prepare the target compounds **2** and **1** (Scheme 2). Thus, the current synthetic strategy attempted to use the azide group as a masked functionality for both the phytosphingosine base and sugar portion.

**Scheme 3:**
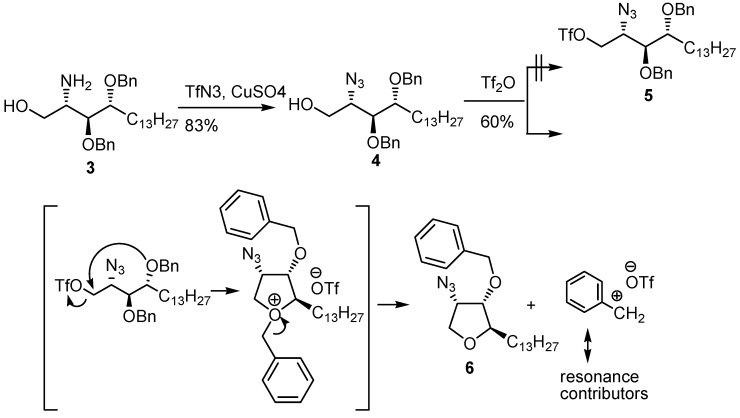
Unexpected ring closure during the preparation of triflate compound **5** and the probable mechanism that leads to its formation.

The azido-compound [46] was introduced under a mild reaction conditions using copper-catalysis (Scheme 3). The subsequent introduction of the triflate did not lead to the desired product **5** but only the cyclized analog of 2-*epi*-jaspin B (**6**), a reported recently potential anti-cancer compound [47]. Whereas the triflate is a very good leaving group with a potency of 100 times than that of tosylate [48], the leaving tendency was insufficient to induce the desired ring closure. A trace amount of acid generated during the chromatography might weaken the ether protecting group [49]. On the other hand, the intramolecular SN_2_ reaction mediated by a suitable stereochemistry has been addressed [50]. In the present case, the nucleophilicity of the OBn group might be displayed by orienting itself through a conformational change of the backbone as evidenced from the ^1^H-NMR in the preparations. Hence, the complex between the five-membered cation and the triflate was converted to the neutral 2-*epi* jaspin B **6** along with the benzyl cation stabilized by the resonance contributors (Scheme 3).

**Scheme 4:**
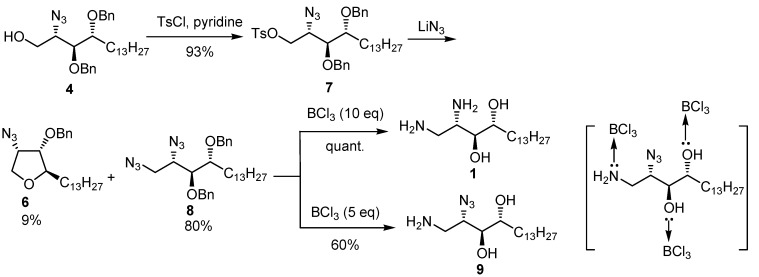
Preparation of the 1,2-diamino and 1-amino-2-azido phytosphingosine analogs **1** and **9**. Formation of the complex proposed to explain the partial reduction of the azido group when using 5 equivalents of BCl_3_.

Introduction of the tosyl group using tosyl chloride took place smoothly without encountering the problem of ring closure (Scheme 4). The subsequent nucleophilic attack by azide afforded the desired diazido compound **8** in 80% yield accompanied with the cyclized 2-*epi*-jaspin B analog **6**. The following reduction using BCl_3_ gave the desired diaminophytosphingosine analog **1** in quantitative yield. Interestingly, when using less equivalents of BCl_3_ (5 eq.), the primary azide was selectively reduced to afford the monoamino compound **9**. The probable cause for the partial reduction of the protecting groups is proposed to be deactivation of the remaining unreacted BCl_3_ to form a complex with the reduced amino group and to a slight extent with the oxo groups (Scheme 4) [51,52].

As observed in the ^1^H-NMR for the diamino compound **1**, the two broad peaks at 8.26 and 8.47 ppm indicated the presence of ammonium complexes. Although both ^1^H- and ^13^C-NMR spectra for the slightly-light-brown sample were satisfactory, the compound could be purified to a white solid by elution from an ion exchange (OH^−^) resin.

**Figure 1:**
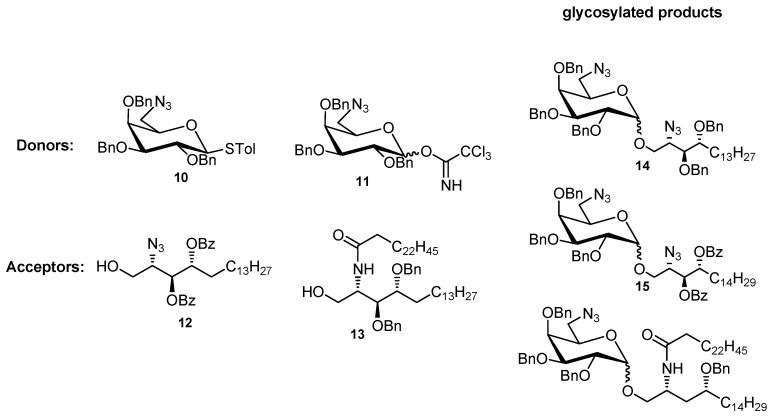
Donors and acceptors used for preparing glycosylated products.

For synthesizing the galactosyl phytosphingosine, the 6-azido galactosyl thioglycoside **10** was used as a donor (Figure 1) [53,54,55,56]. Glycosylation using both ether-protected donor and acceptor, the so-called “armed glycosylation” [57,58,59], could deliver products **14α**,**β** in high yield but with diminished stereoselectivity (Table 1, entry 1). On the other hand, glycosylation using benzoyl-protected sphingosine **12**, a disarmed acceptor, could provide products **15** in fair yield but slightly improved selectivity (entry 2). This might be attributed to an oxocarbenium ion preformed before the nucleophilic attack by lipid [59]. When a benzyl-protected ceramide **13** [44] was used as an acceptor, only very limited amounts of the glycosylated product **16** were obtained (Table 1, entry 3). The poor yield could be due to the neighboring amido hydrogen donor that decreases the nucleophilicity of the primary alcohol, which has been well documented in the literature [60]. It has been reported that imidate as a donor could achieve excellent yields and α-stereoselectivity in glycosylation [36]. By adopting similar conditions, only the undesired silylated alcohol was obtained, whereas the imidate was consumed (Table 1, entry 4). A similar result was obtained when using ceramide **13** as an acceptor (entry 5); the problem there might be caused by the discrepancy in reactivity between acceptor and donor.

**Scheme 5:**
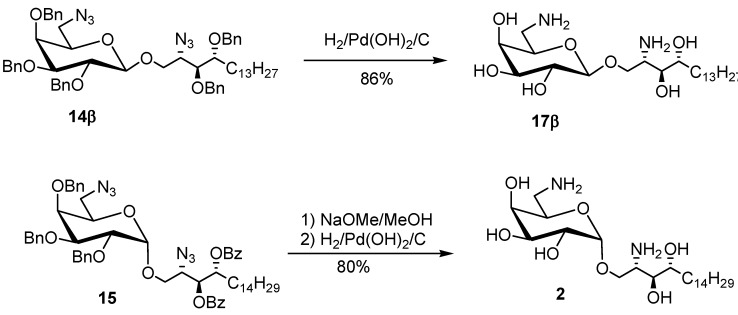
Concomitant removal of benzoyl and benzyl groups using reagent combination.

**Scheme 6:**
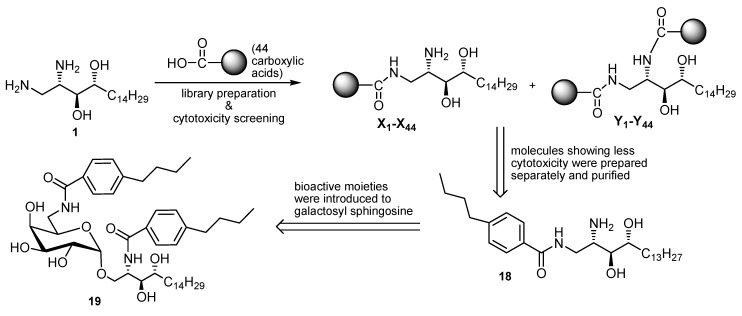
The concept for parallel solution phase synthesis of library for the cytotoxicity screening and further validation for iNKT cell inducing experiment.

Although the concomitant reduction for both benzyl and azido groups of galactosyl sphingosine was difficult [61], compound **14** could be fully deprotected by using the reagent combination of H_2_, MeOH/CHCl_3_, AcOH and Pd(OH)_2_. For example, the β-anomer **14β** was used to test this condition and the deprotected product **17** could be obtained in 86% yield (Figure 6). For comparing with the 18-carbon-based KRN7000, the galactosyl sphingosine **2** was used as another core compound (Scheme 5). Its preparation is relatively straightforward through a stepwise removal of both ester- and ether-protecting groups. Since the more accessible core compound **1** was obtained in sufficient quantity, it provided adequate amounts for further elaboration of amide products (Scheme 6) and for screening cytotoxicities.

**Table 1:** Glycosylation between sphingosine analogs **4**, **12**, **13** and 6-azido galactosyl donors **10**, **11** under armed or disarmed conditions.

Entry	Donor	Acceptor	Time	Product	Yield	α/β
1 ^†^	**10**	**4**	30 min	**14**	95%	51/44
2 ^‡^	**10**	**12**	1 h	**15**	65%	2/1
3 ^§^	**10**	**13**	1 h	**16**	<2%	N.A.
4 ^Ұ^	**11**	**12**	1 h	**15**	N.F.	N.A.
5^ Ұ^	**11**	**13**	1 h	**16**	N.O.	N.A.

^†^ NIS and TfOH (cat.) under 0 °C was used; ^‡^ NIS and TfOH (cat.) under −78 °C→−20 °C was used; ^§^ The presence of the products was confirmed by ESI-MS; ^Ұ^ TMSOTf and co-solvents: Et_2_O/THF 5:1 under −23 °C was used. N.A.: not available; N.O.: not observed; N.F.: not formed but only a silylated acceptor byproduct was obtained.

**Figure 2:**
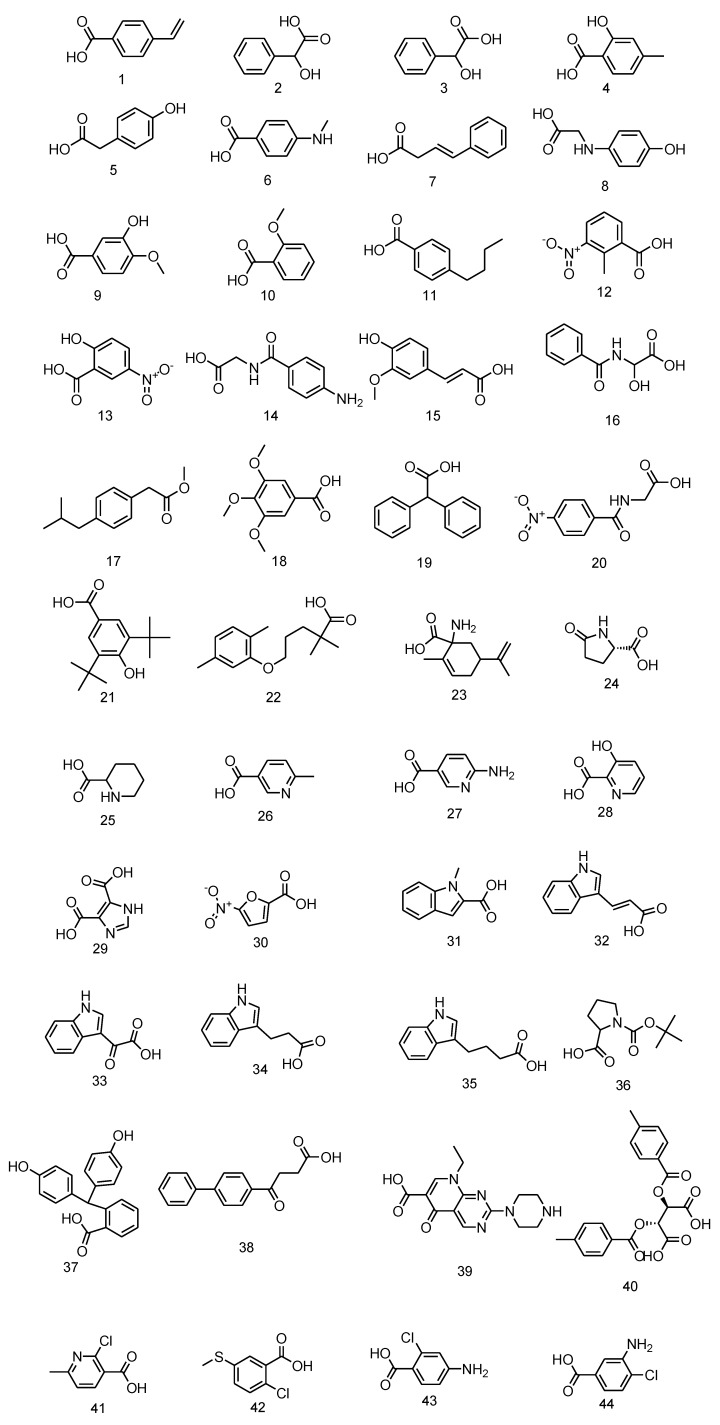
Carboxylic acids used for amide library preparation.

The subsequent library preparation started from core compound **1** (20 mg) by coupling with 44 carboxylic acids using equivalent molarities (Figure 2 and reference [39,40]).

The initial screening for the cytotoxicities of these amide product mixtures was performed by using an MTT assay with normal tissue derived fibroblast cells. Analog **18** showed less cytotoxicity against normal human fibroblasts (50% cell viability *vs*. 0–5% of other analogs in U87 cells).

**Scheme 7:**
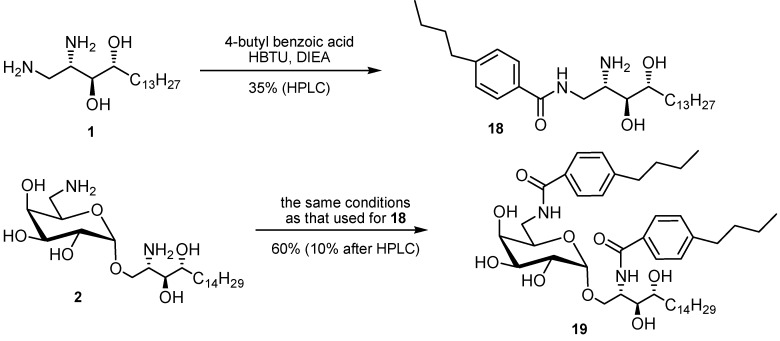
Independent preparation of the potential amide products **18** and **19** followed by purification with HPLC.

The less toxic product mixtures were further examined. These sample mixtures after simple filtration through silica gel were submitted to analysis with ESI-MS. Five product samples showed the expected molecular ion peak patterns, respectively (Supplementary Information). Among them, the 4-butylbenzoic acid derived amide product showing the most significant signals was resynthesized in both its ceramide form **18** and galactosyl ceramide form **19** (Scheme 7).

**Figure 3:**
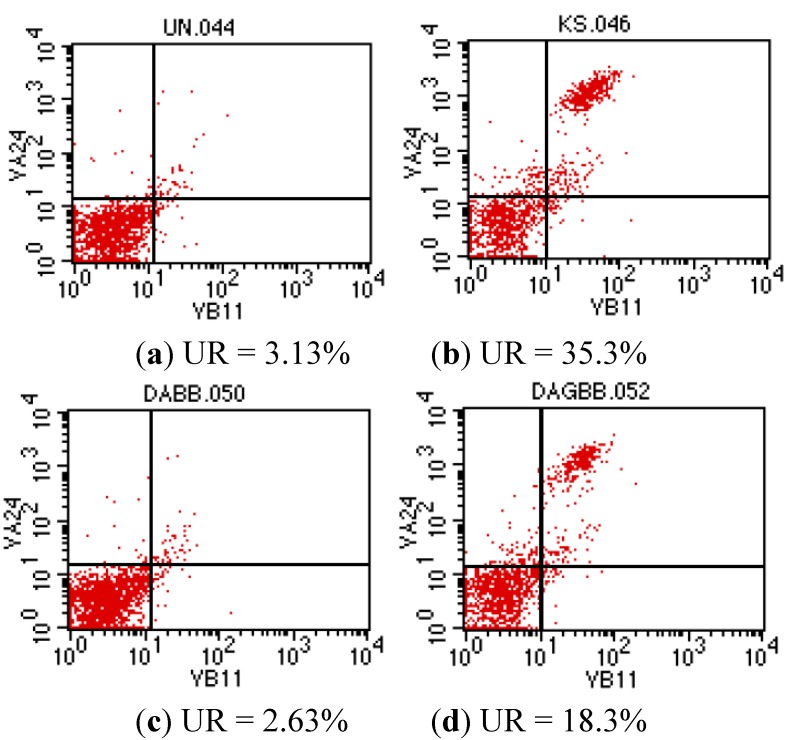
Potencies of analogs **18** and **19** for stimulation of human Vα24+/V-β11+ NKT cell populations. Peripheral blood mononuclear cells (PBMC) from a normal healthy donor were incubated with each individual compound at a final concentration of 100 nM. After 14 days of culture, NKT cell frequencies were determined by flow cytometry. NKT cell frequencies were defined as the percentage of Vα24+/V-β11+ cells among gated lymphocytes in the upper right (UR) corner for each case. Shown here are the profiles of PBMC harvested from 14-day cultures containing (**a**) vehicle alone (DMSO, UN); or (**b**) 100 nM ofα-GalCer (KS); (**c**) analog **18** (DABB); or (**d**) analog **19** (DAGBB), as indicated.

The subsequent validation experiment was performed by MTT assay and flow cytotmetry (Figure 3). Interestingly, α-GalCer analog **19** was Vα24+/Vβ11+ iNKT cell-stimulative but less cytotoxic compound **18** did not show an equivalent activity. This confirmed the important role played by the sugar moieties. Hence, libraries based on galactosyl phytosphingosine analog **2** warrant further study.

## 3. Experimental

### 3.1. General

All reagents and solvents were purchased from Sigma-Aldrich, Mallinckrodt, Acros, Alfa, Tedia, or Fluka. All preparations for nonradioactive compounds were routinely conducted in dried glassware under a positive pressure of nitrogen at room temperature unless otherwise noted. CH_2_Cl_2_, toluene, CH_3_CN, and pyridine were dried over CaH_2_ and MeOH was dried over Mg and distilled prior to reaction. DMF and NEt_3_ were distilled under reduced pressure. Reagents and solvents were of reagent grade. Dimethylaminopyridine (DMAP) was purified through recrystallization from the combination of EtOAc and *n*-hexane before use. The eluents for chromatography: EtOAc, acetone, and *n*-hexane were reagent grade and distilled prior to use; MeOH and CHCl_3_ were reagent grade and used without further purification. NMR spectra including ^1^H-NMR (500 MHz) and ^13^C-NMR (125 MHz, DEPT-135) was measured on a Varian Unity Inova 500 MHz instrument. D-solvents employed for NMR including CD_3_OD, CDCl_3_, C_6_D_6_, and DMSO-d_6_ were purchased from Cambridge Isotope Laboratories, Inc. Low-resolution mass spectrometry (LRMS) was performed on a ESI-MS spectrometry employing VARIAN 901-MS Liquid Chromatography Tandem Mass Q-Tof Spectrometer was performed at the department of chemistry of National Tsing-Hua University (NTHU). High-resolution mass spectrometry (HRMS) was performed using a varian HPLC (Prostar series ESI/APCI) coupled with a Varian 901-MS (FT-ICR Mass) mass detector and triple quadrapole. Elemental analysis was performed using a Foss Heraeus CHN-O-RAPID elemental analysis apparatus. Thin layer chromatography (TLC) was performed with Merck TLC Silica gel 60 F_254_ precoated plates. The starting materials and products were visualized with UV light (254 nm). Further confirmation was carried out by using staining with 5% *p*-anisaldehyde, ninhydrin or ceric ammonium molybdate under heating. Flash chromatography was performed using Geduran Si 60 silica gel (230–400 mesh). Melting points were measured with a MEL-TEMP apparatus and were uncorrected. Flow cytometry was carried out by using a BD FACSCalibur™.

### 3.2. Synthesis of the Compounds

*(2*S*,3*S*,4*R*)-2-Azido-3,4-bis(benzyloxy)heptadecan-1-ol* (**4**): A solution of NaN_3_ (6 g, 90 mmol, 15 eq.) in water (15 mL) and CH_2_Cl_2_ (15 mL) was stirred vigorously at 0 °C. An ice-cold solution of Tf_2_O (5 mL, 30 mmol, 5 eq.) in CH_2_Cl_2_ (5 mL) was added to NaN_3_ (aq.) within 1 min. The solution was vigorously stirred for 2 h and the water phase turned pale yellow. The organic layer was collected and the aqueous phase was further washed with CH_2_Cl_2_ (7 mL × 2). The organic layer combined was washed with saturated Na_2_CO_3_ (15 mL). To a solution of compound **3** (2.8 g, 6.2 mmol) in MeOH (40 mL) was added a solution of K_2_CO_3_ (2 eq., 0.012 mol, 1.7 g) and CuSO_4_·5H_2_O (15 mg, 0.06 mmol, 0.01 eq.) in H_2_O (40 mL), sequentially. The solution of TfN_3 _ described above was added and the color turned to blue-green. The stirring at rt was lasted for 16 h. TLC (MeOH/CHCl_3_ = 1/19) indicated the consumption of starting material **3** (R*_f_* = 0.29) and the formation of the product **4** (R*_f_* = 0.79). The mixture was extracted with EtOAc (40 mL × 3). The organic layer collected was dried with Na_2_SO_4_ and concentrated under reduced pressure. The residue was purified by flash chromatography using silica gel (140 g) with EtOAc/*n*-hexane = 1:19 as eluent to provide a colorless oil with a pleasant odor in 83% yield (2.43 g). ^1^H-NMR (C_6_D_6_): δ 0.91 (t, *J* = 7.0 Hz, 3H, H_aliphatic_), 1.20–1.40 (m, 21H, H_aliphatic_), 1.44–1.56 (m, 2H, H_aliphatic_), 1.64 (bs, 1H, H_OH_), 1.70–1.80 (m, 1H, H_aliphatic_), 3.48 (q, *J* = 5.0 Hz, 1H, H_4_), 3.60–3.76 (m, 4H, H_1_, H_2_, H_3_), 4.37 (d, *J_gem_* = 12.0 Hz, 1H, H_Bn_), 4.46 (t, *J_gem_* =12.0 Hz, 1H, H_Bn_), 4.48 (t, *J_gem_* =12.0 Hz, 1H, H_Bn_), 4.56 (d, *J_gem_* = 11.5Hz, 1H, H_Bn_), 6.98–7.32 (m, 10H, H_Bn_); ^13^C-NMR (C_6_D_6_): δ 14.33 (CH_3_); CH_2_: 23.08, 25.89, 29.37, 29.52, 29.80, 30.16, 30.37, 32.31; 62.67 (CH_2_, C_1_), 63.80 (CH, C_2_), 72.43 (CH_2_, CH_2_Ph), 73.76 (CH_2_, CH_2_Ph), 79.58 (CH, C_4_), 80.03 (CH, C_3_), 127.90 (CH, Ph), 128.03 (CH, Ph), 128.12 (CH, Ph), 128.25 (CH, Ph), 128.32 (CH, Ph), 128.63 (CH, Ph), 128.65 (CH, Ph), 138.52 (C, Ph), 138.79 (C, Ph); LRMS (*m/z*) for C_31_H_47_N_3_O_3_: M (calcd.) = 509.4 (*m/z*), ESI+Q−TOF: M = 509.3 (*m/z*), [M−N_2_−Ph^−^]^+^ = 404.3 (100%), 405.4 (28%), 406.4 (4%); [M+H]^+^ = 510.3 (41%), 511.4 (10%); [M+Na]^−^ = 532.3 (56%), 533.3 (18%), equivalent to the calculated isotopic ratio; analysis (calcd., found for C_31_H_47_N_3_O_3_): C (73.05, 72.74), H (9.29, 9.09), N (8.24, 8.21).

*(2*R*,3*S*,4*S*)-4-Azido-3-(benzyloxy)-2-tridecyltetrahydrofuran* (**6**): Compound **4** (7 mg, 0.013 mmol) was coevaporated with toluene three times, followed by dissolving in CH_2_Cl_2_ (1 mL). Upon the cooling down to −50 °C, pyridine (5μL, 0.06 mmol, 5 eq.) and Tf_2_O (4 μL, 0.03 mmol, 2 eq.) were added sequentially. The reaction was lasted for 30 min. TLC (EtOAc/*n*-hexane = 1:9) indicated the consumption of the starting material 4 (R*_f_* = 0.21) and the formation of the product **6** (R*_f_* = 0.55). CH_2_Cl_2_ (10 mL) was added and the mixture was extracted by saturated aqueous NH_4_Cl (5 mL) and H_2_O (5 mL × 2). The organic layer collected was dried with Na_2_SO_4_ and concentrated under reduced pressure. The residue was purified with flash chromatography using eluents of EtOAc/*n*-hexane = 1:19 and silica gel (4 g) to provide product **6** in 60% yield (3 mg). For analytical purpose, a small amount sample (20 mg) was obtained via another route as described for the preparation of compound **8**. In rare cases, we were able to isolate the triflate **5**. The fragment peaks appeared in ESI-MS spectrum such as 479.3 amu (27%), 493.4 amu (2.4%) and 595.6 amu (2.4%) indicated that the instability of triflate could lead to a number of intermediates. Satisfactory ^1^H-NMR spectra were, however, not available due to the complex patterns. ^1^H-NMR (C_6_D_6_): δ 0.91 (t, *J* = 7.0 Hz, 3H, H_aliphatic_), 1.23–1.36 (m, 21H, H_aliphatic_), 1.38–1.49 (m, 3H, H_aliphatic_), 3.17 (ddd, *J_4,3_*= 6.0, *J_4,5a_* = 5.5,*J_4,5b_* = 5.5 Hz, 1H, H_4_), 3.31 (dd, 1H, *J_3,2_* = 6.5, *J_3,4_* = 6.0 Hz, 1H, H_3_), 3.60 (dd, *J_1a,1b_* = 10.0, *J_1a,2_* = 5.5 Hz, 1H, H_1a_), 3.68 (dd, *J_1b,1a_* = 10.0, *J_1b,2_* = 3.5 Hz, 1H, H_1a_), 3.96 (ddd, *J_2,3_* = 6.5, *J_2,1a_* = 5.5, *J_2,1b_* = 3.5 Hz, 1H, H_2_), 4.21 (d, 1H, *J_gem_* = 11.5 Hz, OC*H*HPh), 4.48 (d, 1H, *J_gem_* = 11.5 Hz, OCH*H*Ph), 7.08–7.11 (m, 1H, Ph), 7.17–7.19 (m, 2H, Ph), 7.29–7.31 (m, 2H, Ph); ^13^C-NMR (C_6_D_6_): δ 14.33 (CH_3_); CH_2_: 23.08, 26.17, 29.79, 30.01, 30.10, 30.13, 32.30, 34.05; 60.69 (CH, C_2_), 69.79 (CH_2_, C_1_), 72.81 (CH_2_, CH_2_Ph), 81.13 (CH, C_4_), 84.03 (CH, C_3_), 128.04 (CH, Ph), 128.11 (CH, Ph), 128.29 (CH, Ph), 128.65 (CH, Ph), 138.08 (C, Ph); LRMS (*m/z*) for C_24_H_39_N_3_O_2_: M (calcd.) = 401.3 (*m/z*); ESI+Q−TOF: M = 401.3 (*m/z*), M^+^−N_2_−Ph+H^−^ = M', [2M'+H]^+^ = 595.59; [M−OTf+H]^+^ = 493.4; [M−OTf−N+H]^+^ = 479.3.

(*2*S*,3*S*,4*R*)-2-Azido-3,4-bis(benzyloxy)heptadecyl-4-methyl benzenesulfonate* (**7**): Before carrying out the tosylation, TsCl was purified by partition between toluene and 10% NaOH (aq). Compound **4** (2.42 g, 4.75 mmol) was azeotropically distilled with toluene for three times, followed by dissolving in CH_2_Cl_2_ (75 mL) under N_2_ at 0 °C. Pyridine (75 mL) and *p*-TsCl (1.81 g, 9.5 mmol) were added, sequentially, and the mixture was stirred for 10 min, followed by stirring at rt for 16 h. TLC (EtOAc/*n*-hexane = 1:9) indicated the consumption of the starting material **4** (R*_f_* = 0.19) and the formation of the product 7 (R*_f_* = 0.40). Following the addition of H_2_O (100 mL), the aqueous phase was extracted with CH_2_Cl_2_ (40 mL × 3). The organic layer collected was dried with Na_2_SO_4_ and concentrated under reduced pressure. The residue was purified using flash chromatography with eluents of EtOAc/*n*-hexane = 1/19 and silica gel (100 g) to provide colorless oil **7** in 93% yield (2.94 g). ^1^H-NMR (C_6_D_6_): δ 0.91 (t, *J* = 7.0 Hz, 3H, H_aliphatic_), 1.19–1.38 (m, 22H), 1.38–1.46 (m, 1H), 1.61–1.68 (m, 1H), 1.79 (s, 3H), 3.48 (dd, *J_3,4_* = 5.0, *J_3,2_* = 5.0 Hz, 1H, H_3_), 3.53 (ddd, 1H, *J_4,5a_* = 7.0, *J_4,5b_* = 6.5, *J_4,3_* = 5.0 Hz, 1H, H_4_), 3.80 (ddd, *J_2,1a_* = 7.5, *J_2,3_* = 5.0, *J_2,1b_* = 2.5 Hz, 1H, H_2_), 4.28 (dd, *J_1a,1b_* = 10.5, *J_1a,2_* = 7.5 Hz, 1H, H_1a_), 4.31 (d, 1H, *J_gem_* = 12.0 Hz, OC*H*HPh), 4.35 (d, 1H, *J_gem_* = 12.0 Hz, OCH*H*Ph), 4.39 (s, 2H, 2 × OC*H*HPh), 4.46 (dd, *J_1b,1a_* = 10.5, *J_1b,2_* = 2.5 Hz, 1H, H_1b_), 6.63–6.64 (m, 2H, Ph), 7.06–7.13 (m, 4H, Ph), 7.16–7.21 (m, 4H, Ph), 7.26–7.27 (m, 2H, Ph), 7.74–7.57 (m, 2h, Ph); ^13^C-NMR (C_6_D_6_): δ 14.33 (CH_3_); 21.11 (CH_3_); CH2: 23.07, 25.39, 29.79, 30.01, 30.09, 30.14, 30.27, 32.29; 61.60 (CH, C_2_), 69.92 (CH_2_, C_1_), 72.26 (CH_2_, CH_2_Ph), 73.52 (CH_2_, CH_2_Ph), 78.75 (CH, C_4_), 79.67 (CH, C_3_), 127.87 (CH, Ph), 128.04 (CH, Ph), 128.23 (CH, Ph), 128.29 (CH, Ph), 128.62 (CH, Ph), 129.86 (CH, Ph), 133.80 (C, tosyl), 138.08 (C, Ph), 138.64 (C, Ph), 144.44 (C, tosyl); LRMS for C_38_H_53_N_3_O_5_S: M (calcd.) = 663.4 (*m/z*), MW = 663.9, ESI+Q−TOF: M = 663.3 (*m/z*), [M+Na]^+^ = 686.3.

*[(2*S*,3*S*,4*R*)-1,2-Diazidoheptadecane-3,4-diyl)bis(oxy)bis(methylene]dibenzene* (**8**): An aqueous solution of LiN_3_ (10.55 g, 43.1 mmol, 20% wt in water) was azeotropically distilled with DMF (2 mL) under reduced pressure for two times. The residue was dissolved in DMF (75 mL) and transferred to a two-necked bottom flask containing a solution of starting material **7** (2.86 g, 4.31 mmol) in DMF (75 mL) under N_2_ at r.t. The mixture was then stirred at 80 °C for 2h. TLC (EtOA/*n*-hexane = 1:9) indicated the consumption of the starting material **7** (R*_f_* = 0.42) and the formation of the product 8 (R*_f_* = 0.55). The mixture was transferred to a funnel for partition between H_2_O (75 mL) and EtOAc (75 mL). The organic layer separated was dried with Na_2_SO_4_ and filtered through a Celite pad. The filtrate was concentrated under reduced pressure. The residue obtained was purified with flash chromatography on silica gel (110 g) using EtOAc/*n*-hexane 1:39 as eluent to provide **8** as a colorless oil in 80% yield (1.81 g) and compound **6** in 9% yield (155 mg). ^1^H-NMR (C_6_D_6_): δ 0.91 (t, *J* = 6.5 Hz, 3H, H_aliphatic_), 1.22–1.38 (m, 21H, H_aliphatic_), 1.38–1.52 (m, 2H, H_aliphatic_), 1.66–1.78 (m, 1H, H_aliphatic_), 3.17 (d, *J* = 5.0 Hz, 2H, H_3_, H_4_), 3.46-3.56 (m, 3H, H_1_, H_2_), 4.35 (d, *J_gem_* = 11.5 Hz, 1H, OC*H*HPh), 4.38 (d, *J_gem_* = 11.5 Hz, 1H, OC*H*HPh), 4.41 (d, *J_gem_* = 11.5 Hz, 1H, OC*H*HPh), 4.51 (d, *J_gem_* = 11.5 Hz, 1H, OC*H*HPh), 7.07–7.12 (m, 2H, Ph), 7.16–7.20 (m, 4H, Ph), 7.21–7.25 (m, 2H, Ph), 7.26–7.27 (m, 2H, Ph); ^13^C-NMR (C_6_D_6_): δ 14.33 (CH_3_), CH_2_: 23.08, 25.66, 29.79, 30.03, 30.09, 30.15, 30.28, 32.30; 52.22 (CH_2_, C_1_), 62.69 (CH, C_2_), 72.20 (CH_2_, OCH_2_Ph), 73.75 (CH_2_, OCH_2_Ph), 79.05 (CH, C_4_), 79.82 (CH, C_3_), 127.97 (CH, Ph), 128.07 (CH, Ph), 128.13 (CH, Ph), 128.22 (CH, Ph), 128.29 (CH, Ph), 128.63 (CH, Ph), 128.65 (CH, Ph), 138.30 (C, Ph), 138.66 (C, Ph); LRMS for C_31_H_46_N_6_O_2_: M (calcd.) = 534.3 (*m/z*), MW = 534.7, ESI+Q−TOF: M = 534.3 (*m/z*), [M+Na]^+^ = 557.3 (100%), 558.3 (42%), 559.3 (4%), equivalent to the calculated isotopic ratio; analysis (calcd., found for C_31_H_46_N_6_O_2_): C (69.63, 69.40), H (8.67, 8.53), N (15.72, 15.83).

*(2*S*,3*S*,4*R*)-1-Amino-2-azidoheptadecane-3,4-diol* (**9**): Starting material **8** (38 mg, 0.071 mmol) after coevaporation with toluene for three times was dissolved in CH_2_Cl_2_ (1 mL) under N_2_. The mixture was cooled down to −78 °C. BCl_3_/CH_2_Cl_2_ (1 M, 35 μL, 0.35 mmol, 5 eq.) was added within 2 min. The mixture was stirred at −78 °C for 2 h followed by slow warming to r.t. within 20 min and the stirring was lasted for further 10 h. TLC (EtOAc/*n*-hexane = 1:9) indicated the consumption of the starting material 8 (R*_f_* = 0.75) and the formation of the product **9** (R*_f_* = 0.07). Upon the addition of MeOH (0.1 mL), the mixture became an opaque light brown solution. It was then concentrated under reduced pressure to provide a yellow oily residue. The purification of the residue using flash chromatography with MeOH-CHCl_3_ 1:9 as eluent and silica gel (1 g) afforded product **9** in 60% yield (13 mg). ^1^H-NMR (CD_3_OD): δ 0.89 (t, *J* = 7.0 Hz, 3H, H_aliphatic_), 1.24–1.43 (m, 22H, H_aliphatic_), 1.50–1.62 (m, 1H, H_aliphatic_), 1.68–1.78 (m, 1H, H_aliphatic_), 3.11 (dd, *J_1a,1b_* = 13.0 Hz, *J_1a,2_* = 8.0 Hz, 1H, H_1a_), 3.17 (dd, *J_1b,1a_* = 13.0, *J_1b,2_* = 3.5 Hz, 1H, H_1b_), 3.49 (td, *J_2,1a_* = 8.0, *J_2,3_* = 8.0, *J_2,1b_* = 3.5 Hz, 1H, H_2_), 3.67 (dd, *J_3,2_* = 8.0, *J_3,4_* = 3.0 Hz, 1H, H_3_), 3.92 (ddd, *J* = 8.5, *J* = 4.0, *J_4,3_* = 3.0 Hz, 1H, H_4_); ^13^C-NMR (CD_3_OD): δ 14.43 (CH_3_), CH_2_: 23.73, 26.61, 30.47, 30.79, 33.07, 34.89; 40.18 (CH_2_, C_1_), 61.88 (CH, C_2_), 72.92 (CH, C_4_), 76.17 (CH, C_3_); LRMS for C_17_H_38_N_4_O_2_, M (calcd.) = 328.3 (*m/z*), ESI+Q-TOF: M = 328.4 (*m/z*), [M+H]^+^ = 329.4 (100%), 330.4 (22%), equivalent to the calculated isotopic ratio (100%:18.9%).

*(2*S*,3*S*,4*R*)-1,2-Diaminoheptadecane-3,4-diol* (**1**): Starting material **8** (812 mg, 1.5 mmol) was distilled azeotropically with toluene for three times followed by dissolving in CH_2_Cl_2_ (20 mL) under N_2_. The mixture was cooled down to −78 °C. BCl_3_ (15 mL, 15 mmol, 1 M in CH_2_Cl_2_, 10 eq.) was added within 3 min. The mixture was stirred at −78 °C for 2 h, followed by warming to rt within 20 min and the stirring was lasted for further 10 h. TLC (MeOH/CHCl_3_ = 2:8) indicated the consumption of the starting material **8** (R*_f_* = 0.88) and the formation of the product **1** (R*_f_* = 0.05). Upon the addition of MeOH (5 mL), the pale yellow solution became a milky white mixture. It was then concentrated under reduced pressure to provide a pale yellow solid. After recrystallization from hot CHCl_3_, the amorphous precipitate was washed with cold *n*-hexane and dried under reduced pressure to provide the yellow solid **1** in quantitative yield (445 mg). The chemical shifts of protons from C_1_ to C_4_ in the ^1^H-NMR were slightly upfield. Interestingly, the two ammonium protons were no longer observable between 8 and 9 ppm, indicating the presence of a neutral amine rather than the ammonium ion. The protons of the ammonium complex with HCl could be observed in ^1^H-NMR. By contrast, no peaks could be found in ESI-MS. HCl is easier to evaporate during the electrospraying step and thereby only the neutral amino form emerged as the base peak, 303.4 (*m/z*). In contrast, a substantial amount of the ammonium hydroxide form would be preserved during ESI thereby appearing as the base peak. The patterns of peak clustering around 389.3 (*m/z*) implied the presence of a chloro-containing molecular ion. mp: 96–100 °C, ^1^H-NMR (CD_3_OD): δ 0.88 (t, *J* = 7.0 Hz, 3H, H_aliphatic_), 1.20–1.47 (m, 22H, H_aliphatic_), 1.49–1.62 (m, 1H, H_aliphatic_), 1.64–1.77 (m, 1H, H_aliphatic_), 3.30 (dd, *J_1a,1b_* = 14.5, *J_1a,2_* = 4.0 Hz, 1H, H_1a_), 3.49 (dd, *J_1b,1a_* = 14.5, *J_1b,2_* = 5.0 Hz, 1H, H_1b_), 3.66 (ddd, *J_4,5a_* = 8.0, *J_4,5b_* = 8.0, *J_4,3_* = 3.0 Hz, 1H, H_4_), 3.77 (dd, *J_3,2_* = 7.0, *J_3,4_* = 3.0 Hz, 1H, H_3_), 3.83 (ddd, *J_2,3_* = 7.0, *J_2,1b_* = 5.0, *J_2,1a_* = 4.0 Hz, 1H, H_2_), 8.26 (bs, 1H, NH), 8.47 (bs, 1H, NH); ^13^C-NMR (CD_3_OD): δ 14.44 (CH_3_), CH_2_: 23.69, 26.57, 30.44, 30.73, 30.76, 33.03, 34.76; 39.15 (CH_2_, C_1_), 51.87 (CH, C_2_), 73.76 (CH, C_4_), 74.01 (CH, C_3_); LRMS for C_17_H_38_N_2_O_2_: M = 302.3 (calcd.); ESI+Q-TOF: M = 302.4 (*m/z*), [M+H]^+^ = 303.4 (100%), 304.4 (20%), equivalent to the calculated isotopic ratio;[M+Na]^+^ = 325.3, [2M+H]+ = 605.6. A sample was further purified with anionic ion exchange resin (OH^-^). Following the gentle stirring of the mixture in MeOH for 2 min, it was filtered by paper. The filtrate collected was concentrated to provide white solid for subsequent analysis with ^1^H-NMR and ESI-MS. ^1^H-NMR (CD_3_OD): δ 0.89 (t, *J* = 7.0 Hz, 3H, H_aliphatic_), 1.20–1.41 (m, 24H, H_aliphatic_), 1.45–1.65 (m, 1H, H_aliphatic_), 1.70–1.76 (m, 1H, H_aliphatic_), 2.69 (bs, 1H), 2.89 (bs, 2H), 3.30 (bs, 1H), 3.45–3.52 m, 1H), 3.77 (dd, *J_3,2_* = 7.0, *J_3,4_* = 3.0 Hz, 1H, H_3_); LRMS for C_17_H_38_N_2_O_2_: ESI+Q−TOF: M = 302.4 (*m/z*), [M+H]^+^ = 303.4 (25%), 304.4 (5%), [M+H_2_O+Na]^+^ = 343.4 (100%), 344.4 (29%), 345.4 (4), roughly equivalent to the calculated isotopic ratio (100:18.4:1.6); [M+2H_2_O+K]^+^ = 377.4.

*2-Azido-3,4-di-*O*-benzyl-1-*O*-(6-azido-2,3,4-tri-*O*-benzyl-α-D-galactopyranosyl)-D-ribo-heptadecan-1-ol* (**14α**): To a solution of donor 10 (954 mg, 1.64 mmol) and acceptor **4** (601 mg, 1.12 mmol) in CH_2_Cl_2_ (10 mL) under N_2_ 4Å molecular sieve (1.8 g) was added. The stirring at rt was continued for 30 min, followed by cooling down to 0 °C. To the mixture was then added *N*-iodosuccinimide (1.56 g, 7.0 mmol) and TfOH (13 mg, 0.09 mmol), prepared by dissolving TfOH (0.5 mL) in CH_2_Cl_2_ (10 mL). The stirring was lasted for 30 min. TLC (EtOAc/*n*-hexane = 1:9) indicated the consumption of the acceptor **4** (R*_f_* = 0.22) and the formation of the product **14α** (R*_f_* = 0.50) and product **14β** (R*_f_* = 0.34). When adding CH_2_Cl_2_ (20 mL) and saturated aqueous Na_2_S_2_O_3_ (20 mL) for partition, the solution turned from dark violet to white. The organic layer was further extracted with saturated aqueous NaHCO_3_ (20 mL). After drying the organic layer with Na_2_SO_4_, the solution was filtered through celite pad and concentrated under reduced pressure. The residue obtained was purified by flash chromatography with EtOAc/*n*-hexane = 1:19 as eluent to provide compound**14α** in 51% yield (583 mg) and compound **14β** in 44% yield (501 mg), both of oily appearance. ^1^H-NMR (C_6_D_6_): δ 0.91 (t, 3H, CH_3_), 1.22–1.36 (m, 20H, CH_2_), 1.32–1.48 (m, 1H, CHH), 1.50–1.58 (m, 1H, CHH), 1.58–166 (m, 1H, CHH), 1.84–1.91 (m, 1H, CHH), 2.74 (dd, *J_6'a,6'b_* = 12.5, *J_6'a,5_* = 4.0 Hz, 1H, H-6'a), 3.45 (s, 1H, H-4'), 3.47 (dd, *J_6'b,6'a_* = 12.5, *J_6'b,5__’_* = 8.0 Hz, 1H, H-6'b), 3.72–3.78 (m, 3H, H_1a_, H_2_, H_4_), 3.82 (dd, *J_5_*_',6b_ = 8.0, *J_5_*_',6a_ = 4.0 Hz, 1H, H_5'_), 3.87 (t, *J* = 4.3 Hz, 1H, H_3_), 4.04 (dd, *J_2',3'_* = 10.5, *J_2',1'_* = 3.5 Hz, 1H, H_2'_), 4.16 (dd, *J_3',2'_* = 10.5, *J_3',4'_* = 4.0 Hz, 1H, H_3'_), 4.20 (dd, *J_1b,1a_* = 13.0, *J_1b,2_* = 6.0 Hz,1H, H_1__b_), 4.42–4.48 (m, 3H, CH_2_Ph), 4.57–4.64 (m, 4H, CH_2_Ph), 4.71 (d, *J* = 11.5 Hz, 1H, CH_2_Ph), 4.78 (d, *J* = 11.5 Hz, 1H, CH_2_Ph), 4.88 (d, *J_1',2'_* = 3.5 Hz, 1H, H_1'_), 4.98 (d, *J* = 11.5, 1H, CH2Ph), 7.00–7.05 (m, 1H, H_Bn_), 7.08–7.12 (m, 6H, H_Bn_), 7.16–7.21 (m, 8H, H_Bn_), 7.28–7.30 (m, 4H, H_Bn_), 7.31–7.33 (m, 2H, H_Bn_), 7.34–7.37 (m, 4H, H_Bn_); ^13^C-NMR (C_6_D_6_): δ 14.33 (CH_3_), CH_2_: 23.08, 26.01, 29.79, 30.15, 30.22, 30.35, 32.30, 51.84; 62.43 (CH), 68.69 (CH_2_), 70.91 (CH), 72.26 (CH_2_Ph), 73.36 (CH_2_Ph), 73.62 (CH_2_Ph), 74.01 (CH_2_Ph), 75.04 (CH_2_Ph), 76.22 (CH), 77.08 (CH), 78.88 (CH), 79.09 (CH), 79.98 (CH), 98.75 (CH),127.66 (CH, Ph), 127.80 (CH, Ph), 128.00 (CH, Ph), 128.19 (CH, Ph), 128.29 (CH, Ph), 128.44 (CH, Ph), 128.49 (CH, Ph), 128.57 (CH, Ph), 128.62 (CH, Ph), 138.85 (C, Ph), 138.99 (C, Ph), 139.15 (C, Ph), 139.19 (C, Ph), 139.30 (C, Ph); LRMS for C_58_H_74_N_6_O_7_: M (calcd.) = 966.6 (*m/z*), ESI+Q−TOF: M = 966.6 (*m/z*), [M−H+H]^+^ = 966.6, M' = M−H^+^+NH_4_^+^, [2M'+H]+ = 1967.0; analysis (calcd., found for C_58_H_74_N_6_O_7_): C (72.02, 72.11), H (7.71, 7.42), N (8.69, 8.66).

*2-Azido-3,4-di-O-benzyl-1-*O*-(6-azido-2,3,4-tri-*O*-benzyl-β-D-galactopyranosyl)-D-ribo-heptadecan-1-ol* (**14β**): ^1^H-NMR (C_6_D_6_): δ 0.91 (t, 3H, CH_3_), 1.19–1.35 (m, 20H, CH_2_), 1.36–1.46 (m, 1H, CHH), 1.48–1.56 (m, 1H, CHH), 1.58–164 (m, 1H, CHH), 1.83–1.90 (m, 1H, CHH), 2.70 (dd, *J_6'a,6'b_* = 12.5, *J_6'a,5_* = 4.0 Hz, 1H, H_6'a_), 2.94 (dd, *J_3_*_,__2_ = 7.5, *J_3,4_* = 4.0 Hz, 1H, H_3_), 3.22 (dd, *J_3',2'_* = 9.5 Hz, *J_3',4'_* = 3.0 Hz, 1H, H_3'_), 3.28 (dd, *J_4',3'_* = 3.0, *J_4',5'_* = 2.5 Hz, 1H, H_4'_), 3.38 (dd, *J_6'b,6'a_* = 12.5, *J_6'b,5'_* = 7.5 Hz, 1H, H_6'b_), 3.73 (ddd, *J_2,3_* = 7.5, *J_2,1a_* = 3.0, *J_2,1b_* = 2.5 Hz, 1H, H_2_), 3.80 (ddd, *J_5',6b'_* = 7.5, *J_5',6a' _* = 4.0, *J_5',4'_* = 2.5 Hz, 1H, H_5'_), 3.82–3.86 (m, 1H, H_4_), 3.94 (dd, *J_1a,__1b_* = 10.5, *J_1a, __2_* = 2.5 Hz, 1H, H_1a_), 4.06 (dd, *J_2',3'_* = 9.5, *J_2',1'_* = 7.5 Hz, 1H, H_2'_), 4.26 (d, *J_1',2'_* = 7.5 Hz, 1H, H_1'_), 4.39 (dd, *J_1__b,__1a_* = 10.5, *J_1__b,__2_* = 6.5 Hz, 1H, H_1__b_), 4.42 (dd, *J* = 12.0 Hz, 1H, CH_2_Ph), 4.44 (dd, *J* = 12.0 Hz, 1H, CH_2_Ph), 4.53 (dd, *J* = 12.0 Hz, 1H, CH_2_Ph), 4.55 (dd, *J* = 11.5 Hz, 1H, CH_2_Ph), 4.64–4.70 (m, 3H), 4.76 (d, *J* = 11.0 Hz, 1H, CH_2_Ph), 4.94 (d, *J* = 11.5 Hz, 1H, CH_2_Ph), 5.09 (d, *J* = 11.5 Hz, 1H, CH_2_Ph), 7.07–7.13 (m, 7H, H_Bn_), 7.16–7.21 (m, 8H, H_Bn_), 7.25–7.26 (m, 2H, H_Bn_), 7.32–7.34 (m, 4H, H_Bn_), 7.36–7.37 (m, 2H, H_Bn_), 7.45–7.46 (m, 2H, H_Bn_); ^13^C-NMR (C_6_D_6_): δ 14.33 (CH_3_), CH_2_: 23.08, 25.95, 29.79, 30.11, 30.15, 30.22, 30.30, 32.30, 51.40; 62.64 (CH), 69.15 (CH_2_), 72.12 (CH_2_Ph), 73.42 (CH_2_Ph), 73.86 (CH_2_Ph),74.20 (CH), 74.75 (CH), 74.89 (CH_2_Ph), 75.29 (CH_2_Ph), 78.93 (CH), 79.82 (CH), 82.01 (CH), 104.08 (CH), 127.61 (CH, Ph), 127.70 (CH, Ph), 127.81 (CH, Ph), 127.92 (CH, Ph), 128.00 (CH, Ph), 128.19 (CH, Ph), 128.29 (CH, Ph), 128.38 (CH, Ph), 128.45 (CH, Ph), 128.57 (CH, Ph), 128.65 (CH, Ph), 138.82 (C, Ph), 139.07 (C, Ph), 139.13 (C, Ph), 139.59 (C, Ph).

*2-Azido-3,4-di-*O*-benzoyl-1-*O*-(6-azido-2,3,4-tri-*O*-benzyl-α-D-galactopyranosyl)-D-ribo-octadecan-1-ol* (**15α**,**β**): A mixture of donor **10** (50 mg, 0.86 mmol) and acceptor **12** (79 mg, 0.14 mmol, 1.5 eq.) was azeotropically distilled with toluene (10 mL) for three times. CH_2_Cl_2_ (1.5 mL) and powdered 4 Å MS (150 mg) were added, sequentially, under N_2_. After stirring for 30 min, the mixture was moved to an ice bath. Following the addition of NIS (126 mg, 0.56 mmol, 6.2 eq.), the flask was stirred at −78 °C for 5 min. TfOH (0.56 μL, 0.006 mmol, 0.1 eq.) was added, while the mixture turned dark red. The stirring was warmed to −20 °C during 10 min. After 1 h, TLC (EtOAc/*n*-hexane = 1:9) indicated the formation of the products 15α,β (R*_f_* = 0.66) and the consumption of the acceptor 12 (R*_f_* = 0.26) and the donor 10 (R*_f_* = 0.66). The mixture were filtered through a Celite pad and the filtrate obtained was concentrated under reduced pressure. The residue was dissolved in EtOAc and treated with Na_2_S_2_O_3(aq) _(3 mL), followed by extraction with NaHCO_3(aq) _ (5 mL). The organic phase was collected and dried with Na_2_SO_4_, followed by filtration with a Celite pad. The filtrate was concentrated under reduced pressure and the resultant residue was purified by flash chromatography using eluents of EtOAc/*n*-hexane = 1:39 to provide the colorless product mixtures 15 in 65% yield (60 mg) and α:β ratio of 2:1. Each of the two anomers could be collected in its pure form from the fractions. Data of **15α**: ^1^H-NMR (CDCl_3_): δ 0.86 (t, *J* =7.0 Hz, 3H, H_aliphatic_), 1.19–1.40 (m, 24H, H_aliphatic_), 1.83–1.85 (m, 2H, H_aliphatic_), 2.91(dd, *J*_6a',6b'_= 12.5 Hz, *J*_6a',5'_ = 5.0 Hz, 1H, H-6a'), 3.43 (dd, *J*_6__b',6__a'_ = 12.5, *J*_6__b',5'_ = 8.5 Hz, 1H,H-6b'), 3.68 (dd, *J*_1a,__1b_ = 10.5, *J*_1a,__2_ = 7.5 Hz, 1H, H-1a), 3.73 (bs, 1H, H-4'), 3.82 (dd, *J*_5',__6b'_ = 8.5, *J*_5',__6a'_ = 5.0 Hz, 1H, H-5'), 3.90 (dd, *J*_3',__2'_ = 10.0, *J*_3',__4'_ = 3.0 Hz, 1H, H-3'), 3.98 (dd, *J*_2',__3'_ = 10.0, *J*_2',__1'_ = 4.0 Hz, 1H, H-2'), 4.00 (dd, *J*_2,__1a_ = 7.5, *J*_2,__1b_ = 3.0 Hz, 1H, H-2), 4.03 (dd, *J*_1b,__1a_ = 10.5, *J*_1b,__2_ = 3.0 Hz, 1H, H-1b), 4.55 (d, 1H,*J* = 11.5Hz, CH_2_Ph), 4.62 (d, 1H, *J* = 12.5 Hz, CH_2_Ph), 4.67–4.70 (d, *J* = 11.5 Hz, 2H, CH_2_Ph), 4.80 (d, 1H, *J*_1',__2'_ = 4.0Hz, H_1'_), 4.83 (d, 1H,*J* = 11.5 Hz, CH_2_Ph), 4.95 (d, 1H, *J* = 11.5 Hz, CH_2_Ph), 5.50–5.53 (m, 2H, H-3+H-4), 7.14–7.44 (m, 19H, ArH), 7.52–7.86 (m, 2H, ArH), 7.96–8.01 (m, 4H, ArH); ^13^C-NMR (CDCl_3_): δ 14.11 (CH_3_), CH_2_: 22.68, 25.31, 29.35, 29.38, 29.41, 29.50, 29.58, 29.64, 29.67, 29.69, 29.87, 31.92, 51.40; 61.36 (CH), 68.54 (CH_2_) , 70.32 (CH), 72.86 (CH), 72.90 (CH), 73.12 (CH_2_, CH_2_Ph), 73.65 (CH_2_, CH_2_Ph), 74.58 (CH_2_, CH_2_Ph), 75.20 (CH), 76.21 (CH), 78.42 (CH), 98.80 (CH); arom. CH: 127.55, 127.59, 127.83, 127.89, 128.27, 128.38, 128.43, 128.47, 128.56, 129.73, 129.87, 133.16, 133.48; arom. quaternary C: 129.32, 129.79, 138.10, 138.51, 138.70; 165.15 (CO), 165.73 (CO). Data of **15β**: ^1^H-NMR (CDCl_3_): δ 0.86 (t, 3H, *J* = 7.0Hz, H_aliphatic_), 1.16–1.43 (m, 22H, H_aliphatic_), 1.57 (bs, 2H, H_aliphatic_), 1.78–1.86 (m, 2H, H_aliphatic_), 2.82 (dd, *J*_6a',6b'_ = 12.0 Hz, *J*_6a',5'_ = 4.5 Hz, 1H, H_6a'_), 3.39 (dd, *J*_6__b',6__a'_ = 12.0, *J*_6__b',5'_ = 7.5 Hz, 1H, H-6b'), 3.43 (dd, *J*_5',__6b'_ = 7.5, *J*_5',__6a'_ = 4.5 Hz, 1H, H-5'), 3.47 (dd, *J*_3',__2'_ = 9.5, *J*_3',__4'_ = 2.5 Hz, 1H, H-3'), 3.65 (d, *J* = 1.0 Hz, 1H, H-4'), 3.83 (dd, *J*_2',__3'_ = 9.5, *J*_2',__1'_ = 8.0 Hz, 1H, H-2'), 3.90 (dd, *J*_1a,__1b_ = 10.5, *J*_1a,__2_ = 1.5 Hz, 1H, H-1a), 3.96 (bs, 1H, H-2), 4.14 (dd, *J*_1b,__1a_ = 10.5, *J*_1b,__2_ = 8.5 Hz, 1H, H-1b), 4.36 (d, *J*_1',__2'_ = 8.0 Hz, 1H, H_1_'), 4.58 (d, *J* = 11.5 Hz, 1H, CH_2_Ph), 4.70 (d, *J* = 11.5 Hz, 1H, CH_2_Ph), 4.73 (d, *J* = 10.5 Hz, 1H, CH_2_Ph), 4.77 ( d, *J* = 10.5 Hz, 1H, CH_2_Ph), 4.89 (d, 1H, *J* = 11.0 Hz, H_1_), 4.96 (d, 1H, *J* = 11.5 Hz, CH_2_Ph), 5.48–5.54 (m, 2H, H-3+H-4), 7.20–7.36 (m, 14H, ArH), 7.36–7.43 (m, 5H, ArH), 7.51–7.58 (m, 2H, ArH), 7.96–8.00 (m, 4H, ArH); ^13^C-NMR (CDCl_3_): δ 14.07 (CH_3_), CH_2_: 22.67, 25.28, 29.34, 29.39, 29.49, 29.57, 29.62, 30.10, 31.90, 51.0; 61.42 (CH), 68.50 (CH_2_), 72.81 (CH), 72.97 (CH), 73.42 (CH_2_, CH_2_Ph), 73.53 (CH), 74.20 (CH), 74.34 (CH_2_, CH_2_Ph), 75.30 (CH_2_, CH_2_Ph), 79.23 (CH), 81.89 (CH), 103.43 (CH), arom. CH: 127.47, 127.58, 127.71, 127.84, 127.98, 128.22, 128.33, 128.43, 128.46, 129.74, 129.92, 133.12, 133.34; arom. quaternary C:129.39, 129.78, 138.05, 138.22,138.63; 165.05 (CO), 165.71 (CO); LRMS for C_59_H_72_N_6_O_9_: M (calcd.) = 1008.5 (*m/z*), ESI+Q−TOF: M = 1008.6 (*m/z*), [M+Na]^+^ = 1031.6 (3.67%), 1032.6 (2.54%), approximately equivalent to the calculated isotopic ratio (100%:65%). Coupling of 11 and 12 afforded only the undesired silylated product. ^1^H-NMR (CDCl_3_): δ 0.10 (s, 9H, CH_3_), 0.90 (t, 3H, CH_3(aliphatic)_), 1.15–1.50 (m, 24H, H_aliphatic_), 1.80–2.00 (m, 2H, H_aliphatic_), 3.75–4.00 (m, 3H), 5.46–5.56 (m, 2H, H_3_, H_4_), 7.40–7.50 (m, 4H, ArH), 7.53–7.63 (m, 2H, ArH), 7.98–8.00 (m, 4H, ArH). Attempt to synthesize compound **16** by coupling **10** and **13**. Anal. C_83_H_124_N_4_O_8_, M (calcd.) = 1304.9 (*m/z*); ESI+Q−TOF: M = 1304.8 (*m/z*), [M+Na]^+^ = 1327.8 (8.7%), 1328.6 (7.8%), 1329.5 (3.6%), approximately equivalent to the calculated isotopic ratio (100%:91.5%:43.0%).

*2-Amino-1-*O*-(6-amino-β-D-galactopyranosyl)-D-ribo-heptadecan-1,3,4-ol* (**17β**): To a solution of β-anomer **14β** (40 mg, 0.41 mmol) in CHCl_3_ (0.5 mL) was added MeOH (2 mL). AcOH (20 μL) and Pd(OH)_2_ (81 mg) were added to the stirred mixture, sequentially. It was then sealed with septa and parafilm. The glassware was evacuated with syringe, followed by charging with hydrogen gas provided by a balloon. Repeating the procedure twice, a mixed solution of MeOH/CHCl_3_ (1 mL, 4/1) was added to compensate for the solvent reduced by evaporation. The mixture was then stirred under an atmosphere of a balloon filling with hydrogen for 23 h. TLC (NH_3_/MeOH/CHCl_3_ = 1/5/5) indicated the consumption of the starting material **14β** (R*_f_* = 0.95) and the formation of the product **17β** (R*_f_* = 0.14). The mixture was then filtered through a Celite pad, followed by washing with CHCl_3_ and MeOH, sequentially. The filtrates were combined and concentrated under reduced pressure to provide an off-white solid which was followed by washing with CHCl_3_ to remove some colored impurities. The wet solid was filtered and collected. The residue was dried under reduced pressure to afford a white solid in 86% yield (16 mg). Recrystallization of a sample from water was unsuccessful. Instead, after concentration under reduced pressure, the solid become pale yellow. ^1^H-NMR (D_2_O): δ 0.82 (bs, 3H, CH_3_), 1.23 (bs, 22H, CH_2_), 1.47 (bs, 1H), 1.72 (bs, 1H), 3.28 (bs, 2H), 3.54 (bs, 2H), 3.69 (bs, 2H), 3.81 (bs, 1H), 3.93 (bs, 2H), 4.05 (bs, 1H), 4.13 (bs, 1H), 4.50 (bs, 1H); ^13^C-NMR (125 MHz, D_2_O): δ 14.10 (CH_3_), CH_2_: 22.86, 25.51, 29.74, 30.21, 32.21, 34.02, 40.46; 53.45 (CH), 65.56 (CH_2_), 69.29 (CH), 70.65 (CH), 71.04 (CH), 71.97 (CH), 72.20 (CH), 72.39 (CH), 102.48 (CH); LRMS for C_23_H_48_N_2_O_7_: MW = 464.6, M (calcd.) = 464.4 (*m/z*), ESI+Q−TOF: M = 464.4 (*m/z*), [M+H]^+^ = 465.4 (13.4%), 466.4 (4.2%), approximately equivalent to the calculated isotopic ratio (100%:25.1%).

*2-Amino-1-*O*-(6-amino-α-D-galactopyranosyl)-D-ribo-octadecan-3,4-diol* (**2**): To a mixture of starting material **15α** and MeOH (8 mL) was added NaOMe (3 mg, 0.05 mmol, 0.5 eq.). The stirring was allowed for 1 h. TLC (EtOAc/*n*-hexane = 1:4) indicated the consumption of the starting material **15α** (R*_f_* = 0.90) and the formation of the product (R*_f_* = 0.58). After adding the cationic exchange resin (H+), the pH was adjusted to neutral. The mixture was filtered through a Celite pad. The filtrate was concentrated and the residue obtained was further purified using column chromatography (MeOH/CHCl_3_ 1:9) to afford white solid of *2-azido-1-O-(6-azido-2,3,4-tri-O-benzyl-α-D-galactopyranosyl)-D-ribo-octadecan-3,4-diol* in 90% yield (57 mg). ^1^H-NMR (CD_3_OD): δ 0.88 (t, 3H, *J* = 7.0 Hz, H_aliphatic_), 1.26–1.36 (m, 24H, H_aliphatic_), 1.60–1.80 (m, 2H, H_aliphatic_), 3.14 (dd, 1H,*J*_6a',6b'_ = 12.5 Hz, *J*_6a',5'_ = 4.5 Hz, H_6a'_), 3.48 (dd, *J*_6b',6a'_= 12.5 Hz, *J*_6b',5'_= 8.5 Hz, 1H, H_6b'_), 3.52–3.57 (m, 1H), 3.59 (dd, *J* = 7.0, 4.5 Hz, 1H), 3.70–3.73 (m, 1H), 3.75 (dd, *J* = 10.5, 6.5 Hz, 1H), 3.94 (bs, 1H), 3.96 (dd, *J* = 10.0, 2.5 Hz, 1H), 4.00 (dd, *J* = 10.0, 3.5 Hz, 1H), 4.10 (dd, *J* = 10.0, 2.5 Hz, 1H), 4.55 (d, *J* = 11.5 Hz,1H, CH_2_Ph), 4.73–4.77 (m, 4H, CH_2_Ph), 4.90 (d, *J* =11.0 Hz, 1H, CH_2_Ph), 4.94 (d, 1H, *J_1,2_* = 3.5 Hz, H_1_), 7.26–7.38 (m, 15H, ArH); ^13^C-NMR (CD_3_OD): δ 14.46 (CH_3_), CH_2_: 23.74, 26.74, 30.48, 30.76, 30.79, 33.07, 34.14, 52.61; 63.68 (CH), 68.98 (CH_2_), 71.66 (CH), 73.01 (CH), 74.18 (CH_2_, CH_2_Ph), 74.28 (CH_2_, CH_2_Ph), 75.99 (CH_2_, CH_2_Ph), 76.46 (CH), 76.96 (CH), 77.30 (CH), 79.85(CH), 99.68( CH), arom. CH: 128.68, 128.79, 128.89, 129.17, 129.33, 129.36, 129.39, 129.42; arom. quaternary C: 139.70, 139.80, 139.96; LRMS for C_45_H_64_N_6_O_7_: M (calcd.) = 800.5 (*m/z*), ESI+Q−TOF: M = 800.3 (*m/z*), [M+Na]^+^ = 823.3. The similar procedure as described for compound **17 ** was employed. The benzoyl-group-removed compound (57 mg, 0.07 mmol), a cosolvent of MeOH (8 mL) and CHCl_3_ (2 mL), glacial AcOH (30 μL) and Pd(OH)_2_ (114 mg) were employed. TLC (NH_3_/MeOH/CHCl_3_ = 0.2/1/1) indicated the formation of the product (R*_f_* = 0.13) and the consumption of the starting material (R*_f_* = 0.95). After 30 h, the mixture was filtered through a Celite pad and washed with MeOH to obtain the filtrate. After concentration under reduced pressure, a white solid of product **2** was obtained in 90% yield (30 mg). LRMS for C_24_H_50_N_2_O_7_: M (calcd.) = 478.4 (*m/z*), ESI+Q-TOF: M = 478.36 (*m/z*), [M+H]^+^ = 479.3 (94.0%), 480.3 (28.9%), 481.3 (5.8%), approximately equivalent to the calculated isotopic ratio (100%:26.8%:4.9%).

***N****-((2S,3S,4R)-2-Amino-3,4-dihydroxyheptadecyl)-4-butylbenzamide* (**18**): Compound **1** (15 mg, 0.05 mmol), 4-butylbenzoic acid (1 eq.) and HBTU (1.2 eq.) were used, respectively. Purification used column chromatography with eluents of MeOH/CHCl_3_ 1:19→1:12 to afford product mixtures, which were observed to be pure in TLC. Further purification using HPLC as described above but with MeOH/CHCl_3_ 1:13 as eluent was used to collect the fraction under the area between 8.5 and 10.5 min. Product **18** was obtained in 35% yield (8 mg). Reinjection of the concentrated fraction into HPLC showed two peaks in the chromatogram. These were suspected to be two conformers due to rotation. Miscellaneuous small unidentified peaks in ^1^H-NMR were impurities, which were also observable in the HPLC chromatogram. The impurities were suspected to be the unremoved diisopropylethylamine, which was confirmed from the spectrum of ESI-MS: [M+H]^+^ = 130.2 (28%), 131.2 (3%); ^1^H-NMR (C_6_D_6_): δ 0.90–0.96 (m, 6H, H_aliphatic_), 1.25–1.50 (m, 22H, H_aliphatic_), 1.62–1.80 (m, 4H, H_aliphatic_), 2.02–2.12 (m, 2H), 2.44–2.47 (m, 2H), 3.99–4.24 (m, 5H), 7.13–7.15 (m, 2H, ArH), 8.04–8.06 (m, 2H, ArH), 8.29 (bs, 1H, amide); ^13^C-NMR (C_6_D_6_): δ 8.13, 14.10, 14.37, 22.78, 23.14, 26.36, 29.92, 30.27, 30.38, 30.45, 30.49, 30.53, 32.39, 33.45, 35.83, 46.62, 54.92, 66.20, 72.60, 74.10, 127.47, 129.01, 130.67, 147.83, 170.38; LRMS for C_28_H_50_N_2_O_3_: M (calcd.) = 462.4 (*m/z*), ESI+Q-TOF: M = 462.4 (*m/z*), [M+H]^+^ = 463.4 (100%), 464.4 (29%), 465.4 (5%), [M+Na]^+^ = 485.3 (8%), 486.4 (2%), approximately equivalent to the calculated isotopic ratio (100%:31%:5.3%).

*4-Butyl-*N*-(((2*R*,3*R*,4*S*,5*R*,6*S*)-6-(((2*S*,3*S*,4*R*)-2-(4-butylbenzamido)-3,4-dihydroxyoctadecyl)oxy)-3,4,5-trihydroxytetrahydro-2H-pyran-2-yl)methyl)benzamide* (**19**): To a mixture of 4-butylbenzoic acid (23 mg, 0.13 mmol, 2.1 equiv), HBTU (57 mg, 0.15 mmol, 2.4 equiv) and DMF (6 mL) was added diisopropylethylamine (14 μL, 0.08 mmol, 1.3 eq.) under N_2_. After stirring for 10 min, TLC (EtOAc/*n*-hexane = 1:3) indicated the formation of the ester intermediate (R*_f_* = 0.73) and consumption of the starting 4-butylbenzoic acid (R_f_ = 0.12). To this mixture was added the solution of compound **2** (30 mg, 0.06 mmol) in DMF (4 mL). After stirring for 30 h, TLC (NH_3_/MeOH/CHCl_3_ = 0.2:1:1) indicated the formation of the product **19** (R*_f_* = 0.89) and consumption of the starting compound **2** (R*_f_* = 0.14). The mixture was concentrated under reduced pressure. The residue obtained was purified using column chromatography (EtOAc/n-hexane = 1:4) to afford **19** as a white solid in 60% yield (31 mg). The sample was further purified using HPLC (0.9 cm × 20 cm, Si-100) with MeOH/CHCl_3_ = 1:29 as eluent at a flow rate of 3 mL/min to a afford white solid (5 mg); t_R_ = 19.2 min; t_R_ = 11.9 min (aromatic impurities). Anal. C_46_H_74_N_2_O_9_, M (calcd.) = 798.5 (m/z), ESI+Q−TOF: M = 798.6 (m/z), [M+H]^+^ = 799.6 (19.04%), 800.6 (10.99%), [M+Na]^+^= 821.6 (100%), 822.6 (50.09%), 823.6 (11.33%), equivalent to the calculated isotopic ratio 100:50.8:12.7; HRMS (ESI) M (calcd.) = 798.53943 (m/z), M (found) = 798.53975 (m/z); ^1^H-NMR (CD_3_OD): δ 0.87–0.94 (m, 9H, H_aliphatic_), 1.21–1.40 (m, 28H, H_aliphatic_), 1.60–1.70 (m, 5H, H_aliphatic_), 2.61–2.67 (m, 4H), 3.44–3.48 (m, 1 H), 3.56–3.60 (m, 1 H), 3.67–3.82 (m, 6H), 3.93–4.00 (m, 2H), 4.40–4.44 (dd, 1H, J = 10.5, J = 5.0 Hz), 4.93–4.94 (d, 1H, J = 3.5 Hz, H_1_), 7.20–7.25 (m, 4H, ArH), 7.67–7.71 (m, 4 H, ArH); ^13^C-NMR (CD_3_OD): δ 14.18 (CH_3_), 14.36 (CH_3_), 23.29 (CH_2_), 23.32 (CH_2_), 23. 67 (CH_2_), 26.77 (CH_2_), 30.41(CH_2_), 30.71 (CH_2_), 30.75 (CH_2_), 33.03 (CH_2_), 33.47 (CH_2_), 34.54 (CH_2_), 36.46 (CH_2_), 41. 69 (CH_2_), 52.60 (CH_2_), 67.96 (CH_2_), 70.18 (CH), 70.52 (CH), 71.29 (CH), 71.46 (CH), 73.04 (CH), 75.85 (CH_3_), 101.13 (CH), arom: 128.38, 128.46, 129.57, 132.93, 133.19, 148.34, 148.27; 169.93 (amide), 170.76 (amide).

### 3.3. Preparation of Cell Lines and MTT Assay

#### 3.3.1. Cell Culture

Adherent normal human fibroblast and U87 cells were maintained at 37 °C in a humidified CO_2_-controlled atmosphere in Minimum Essential Medium (MEM) (Sigma-Aldrich) supplemented with 10% heat-inactivated fetal bovine serum (FBS) (Biological Industries). In addition, adherent A549 and C26 cells were maintained at 37 °C in a humidified CO_2_-controlled atmosphere in RPMI 1640 (Sigma-Aldrich) supplemented with 10% heat-inactivated fetal bovine serum (FBS) (Biological Industries).

#### 3.3.2. MTT Assay of Amide-Bond Formation Products

##### 3.3.2.1. Cell Plating

Briefly, 3,000 cells per well were plated in 96-well microtiter plates with 100 μL MEM/10%FBS and incubated at 37 °C in a humidified CO_2_-controlled atmosphere for 1 day.

##### 3.3.2.2. Construction of Amide Bonding Libraries and Their Cytotoxicity Screening

**Figure 4:**
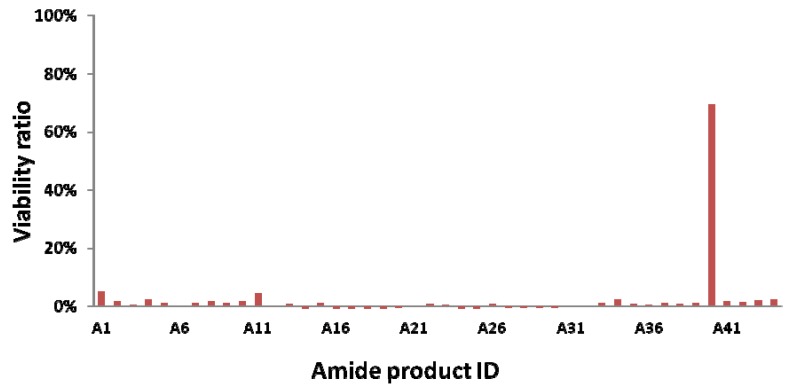
Analog **18** (A11) showed the less cytotoxicites against normal human fibroblasts (50% in U87 cells). A40 was obtained from (2*R*,3*R*)-2,3-bis(4-methylbenzoyloxy)succinic acid. Purification of the product mixtures of A40 with HPLC generated a number of unidentified peaks in chromatogram.

We used 44 carboxylic acids (Figure 2) to construct amide bonding libraries. Every carboxylic acid (1 eq., dissolved in 25 μL DMSO) was activated by HBTU (1.1 eq., 4.1 mg, dissolved in 23 μL DMSO) and DIEA (1.2 eq., 0.012 mmol, 2 μL). The amide bonding reaction was carried out by coupling a portion of crude active ester (10 μL, 0.2 M, dissolved in DMSO) with amine (10 μL, 0.2 M, dissolved in DMSO). After completion of amide bond formation, a portion of the crude product (0.1 M, dissolved in 4 μL DMSO) was diluted by de-ionized sterilized water (396 μL) and filtrated with 0.2 μm filter. The filtrate (1 mM crude product in 10 μL water containing 1% (v/v) DMSO) was diluted with 100 µL culture medium in the previous cell-plated microtiter plates so that the concentration of DMSO was less than 0.1% (v/v), and the crude product was less than 100 μM These microtiter plates was further incubated at 37 °C in a humidified CO_2_-controlled atmosphere for 2 days. 3-(4,5-dimethylthiazol-2-yl)-2,5-diphenyltetrazolium bromide (0.5 mg dissolved in 1 mL PBS buffer) was added to previous microtiter plates and incubated for 4 h. After removing the culture medium from microtiter plates and dissolving insoluble formazan with 100 μL DMSO, cytotoxicity screening data was obtained by dectecting the absorbance of 570 nm with microtiter plate reader (Plate CHAMELEON^TM^). The MTT assay results are shown below (Figure 4).

### 3.4. Invariant Nature Killer Cell Quantification

The iNKT was obtained from peripheral blood monocytes (PBMC) of healthy donors after gradient-separated at 400 g, 30 min with Ficoll-Hypaque^TM^ plus (GE Healthcare, CA, USA). The cells were cultured and enriched in RPMI with L-glutamin (Gibco, NY, USA) with supplement of 10% fetal calf serum and 1% penicillin-streptomycin. α-Galactosylceramide (α-GalCer, Kirin, Gunma, Japan) was added to the medium at defined concentration every 3 days. The iNKT population was either identified or sorted (fluorescence-activated cell sorted (FACS), magnetic cell seperation) with antibodies against Vα24^+^/Vβ11^+^. The antibodies for staining T-cell receptors (TCR, Vα24^+^/Vβ11^+^) were purchased from Beckman Coulter (city?CA, USA), BD bioscience (NJ, USA) and Miltenyi-Biotec (CA, USA). Briefly, after 7–10 days of incubation, the cultured cells were analyzed with flow cytometry.

## 4. Conclusions

The 6-aminogalactosylsphingosine analog has been prepared as core compound for the construction of libraries. A mini library comprising 40+ compounds have been generated through parallel solution phase synthesis via amide bond formation. A preliminary test of the bioactivity including the use of cytotoxicity assays and flow cytometry assays for NKT cell proliferation have been performed accordingly. A subtle inducement of the subpopulation of V 24+/V-β11+ cells by compound **19** needs further study to clarify its role.

## Supplementary Materials

Supplementary materials can be accessed at: http://www.mdpi.com/1420-3049/17/3/3058/s1.
