# Supplementary material for: Synthesis of Amino Core Compounds of Galactosyl Phytosyl Ceramide Analogs for Developing iNKT-Cell Inducers
Source: Molecules. 2012 Mar 12;17(3):3058–81. doi: 10.3390/molecules17033058 (PMC6268237; doi:10.3390/molecules17033058)

<u>Supplementary Information</u>

^{1}H- and ^{13}C-NMR, ESI-MS spectra and HPLC chromatogram

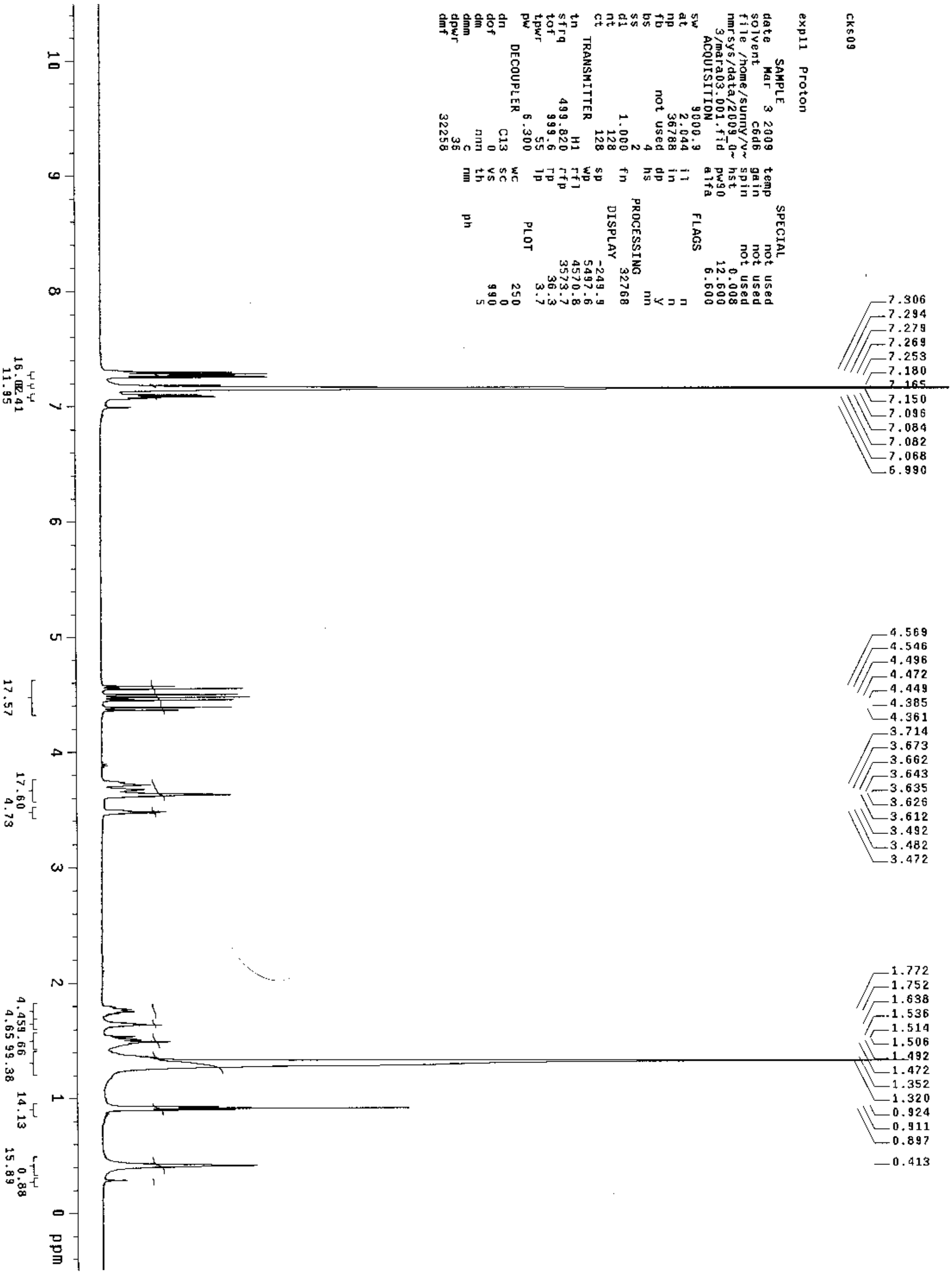

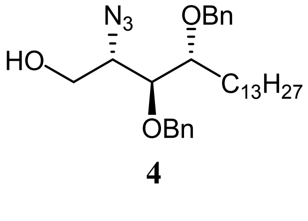

4

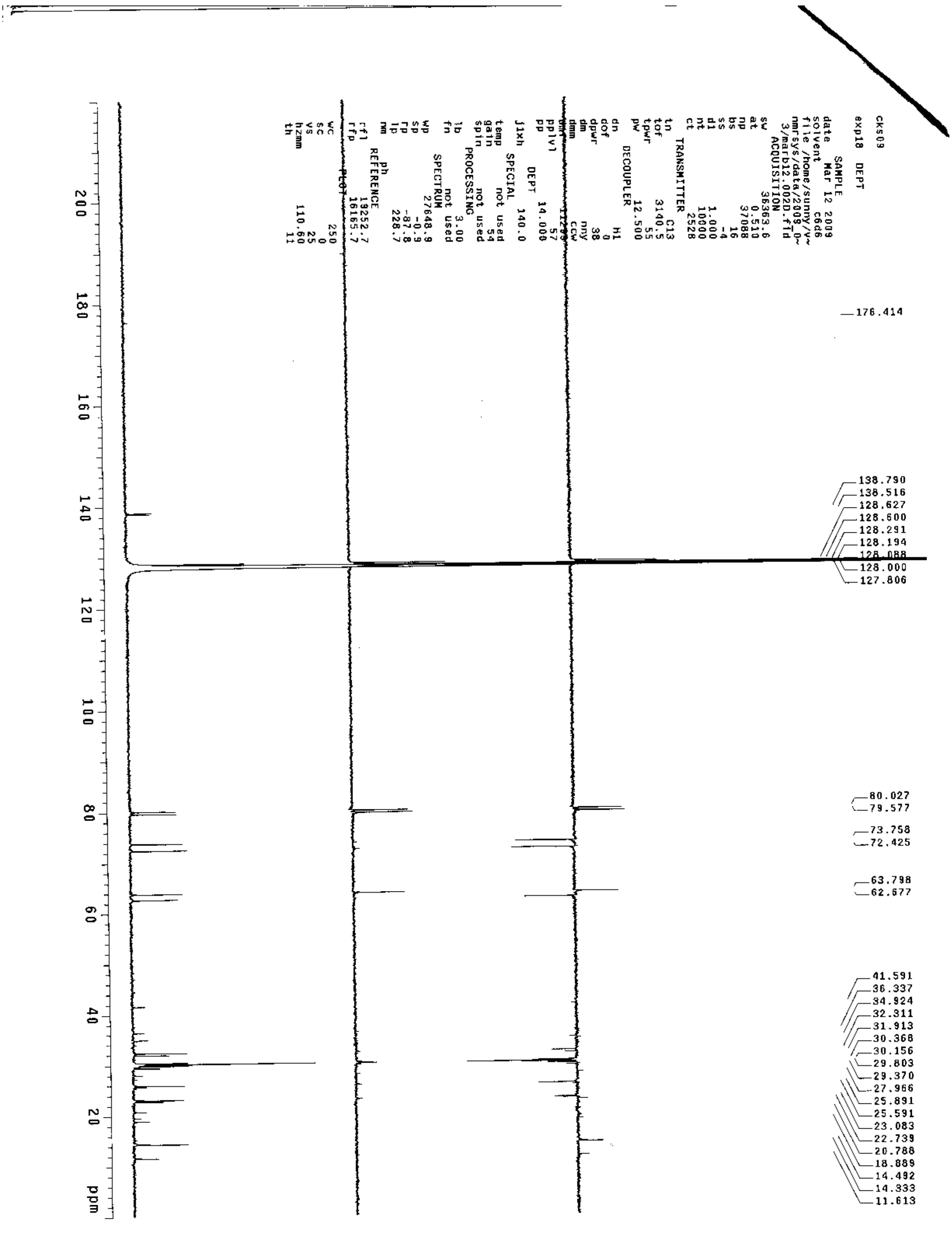
176.414
138.790
138.516
128.627
128.600
128.291
128.194
128.088
128.000
127.806
80.027
79.577
73.758
72.425
63.798
62.677
41.591
36.337
34.924
32.311
31.913
30.368
30.156
29.803
29.370
27.966
25.891
25.591
23.083
22.739
20.788
18.889
14.492
14.333
11.613
ppm

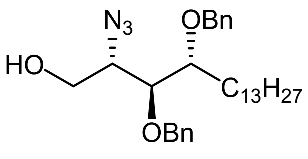
N3
OBn
HO
C13H27
OBn

4

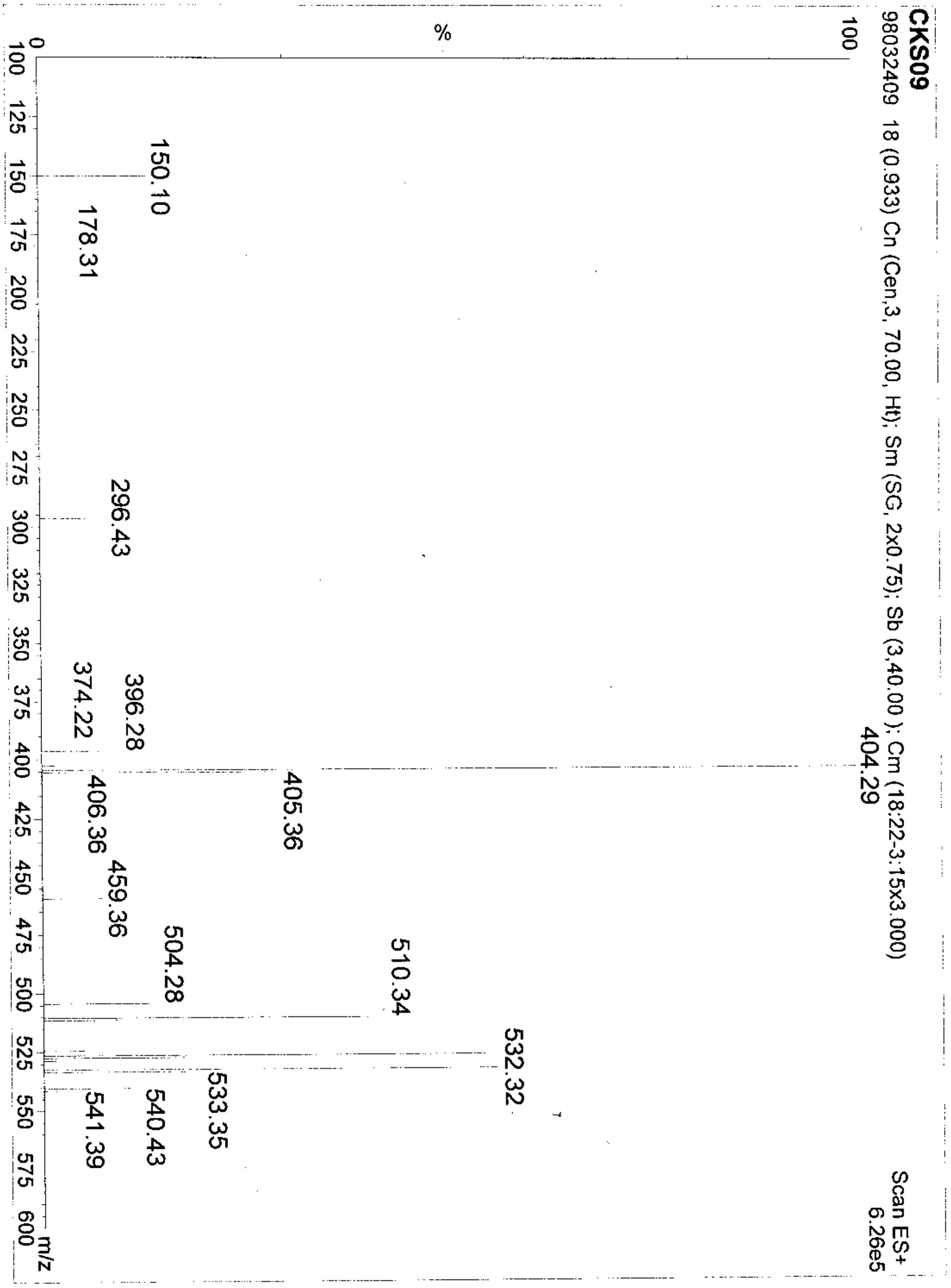
CKS09
98032409 18 (0.933) Cn (Cen,3, 70.00, Ht); Sm (SG, 2x0.75); Sb (3,40.00); Cm (18:22-3:15x3.000)
Scan ES+
6.26e5
100
%
0
150.10
178.31
296.43
374.22
396.28
404.29
405.36
406.36
459.36
504.28
510.34
532.32
533.35
540.43
541.39
100 125 150 175 200 225 250 275 300 325 350 375 400 425 450 475 500 525 550 575 600
m/z

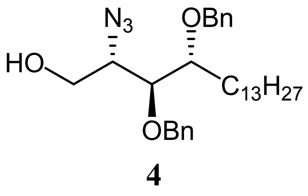
N3
OBn
HO
C13H27
OBn

4

國立交通大學應用化學系

元素分析儀 Heraeus CHN-O Rapid 服務報告書

使用者姓名：張凱翔　　　　中心編號：980357

服務單位：清大原科 俞鐘山實驗室　樣品名稱或代號：CKS09

收件日期：98 年 3 月 24 日 完成日期：98 年 3 月 26 日

分析結果：

實驗值：	N%	C%	H%
1.	8.10	72.85	8.49
2.	8.21	72.74	9.09
3.			
4.			
推測值：	8.24	73.05	9.29

本日所使用之 Standard：A

(A)Acetanilide　(B)Atropin　(C)N-Anilin

	N%	C%	H%
理論值：	10.36	71.09	6.71
測出值：	10.42	70.86	6.55

備註：

費用核算：NCH：800 元

報告日期：98 年 3 月 27 日

儀器負責人簽章：謝有容　　技術員簽章：技士李蘊明

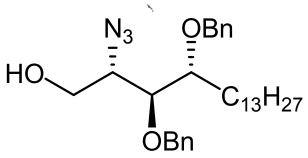

4

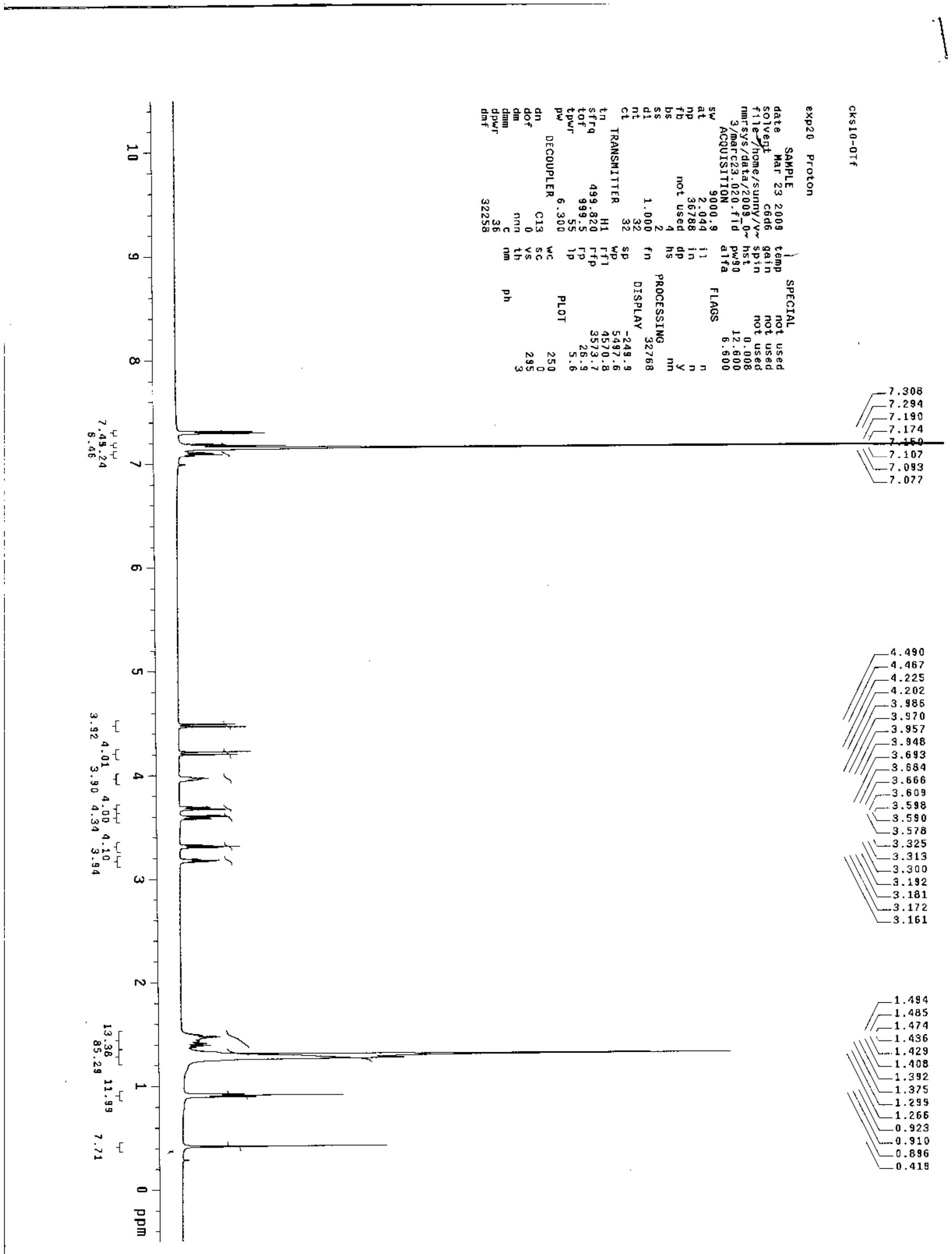

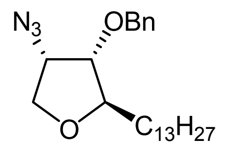

6

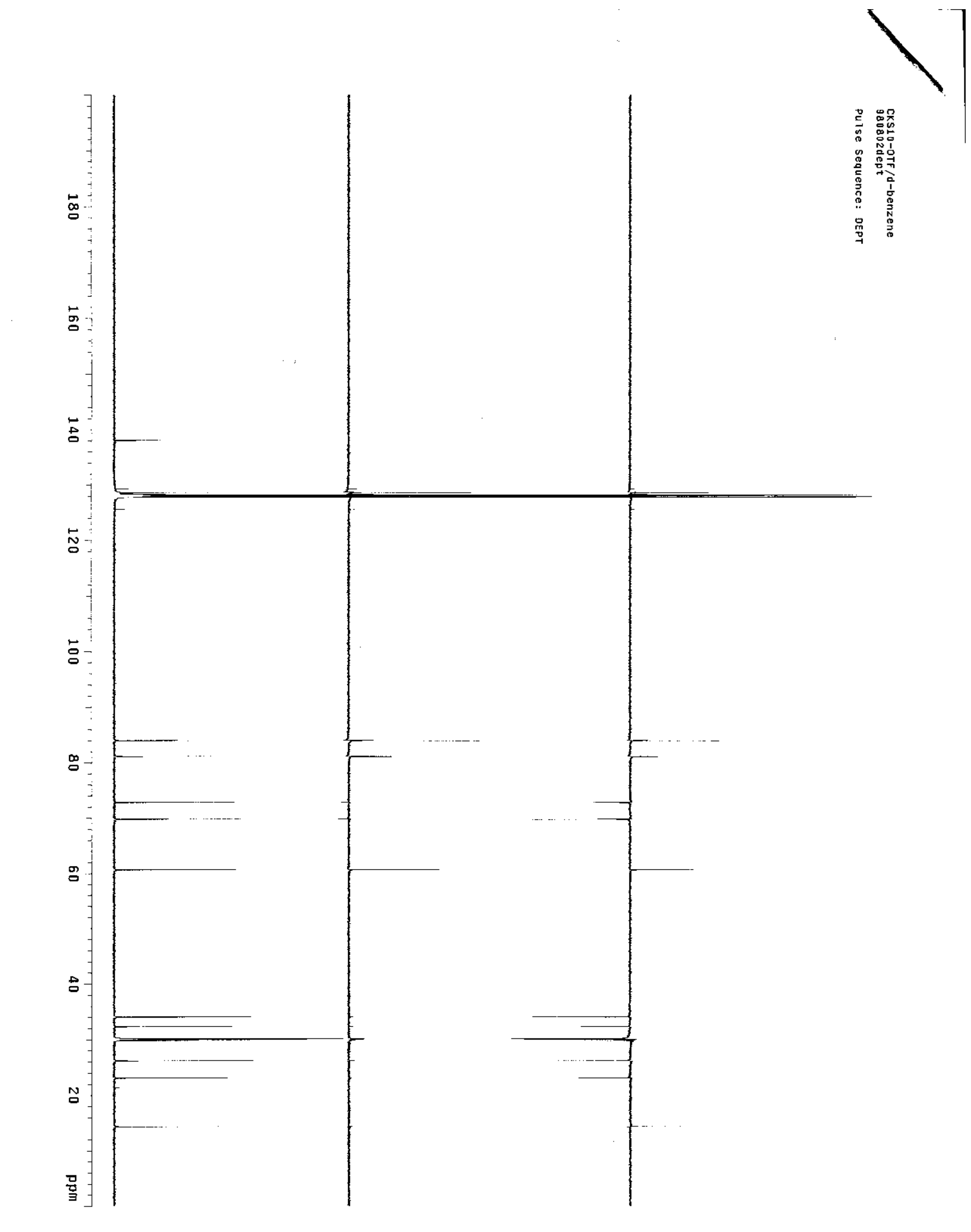

CKS10-OTf/d-benzene
980802dept
Pulse Sequence: DEPT
180
160
140
120
100
80
60
40
20
ppm

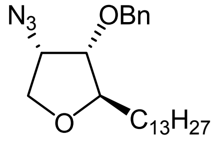

N3
OBn
O
C13H27

6

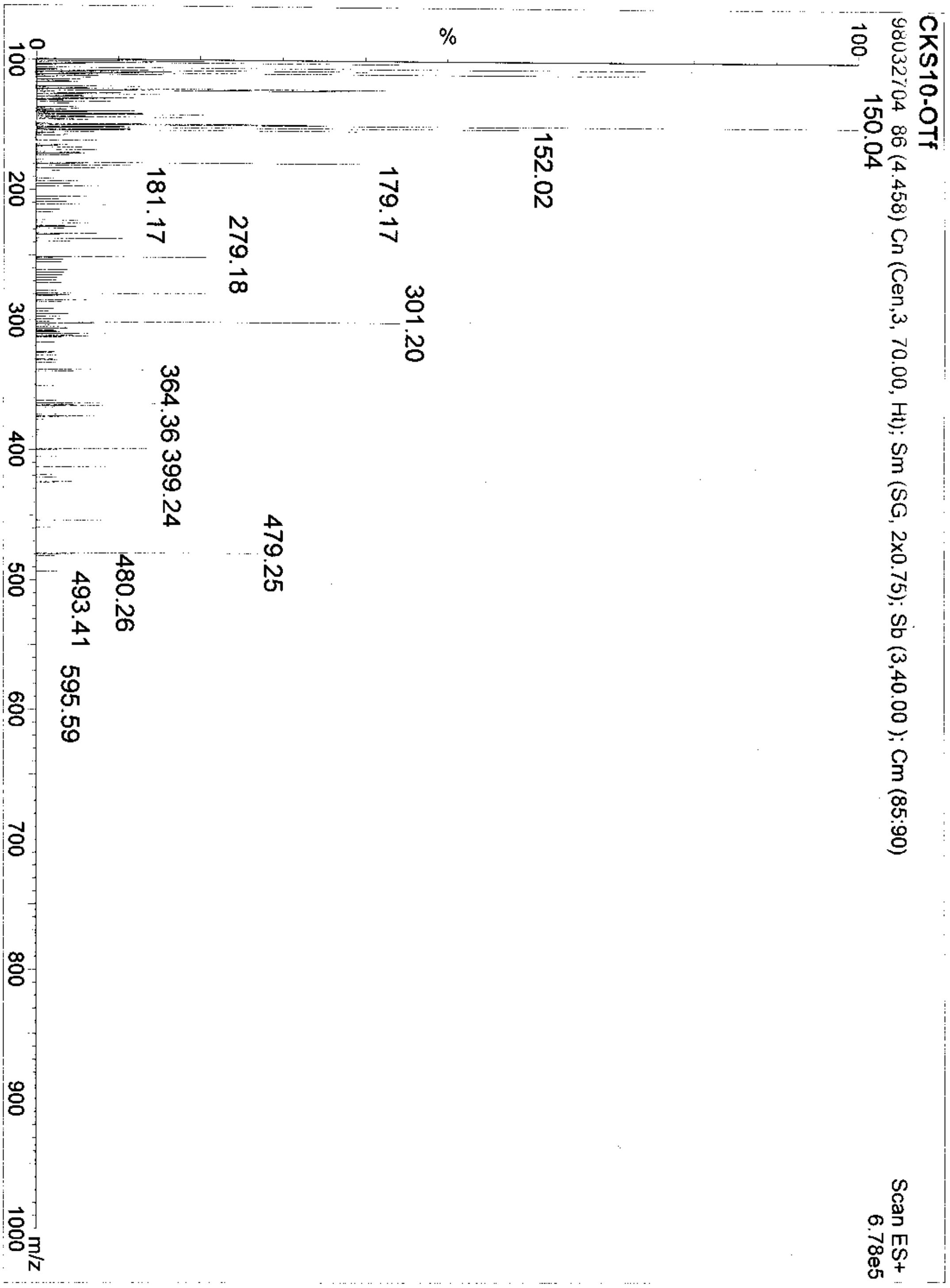
CKS10-OTf
98032704 86 (4.458) Cn (Cen,3, 70.00, Ht); Sm (SG, 2x0.75); Sb (3,40.00); Cm (85:90)
Scan ES+
6.78e5
%
100
0
150.04
152.02
179.17
181.17
279.18
301.20
364.36 399.24
479.25
480.26
493.41
595.59
100
200
300
400
500
600
700
800
900
1000
m/z

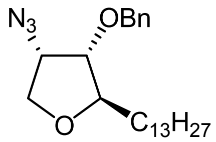
N3
OBn
O
C13H27

6

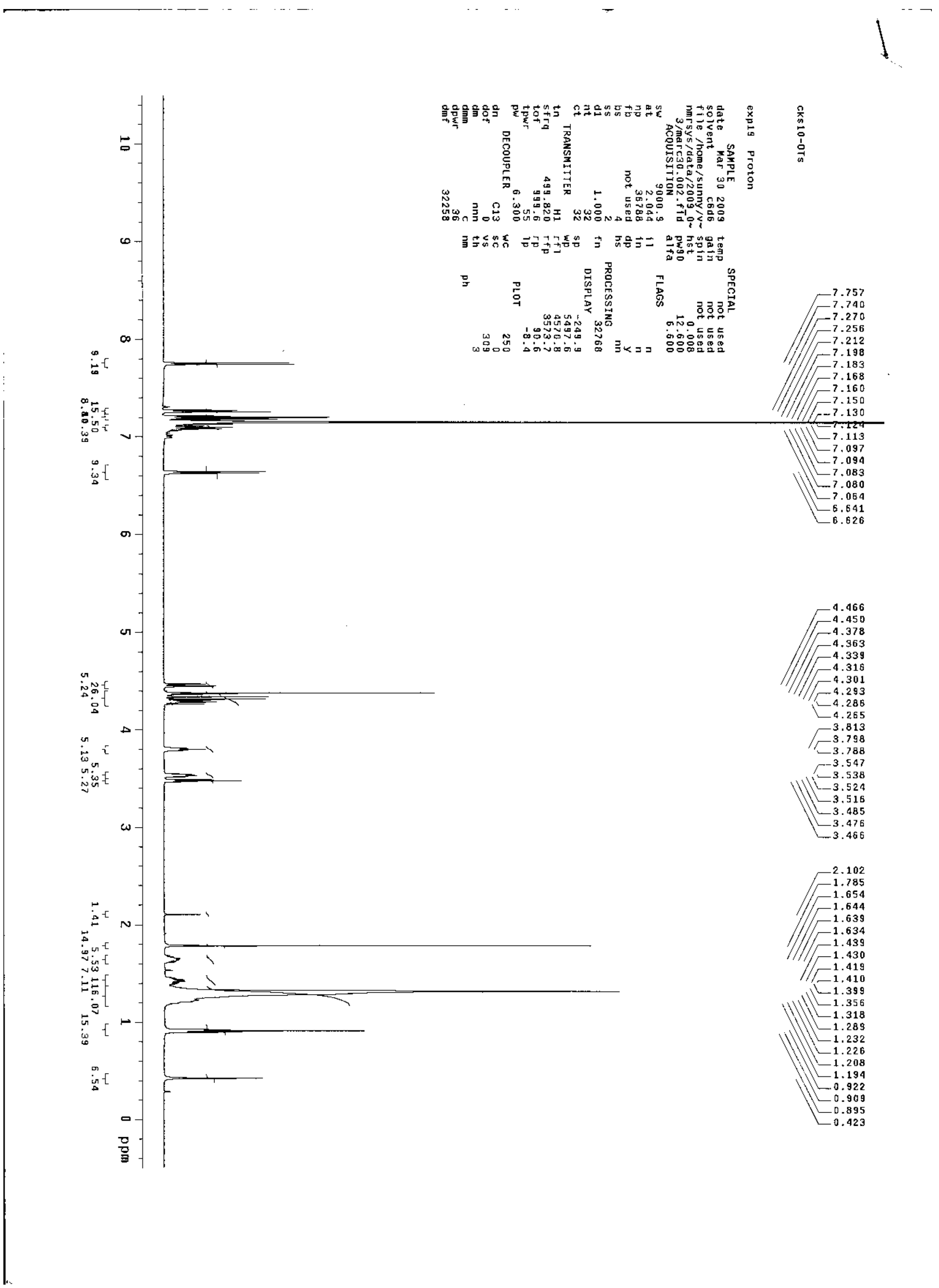

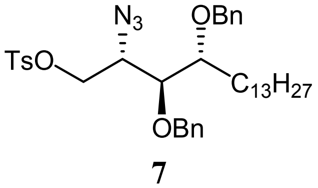

7

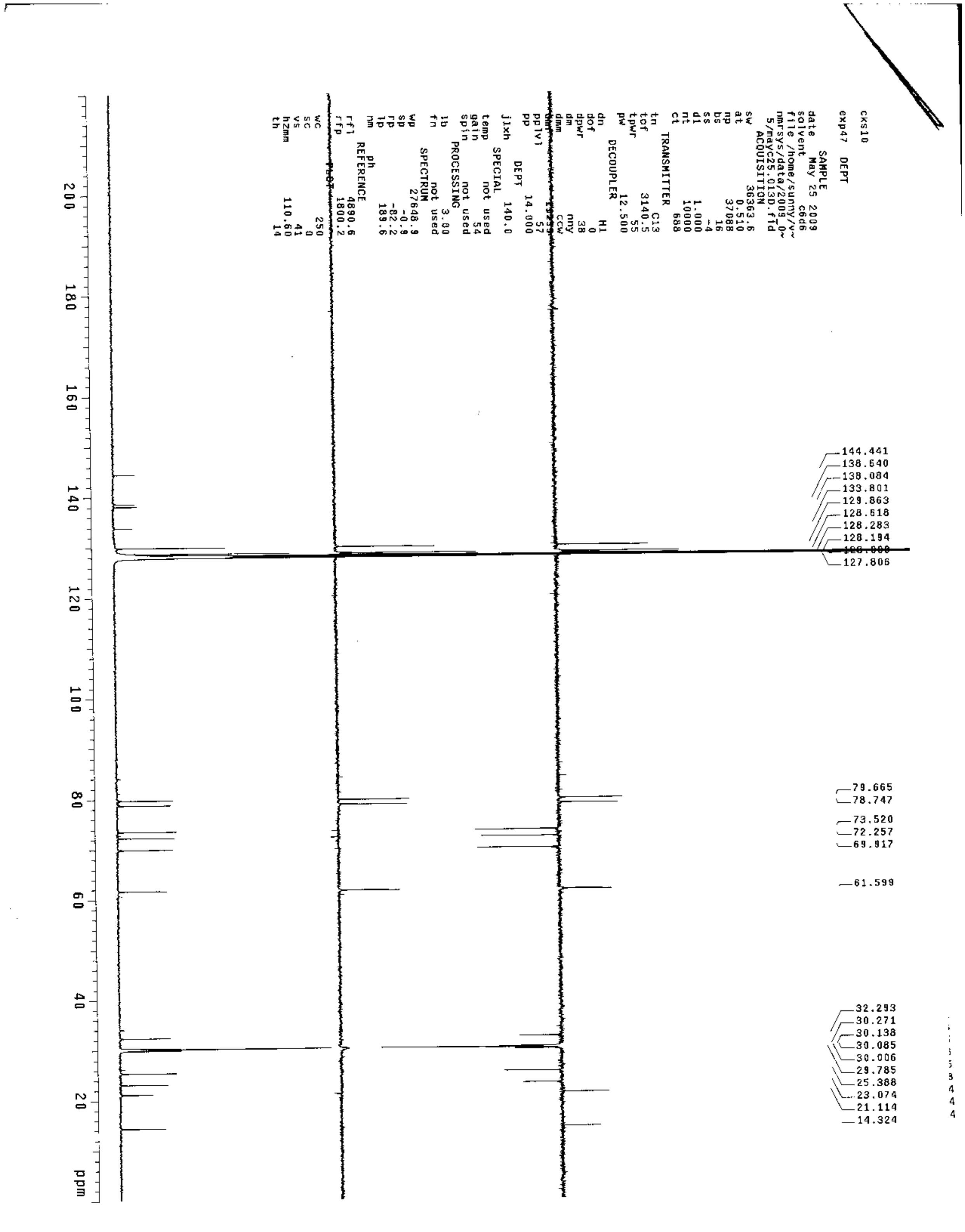
144.441
138.640
138.084
133.801
129.863
128.518
128.283
128.194
[illegible]
127.806
79.665
78.747
73.520
72.257
69.917
61.599
32.293
30.271
30.138
30.085
30.006
29.785
25.388
23.074
21.114
14.324
ppm

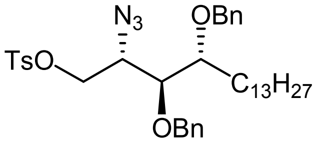
N3
OBn
TsO
C13H27
OBn

7

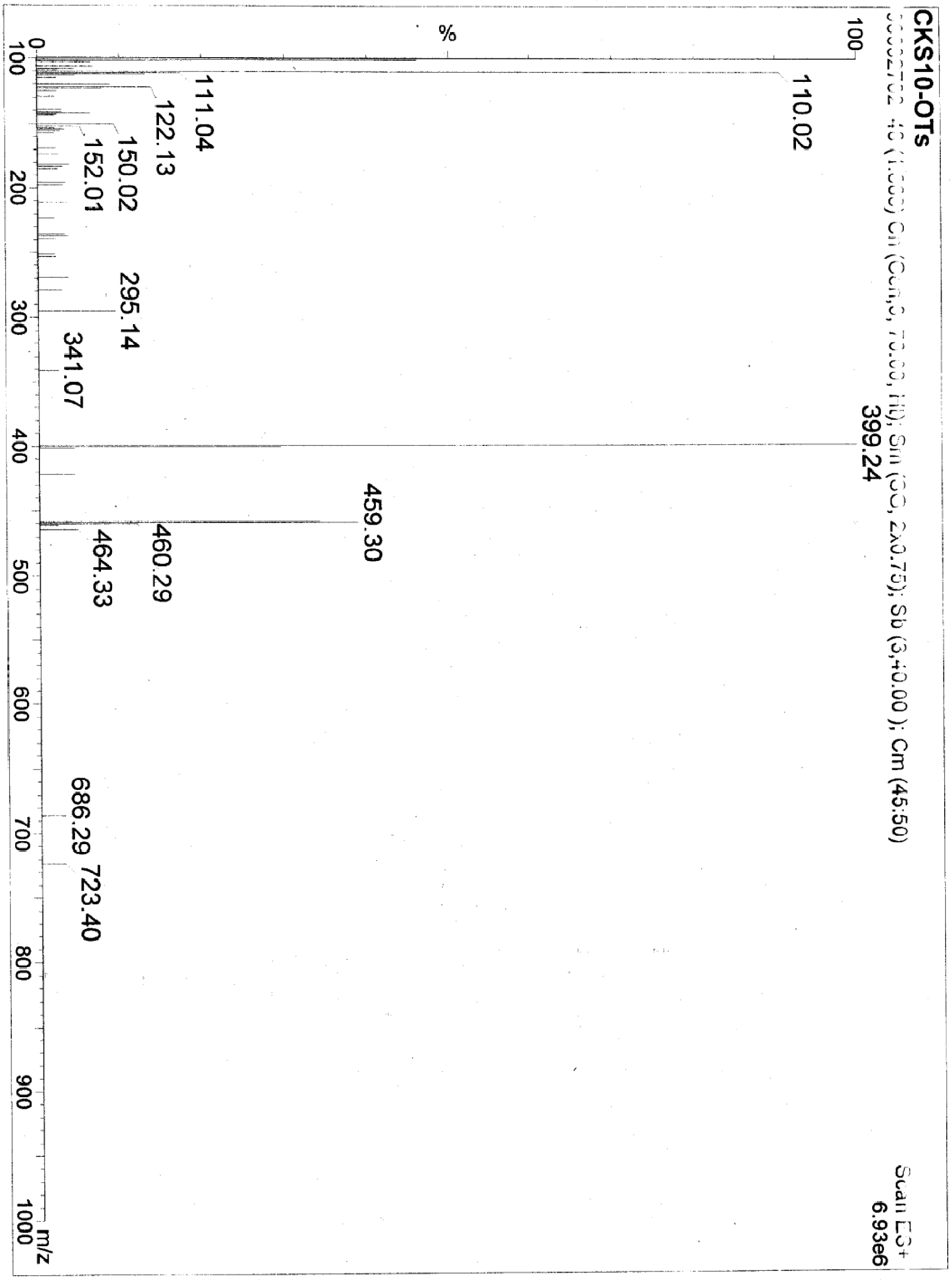
CKS10-OTs
Scan ES+
6.93e6
%
100
0
110.02
111.04
122.13
150.02
152.01
295.14
341.07
399.24
459.30
460.29
464.33
686.29
723.40
100
200
300
400
500
600
700
800
900
1000
m/z

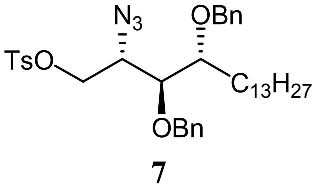
N3
OBn
TsO
C13H27
OBn

7

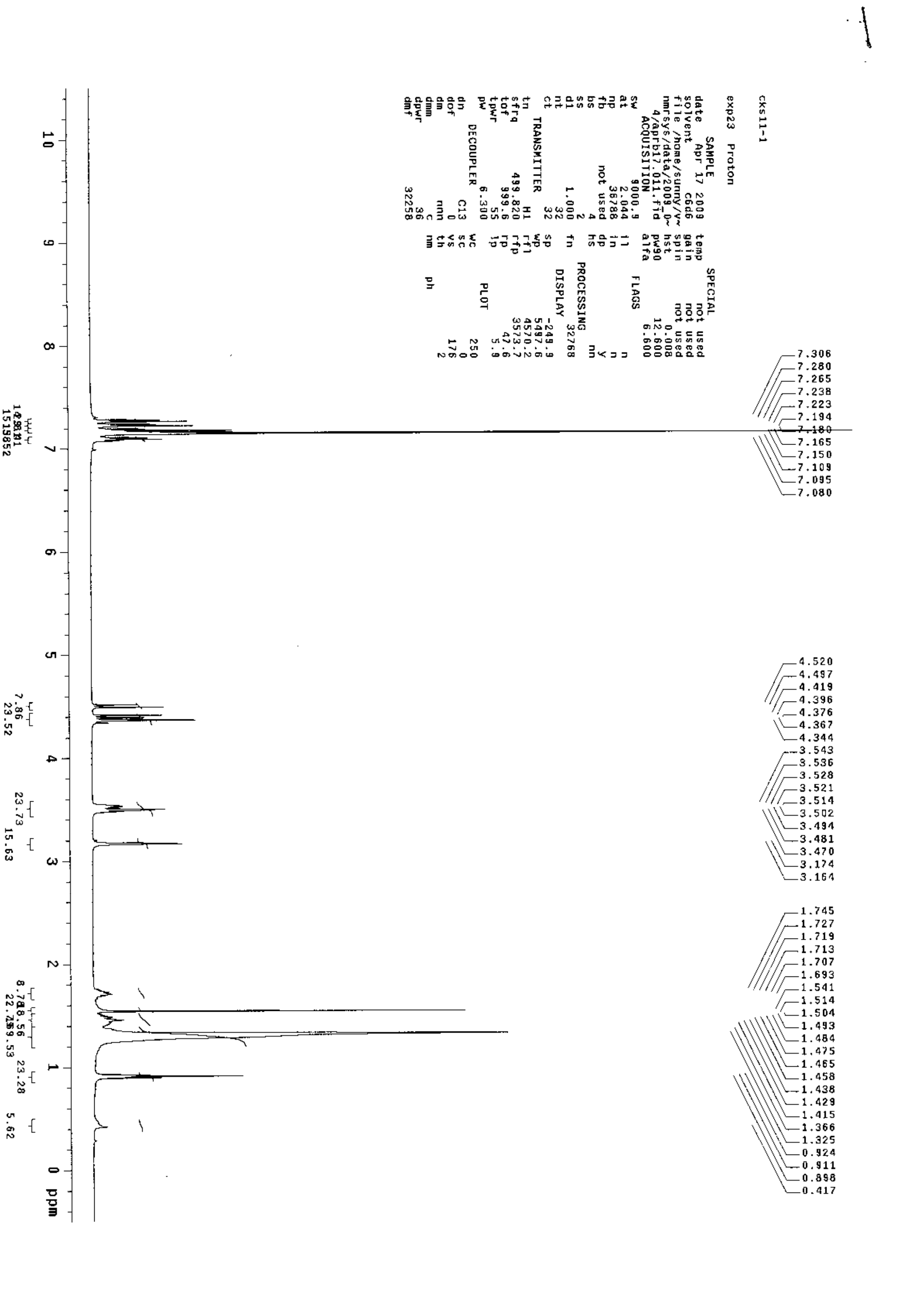

cks11-1
exp23 Proton
SAMPLE
date Apr 17 2009
solvent C6d6
file /home/sunny/v~
nmrsys/data/2009_0~
4/aprb17.011.fid
ACQUISITION
sw 9000.9
at 2.044
np 36788
fb not used
bs 4
ss 2
d1 1.000
nt 32
ct 32
TRANSMITTER
tn H1
sfrq 499.820
tof 999.6
tpwr 55
pw 6.300
DECOUPLER
dn C13
dof 0
dm nnn
dmm c
dpwr 36
dmf 32258
SPECIAL
temp not used
gain not used
spin not used
hst 0.008
pw90 12.600
alfa 6.600
FLAGS
il n
in n
dp y
hs nn
PROCESSING
fn 32768
DISPLAY
sp -249.9
wp 5497.6
rfl 4570.2
rfp 3573.7
rp 47.6
lp 5.9
PLOT
wc 250
sc 0
vs 176
th 2
nm ph
7.306
7.280
7.265
7.238
7.223
7.194
7.180
7.165
7.150
7.109
7.095
7.080
4.520
4.497
4.419
4.396
4.376
4.367
4.344
3.543
3.536
3.528
3.521
3.514
3.502
3.494
3.481
3.470
3.174
3.164
1.745
1.727
1.719
1.713
1.707
1.693
1.541
1.514
1.504
1.493
1.484
1.475
1.465
1.458
1.438
1.429
1.415
1.366
1.325
0.924
0.911
0.898
0.417
7.86
23.52
23.73
15.63
23.28
5.62
10
9
8
7
6
5
4
3
2
1
0
ppm

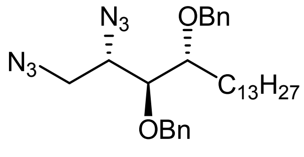

N3
OBn
N3
C13H27
OBn

8

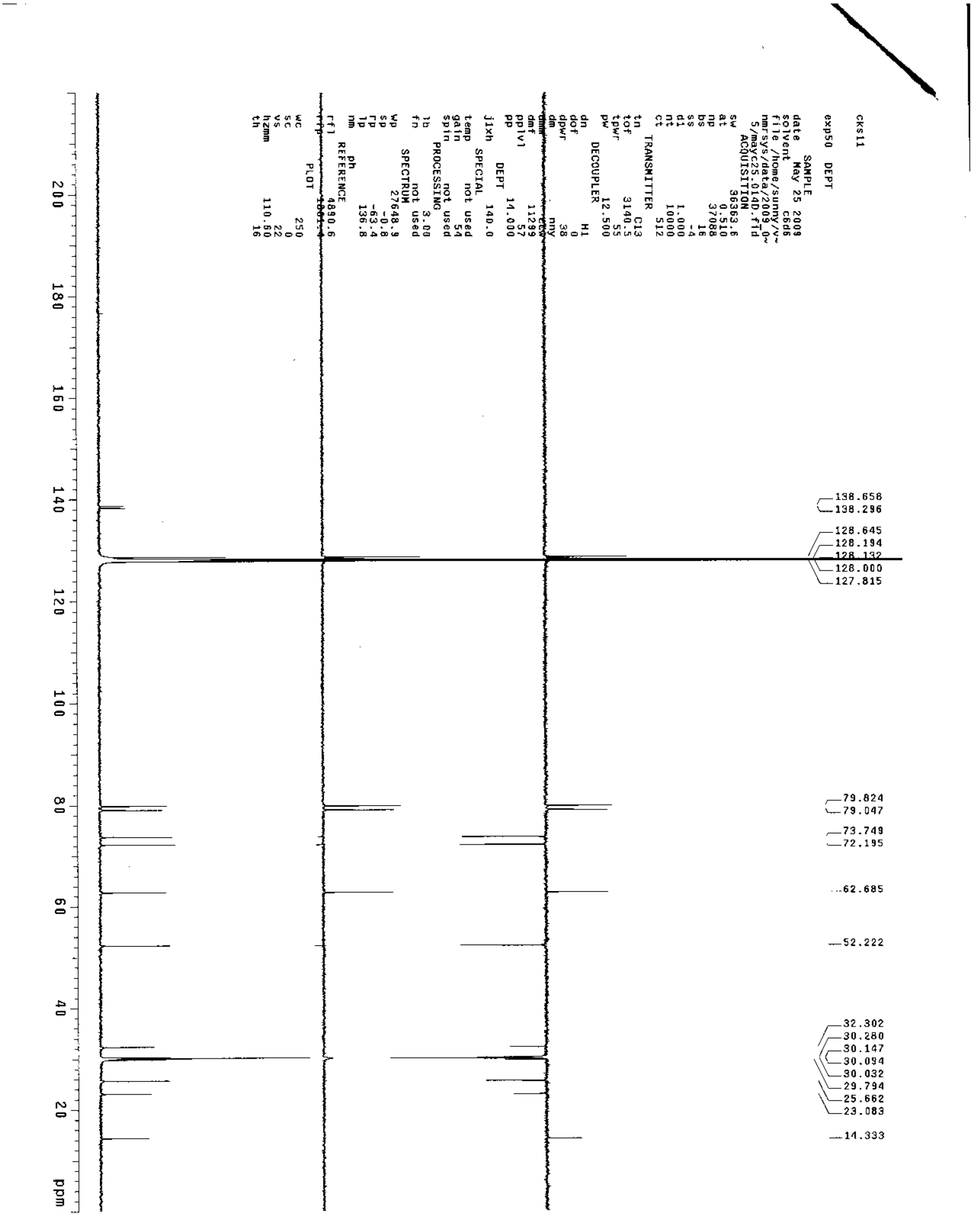
138.658
138.296
128.645
128.194
128.132
128.000
127.815
79.824
79.047
73.749
72.195
62.685
52.222
32.302
30.280
30.147
30.094
30.032
29.794
25.662
23.083
14.333
ppm

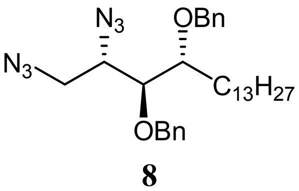
N3
OBn
N3
C13H27
OBn

8

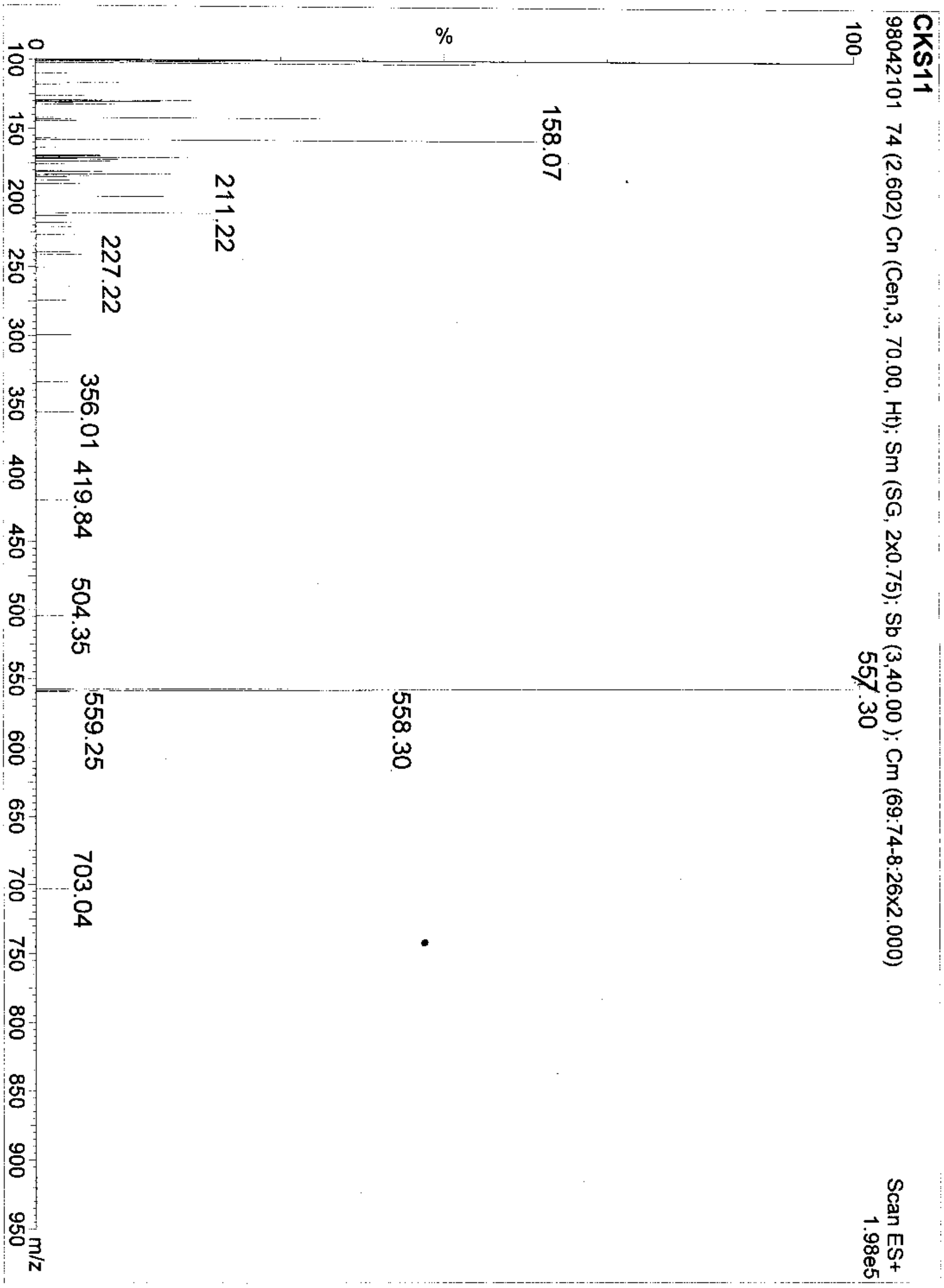
CKS11
9804201 74 (2.602) Cn (Cen,3, 70.00, Ht); Sm (SG, 2x0.75); Sb (3,40.00); Cm (69:74-8:26x2.000)
Scan ES+
1.98e5
100
%
0
557.30
158.07
558.30
211.22
227.22
559.25
356.01
419.84
504.35
703.04
m/z
100
150
200
250
300
350
400
450
500
550
600
650
700
750
800
850
900
950

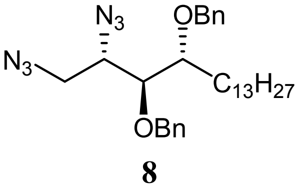
N3
OBn
N3
C13H27
OBn

8

國立交通大學應用化學系

元素分析儀 Heraeus CHN-O Rapid 服務報告書

使用者姓名：張凱翔　　　　　中心編號：980530

服務單位：清大原科 俞鐘山實驗室　樣品名稱或代號：CKS11

收件日期：98 年 5 月 14 日　完成日期：98 年 5 月 18 日

分析結果：

實驗值：	N%	C%	H%
1.	16.03	69.96	8.24
2.	15.83	69.40	8.53
3.			
4.			
推測值：	15.72	69.63	8.67

本日所使用之 Standard：A

(A)Acetanilide　(B)Atropin　(C)N-Anilin

	N%	C%	H%
理論值：	10.36	71.09	6.71
測出值：	10.34	71.01	7.09

備註：

費用核算：NCH：800 元

報告日期：98 年 5 月 19 日

儀器負責人簽章：[illegible]　　技術員簽章：技士李蘊明

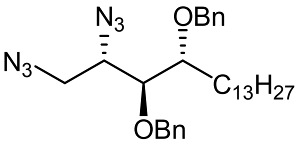

8

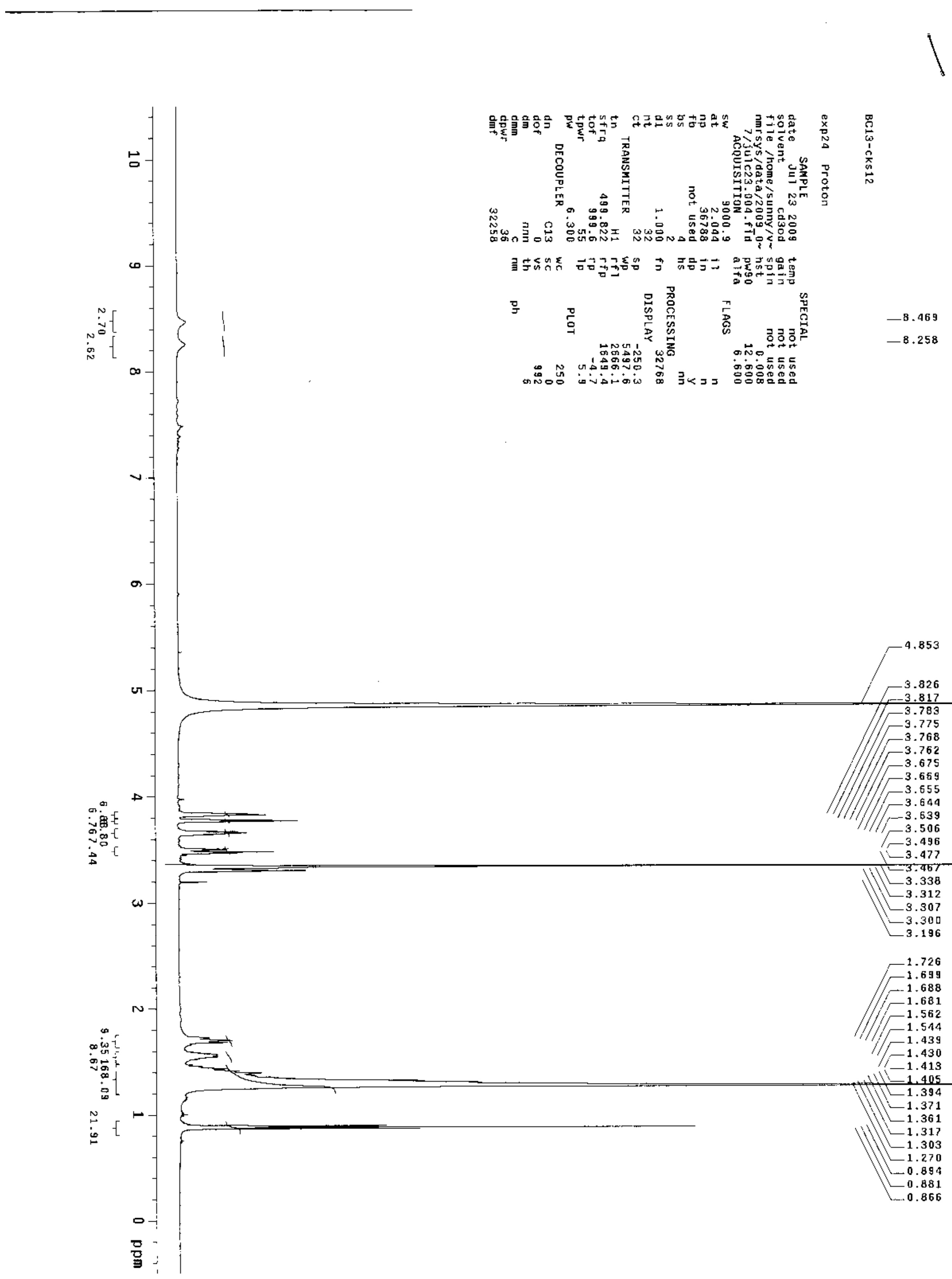
BC13-cks12
exp24 Proton
SAMPLE
date Jul 23 2009
solvent cd3od
file /home/sunny/v~
nmrsys/data/2009_0~
7/Jul23.004.fid
ACQUISITION
sw 9000.9
at 2.044
np 36788
fb not used
bs 4
ss 2
d1 1.000
nt 32
ct 32
TRANSMITTER
tn H1
sfrq 499.822
tof 999.6
tpwr 55
pw 6.300
DECOUPLER
dn C13
dof 0
dm nnn
dmm c
dpwr 36
dmf 32258
SPECIAL
temp not used
gain not used
spin not used
hst 0.008
pw90 12.600
alfa 6.600
FLAGS
il n
in n
dp y
hs nn
PROCESSING
fn 32768
DISPLAY
sp -250.3
wp 5497.6
rfl 2666.1
rfp 1649.4
rp -4.7
lp 5.9
PLOT
wc 250
sc 0
vs 992
th 6
ph
8.469
8.258
4.853
3.826
3.817
3.783
3.775
3.768
3.762
3.675
3.669
3.655
3.644
3.639
3.506
3.496
3.477
3.467
3.338
3.312
3.307
3.300
3.196
1.726
1.699
1.688
1.681
1.562
1.544
1.439
1.430
1.413
1.405
1.394
1.371
1.361
1.317
1.303
1.270
0.894
0.881
0.866
2.70
2.62
8.67
21.91
10
9
8
7
6
5
4
3
2
1
0
ppm

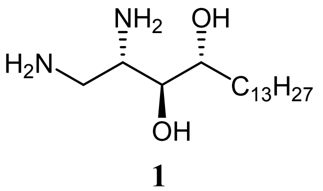
NH2
OH
H2N
C13H27
OH

1

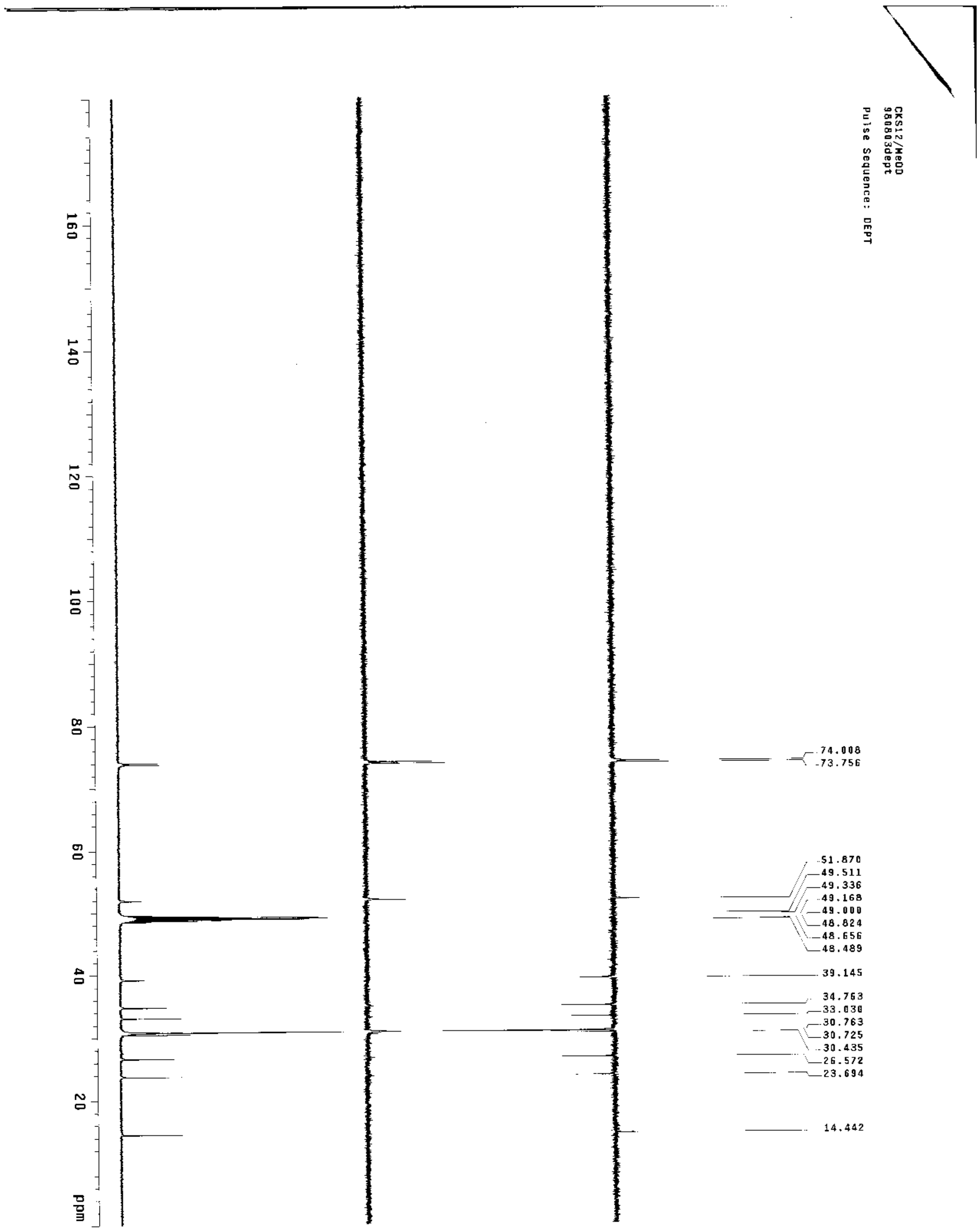
CKS12/MeOD
980803dept
Pulse Sequence: DEPT
74.008
73.756
51.870
49.511
49.336
49.168
49.000
48.824
48.656
48.489
39.145
34.763
33.030
30.763
30.725
30.435
26.572
23.694
14.442
160
140
120
100
80
60
40
20
ppm

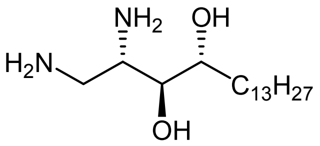
NH2
OH
H2N
C13H27
OH

1

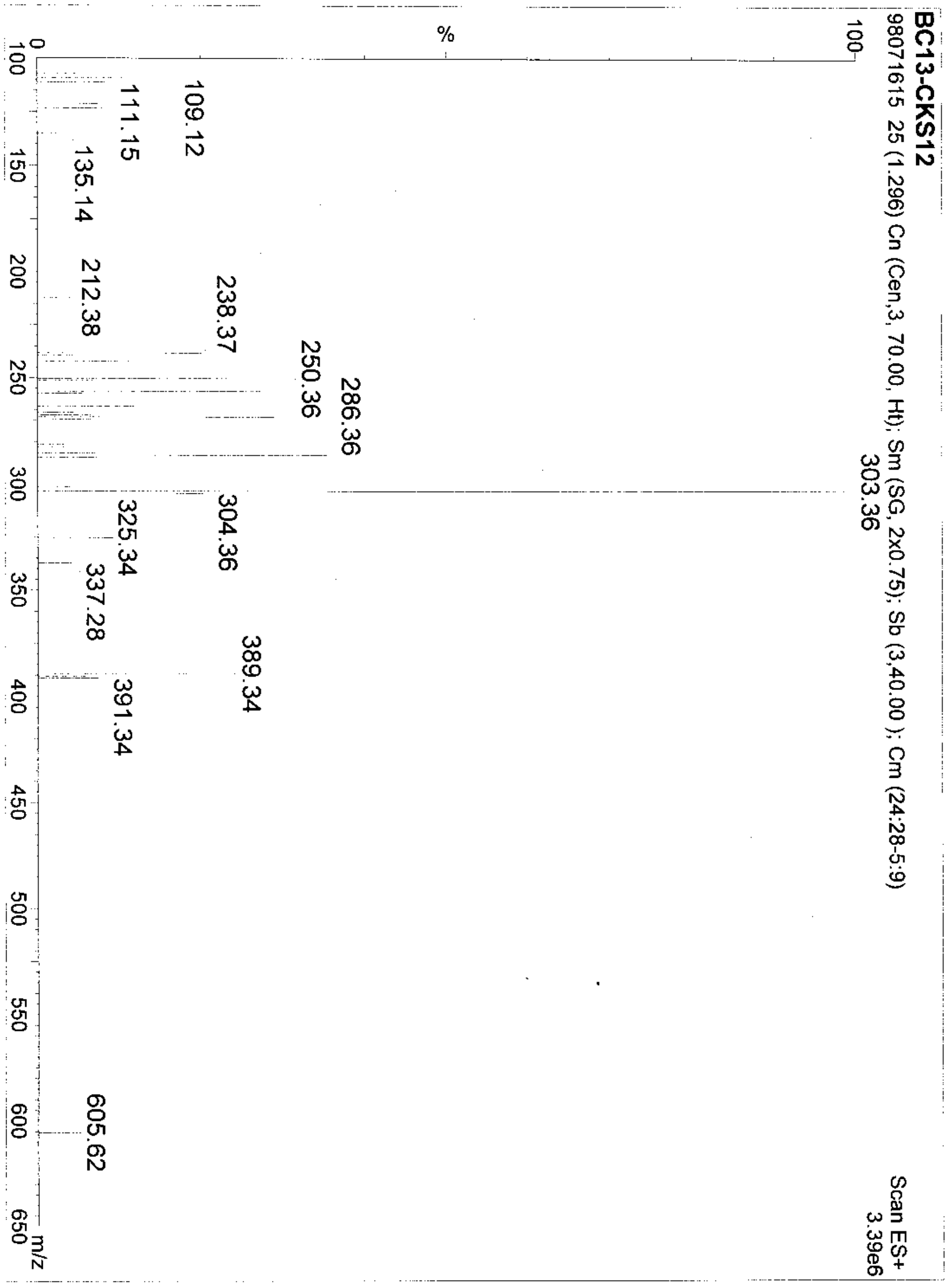
BC13-CKS12
9807161 5 25 (1.296) Cn (Cen,3, 70.00, Ht); Sm (SG, 2x0.75); Sb (3,40.00); Cm (24:28-5:9)
Scan ES+
3.39e6
100
%
0
303.36
286.36
250.36
238.37
212.38
109.12
111.15
135.14
304.36
325.34
337.28
389.34
391.34
605.62
100
150
200
250
300
350
400
450
500
550
600
650
m/z

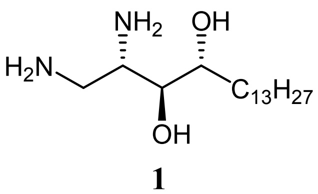
NH2
OH
H2N
C13H27
OH

1

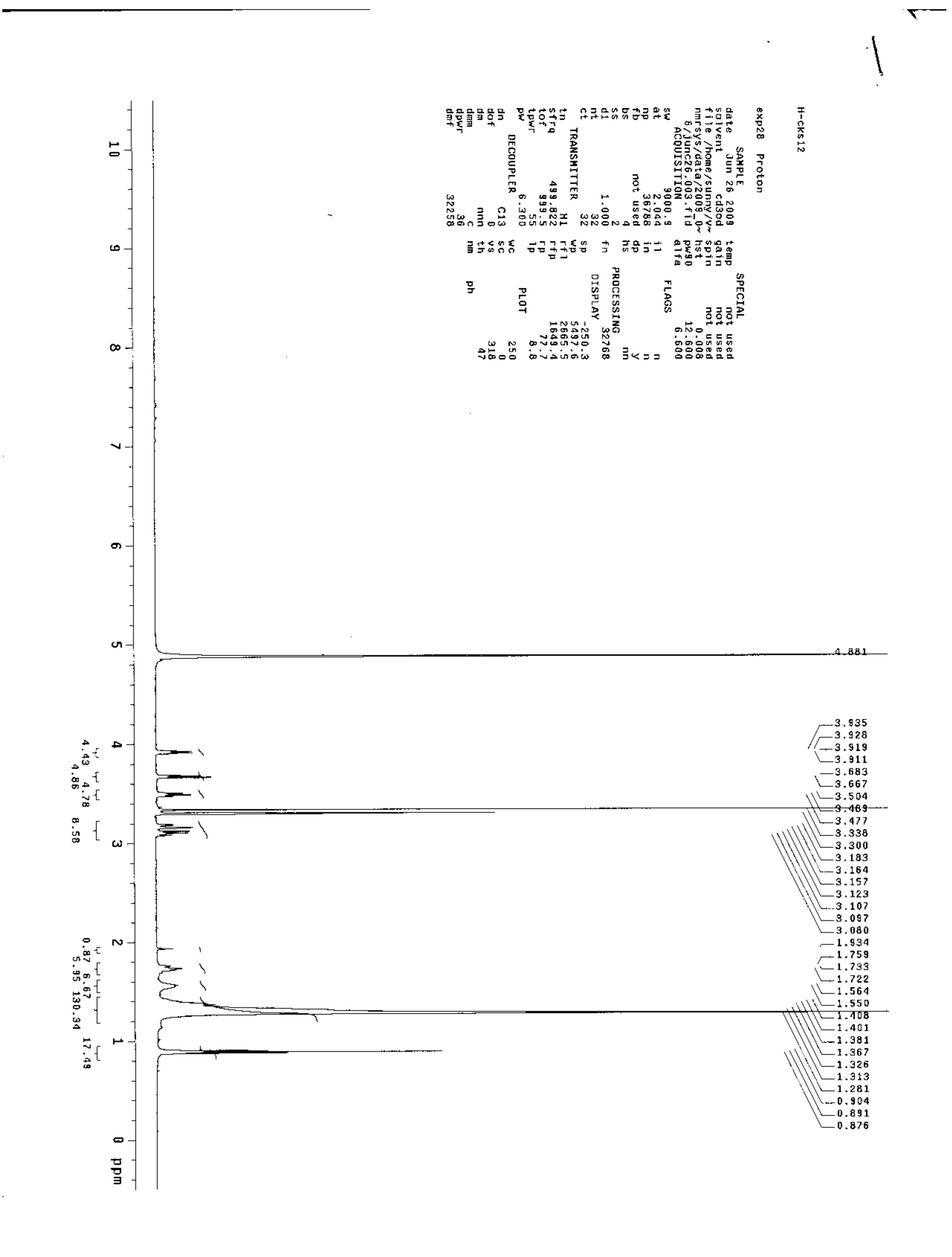
H-cks12
exp28 Proton
4.881
3.935
3.928
3.919
3.911
3.683
3.667
3.504
3.489
3.477
3.338
3.300
3.183
3.164
3.157
3.123
3.107
3.097
3.080
1.934
1.759
1.733
1.722
1.564
1.550
1.408
1.401
1.381
1.367
1.326
1.313
1.281
0.904
0.891
0.876
ppm

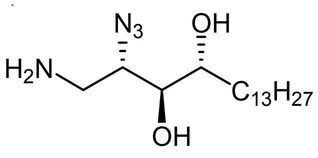
N3
OH
H2N
C13H27
OH

9

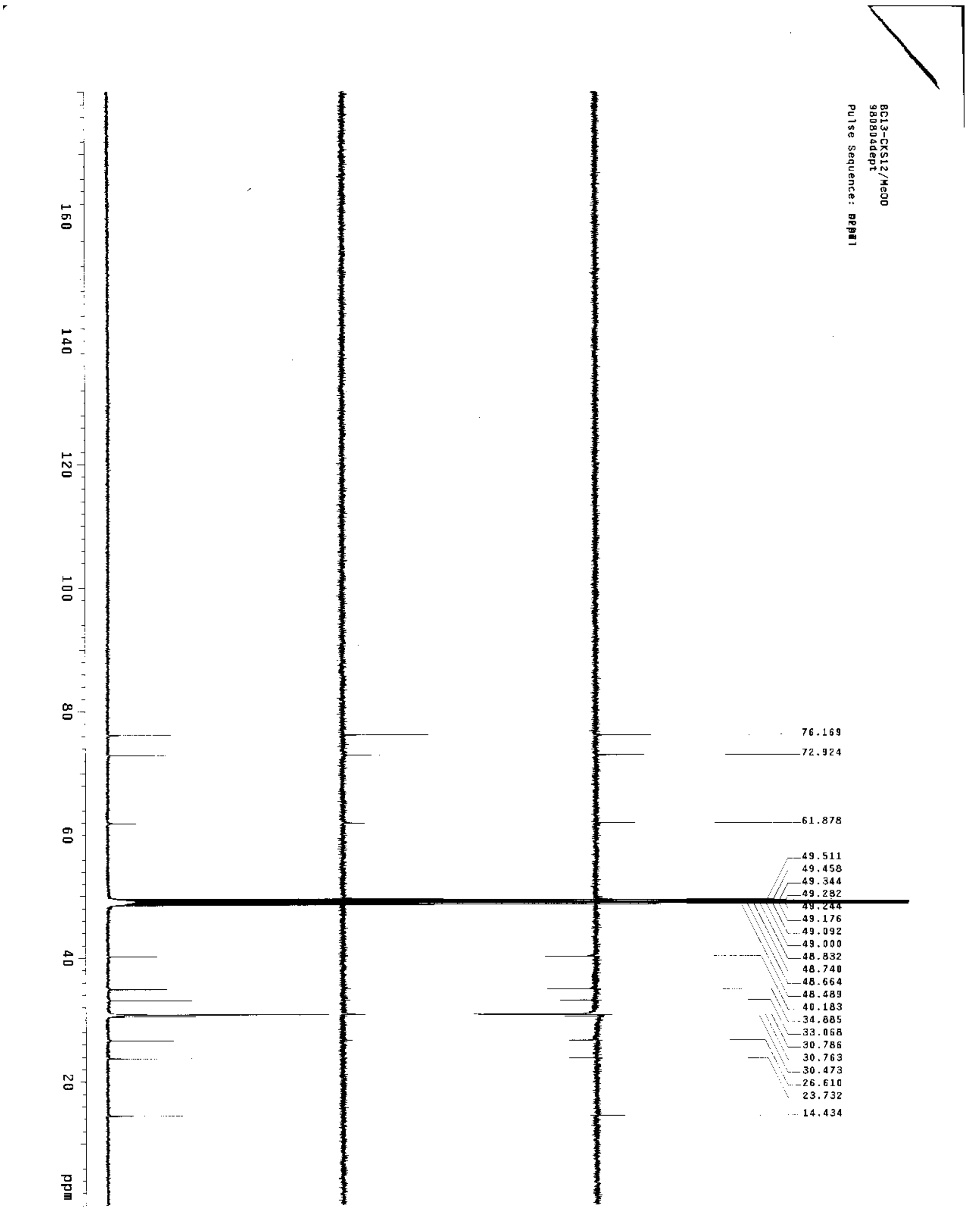
BC13-CKS12/MeOD
980804dept
Pulse Sequence:
76.169
72.924
61.878
49.511
49.458
49.344
49.282
49.244
49.176
49.092
49.000
48.832
48.740
48.664
48.489
40.183
34.885
33.068
30.786
30.763
30.473
26.610
23.732
14.434
160
140
120
100
80
60
40
20
ppm

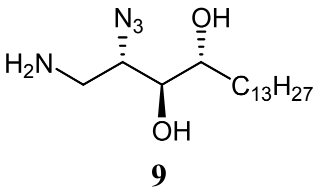
N3
OH
H2N
C13H27
OH

9

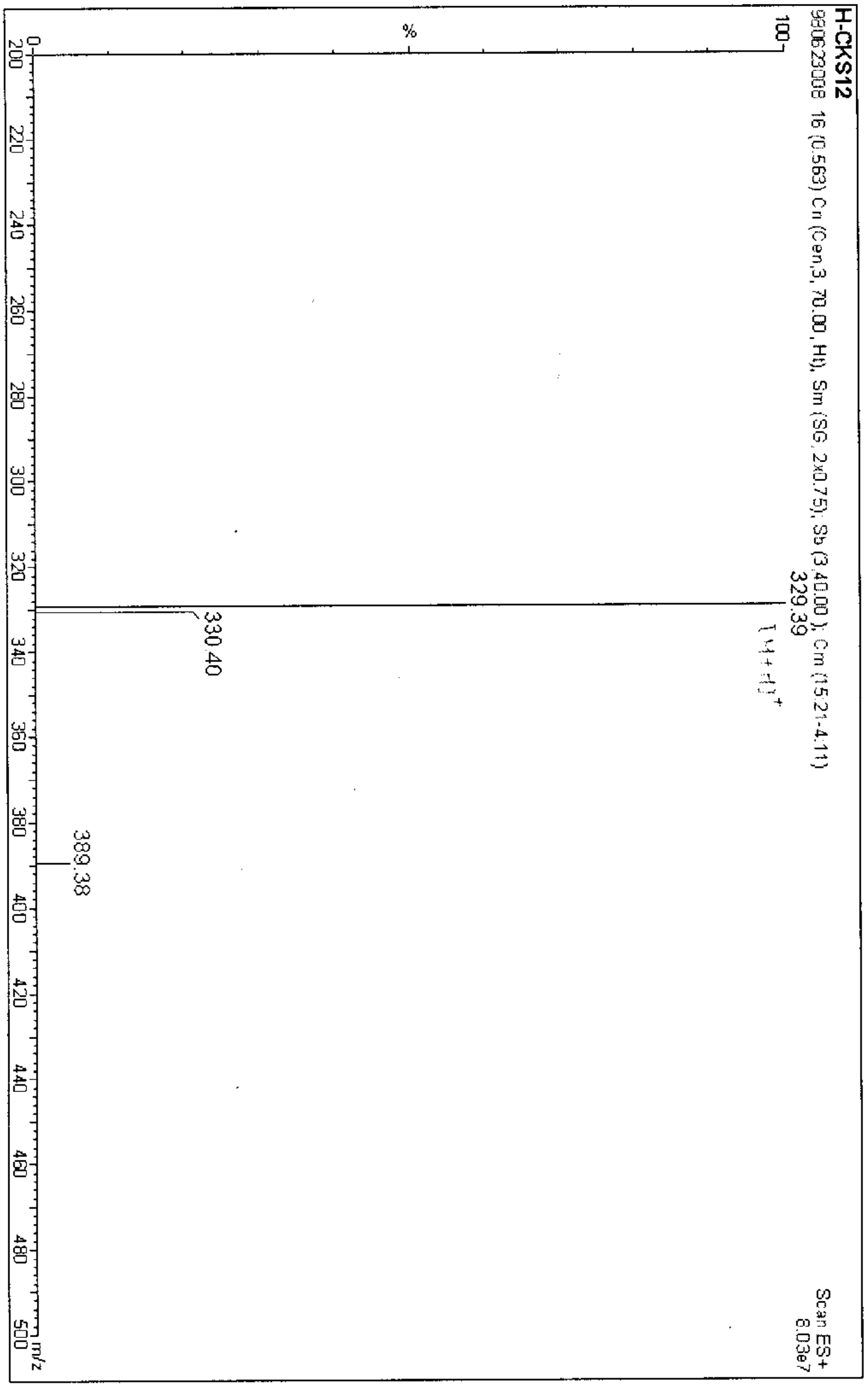
H-CKS12
98062308 16 (0.563) Cn (Cen,3, 70.00, Ht); Sm (SG, 2x0.75); Sb (3,40.00); Cm (15:21-4:11)
Scan ES+
8.03e7
100
%
0
329.39
[M+H]+
330.40
389.38
200
220
240
260
280
300
320
340
360
380
400
420
440
460
480
500
m/z

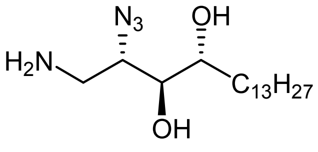
N3
OH
H2N
C13H27
OH

9

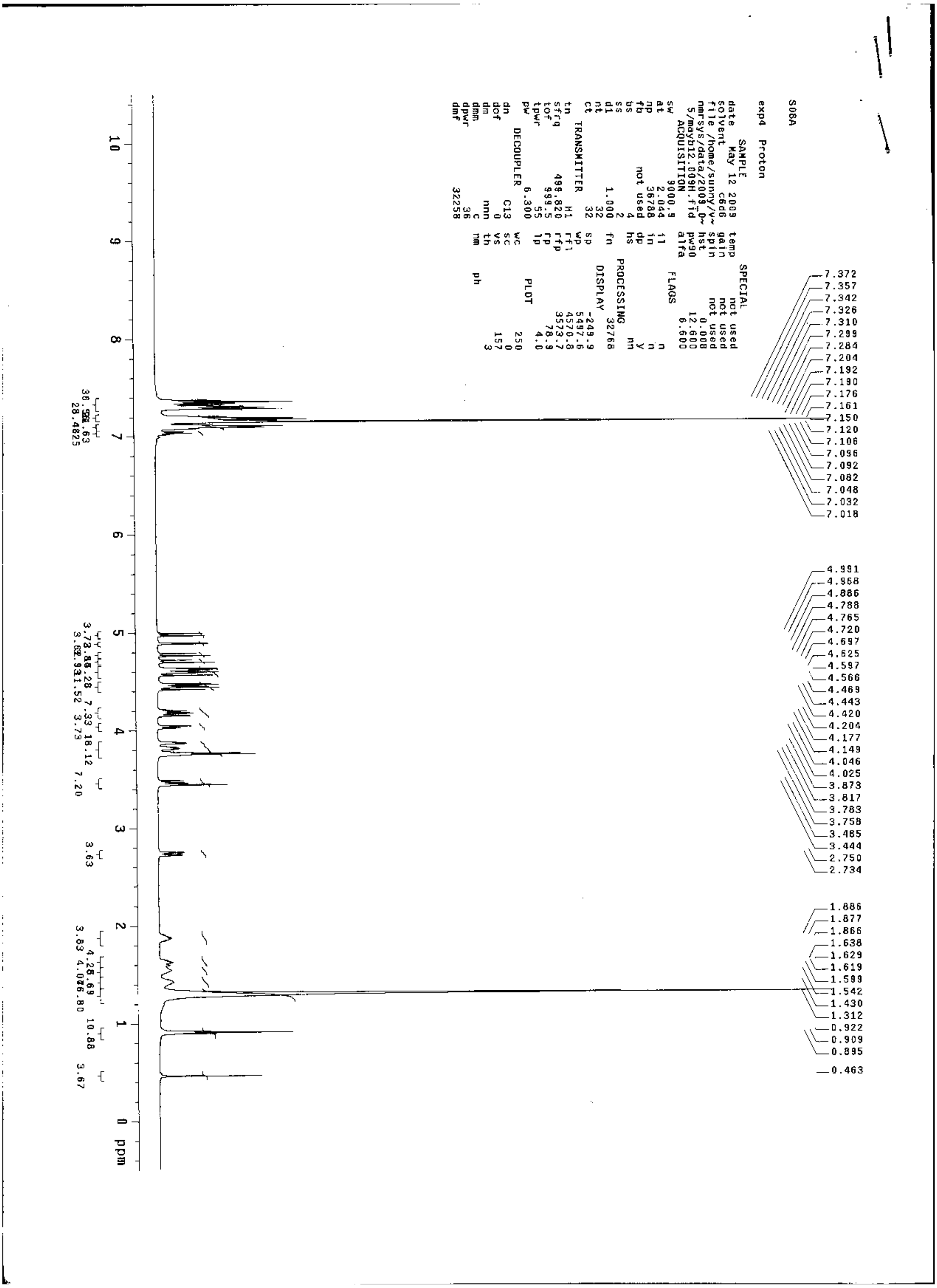

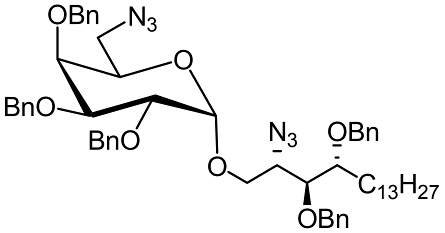

14α

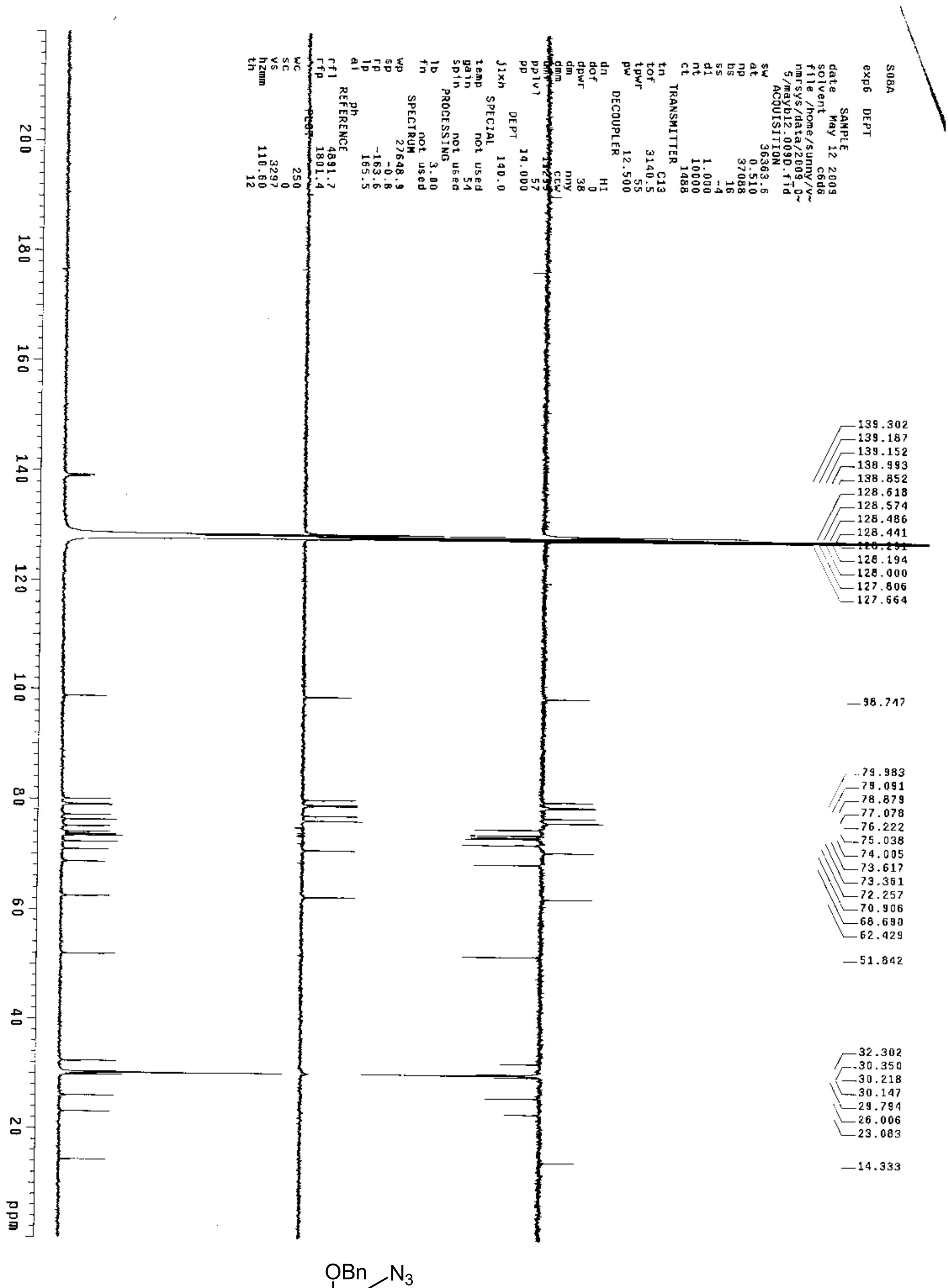
139.302
139.187
139.152
138.993
138.852
128.618
128.574
128.486
128.441
128.194
128.000
127.806
127.664
98.747
79.983
79.091
78.879
77.078
76.222
75.038
74.005
73.617
73.361
72.257
70.906
68.690
62.429
51.842
32.302
30.350
30.218
30.147
29.794
26.006
23.083
14.333
ppm

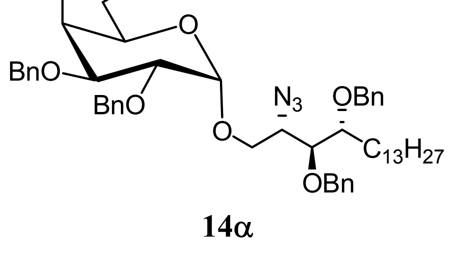
OBn
N3
O
BnO
BnO
N3
OBn
C13H27
OBn

14α

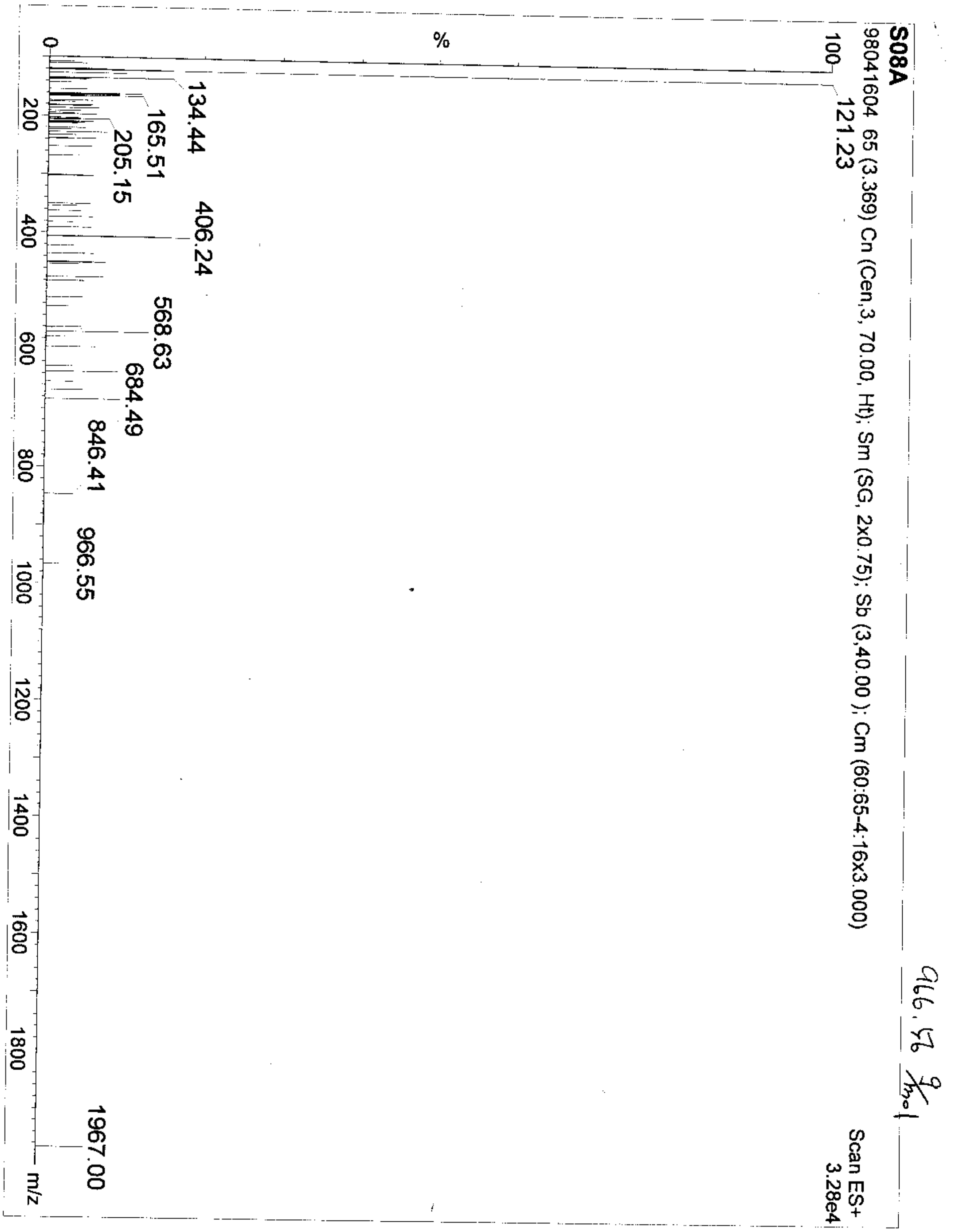
S08A
9804164 65 (3.369) Cn (Cen,3, 70.00, Ht); Sm (SG, 2x0.75); Sb (3,40.00); Cm (60:65-4:16x3.000)
Scan ES+
3.28e4
100
%
0
121.23
134.44
165.51
205.15
406.24
568.63
684.49
846.41
966.55
1967.00
200
400
600
800
1000
1200
1400
1600
1800
m/z

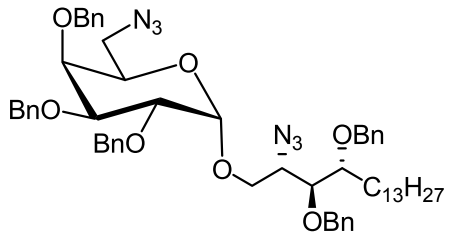
OBn
N3
O
BnO
BnO
N3
OBn
O
C13H27
OBn

14α

國立交通大學應用化學系

元素分析儀 Heraeus CHN-O Rapid 服務報告書

使用者姓名：廖國延　　　　　　　中心編號：980529

服務單位：清大原科 俞鐘山實驗室　樣品名稱或代號：SO8A

收件日期：　98　年　5　月　14　日 完成日期：　98　年　5　月　18　日

分析結果：

實驗值：	N%	C%	H%
1.	8.66	72.11	7.42
2.	8.46	71.79	7.27
3.			
4.			
推測值：	8.69	72.02	7.71

本日所使用之 Standard：A

(A)Acetanilide　(B)Atropin　(C)N-Anilin

	N%	C%	H%
理論值：	10.36	71.09	6.71
測出值：	10.34	71.01	7.09

備註：

費用核算：NCH：800 元

報告日期：　98　年　5　月　19　日

儀器負責人簽章：[illegible]　　技術員簽章：技士李蘊明

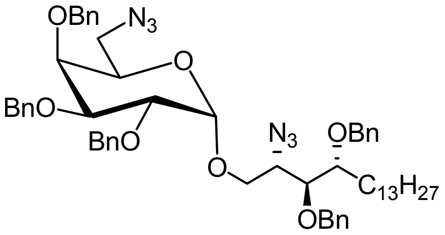

14α

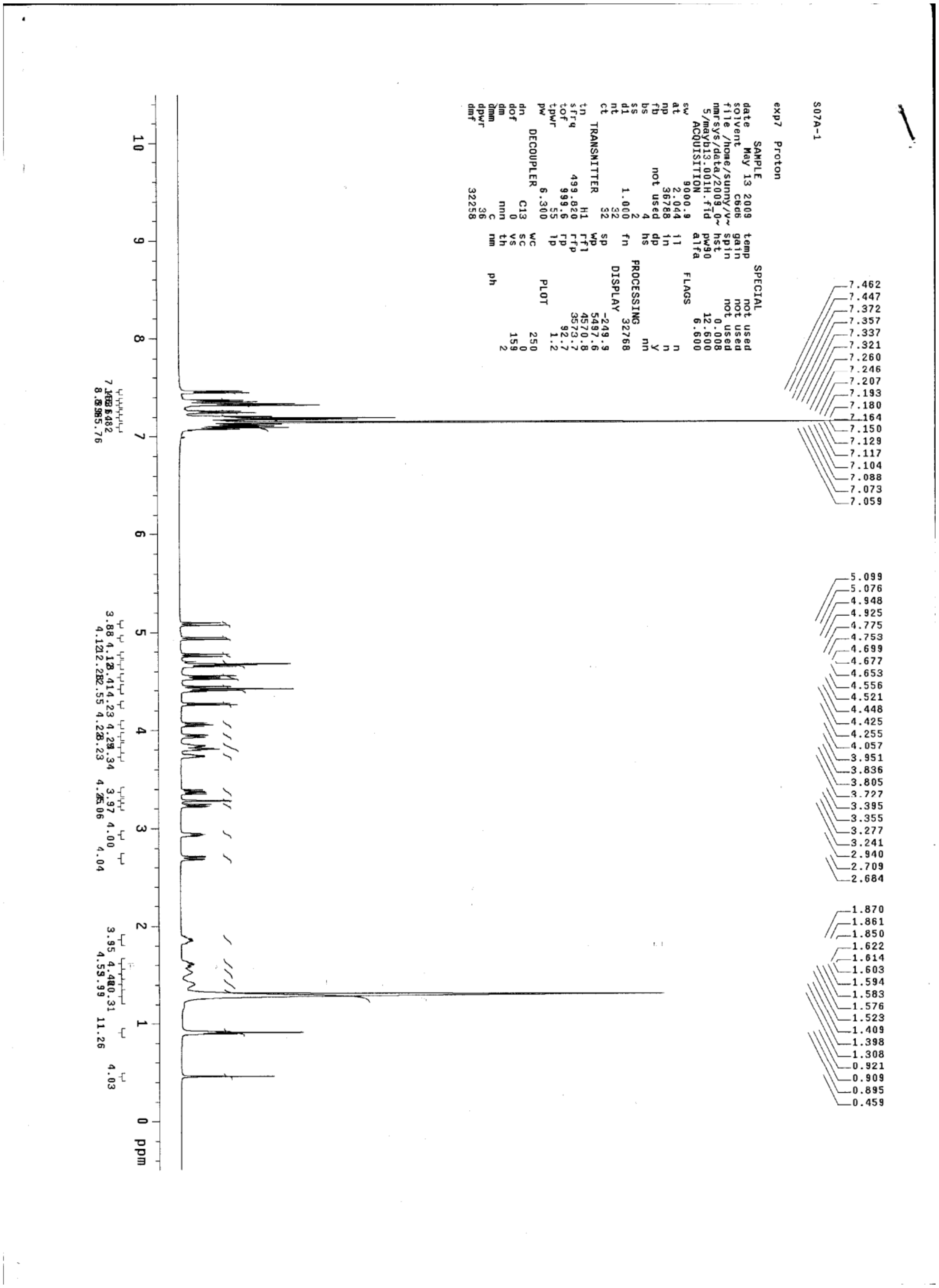

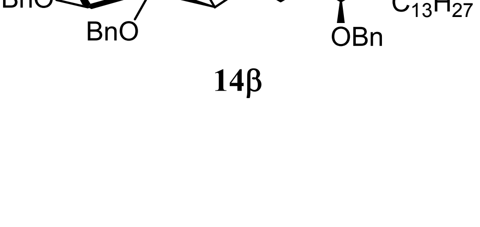

14β

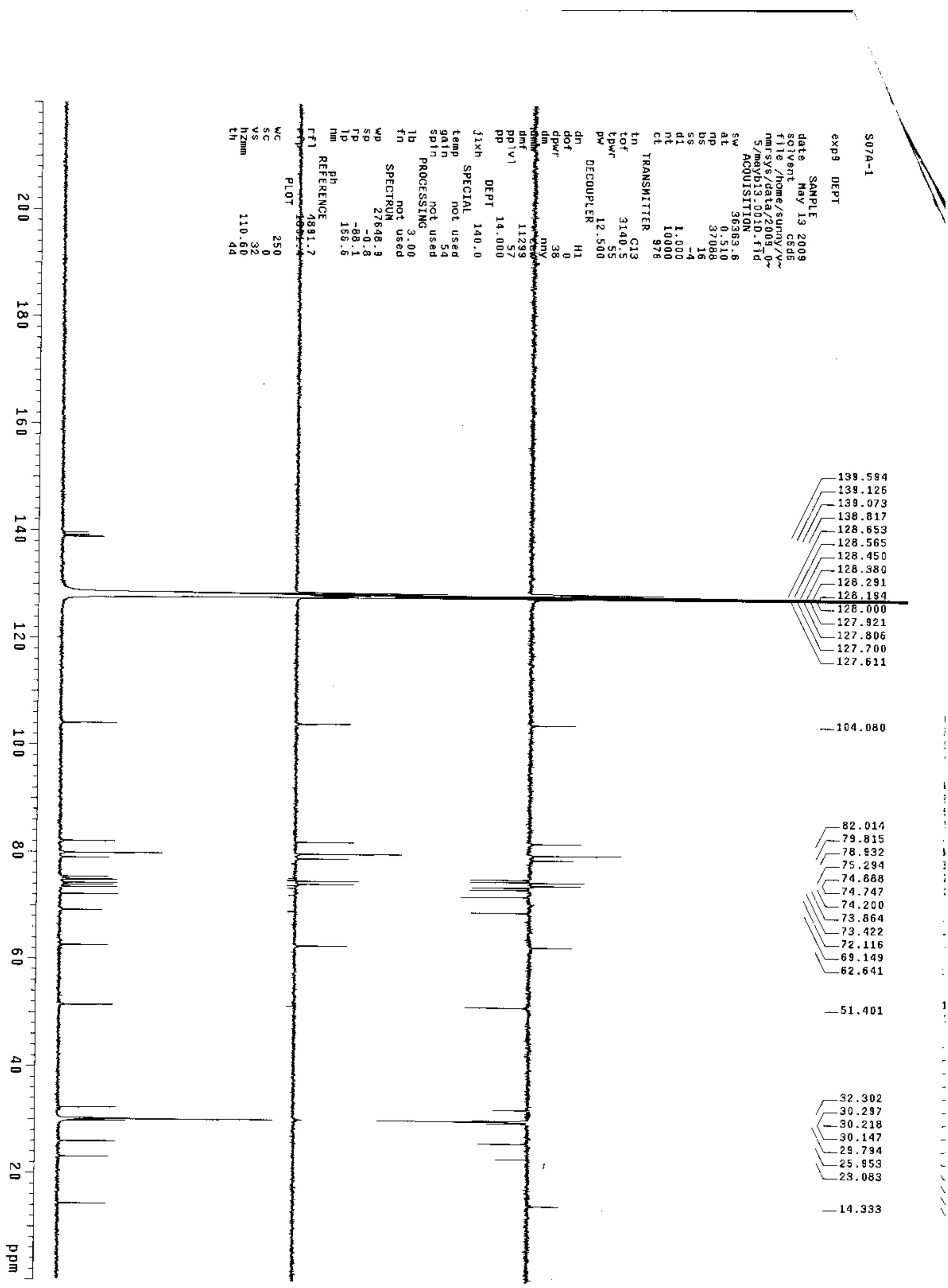
S07A-1
exp9 DEPT
139.594
139.126
139.073
138.817
128.653
128.565
128.450
128.380
128.291
128.194
128.000
127.921
127.806
127.700
127.611
104.080
82.014
79.815
78.932
75.294
74.888
74.747
74.200
73.864
73.422
72.116
69.149
62.641
51.401
32.302
30.297
30.218
30.147
29.794
25.953
23.083
14.333
ppm

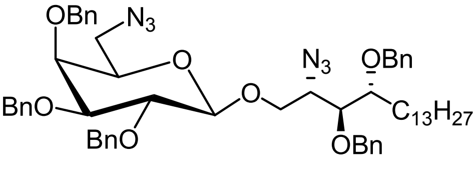
OBn
N3
O
BnO
BnO
O
N3
OBn
C13H27
OBn

14β

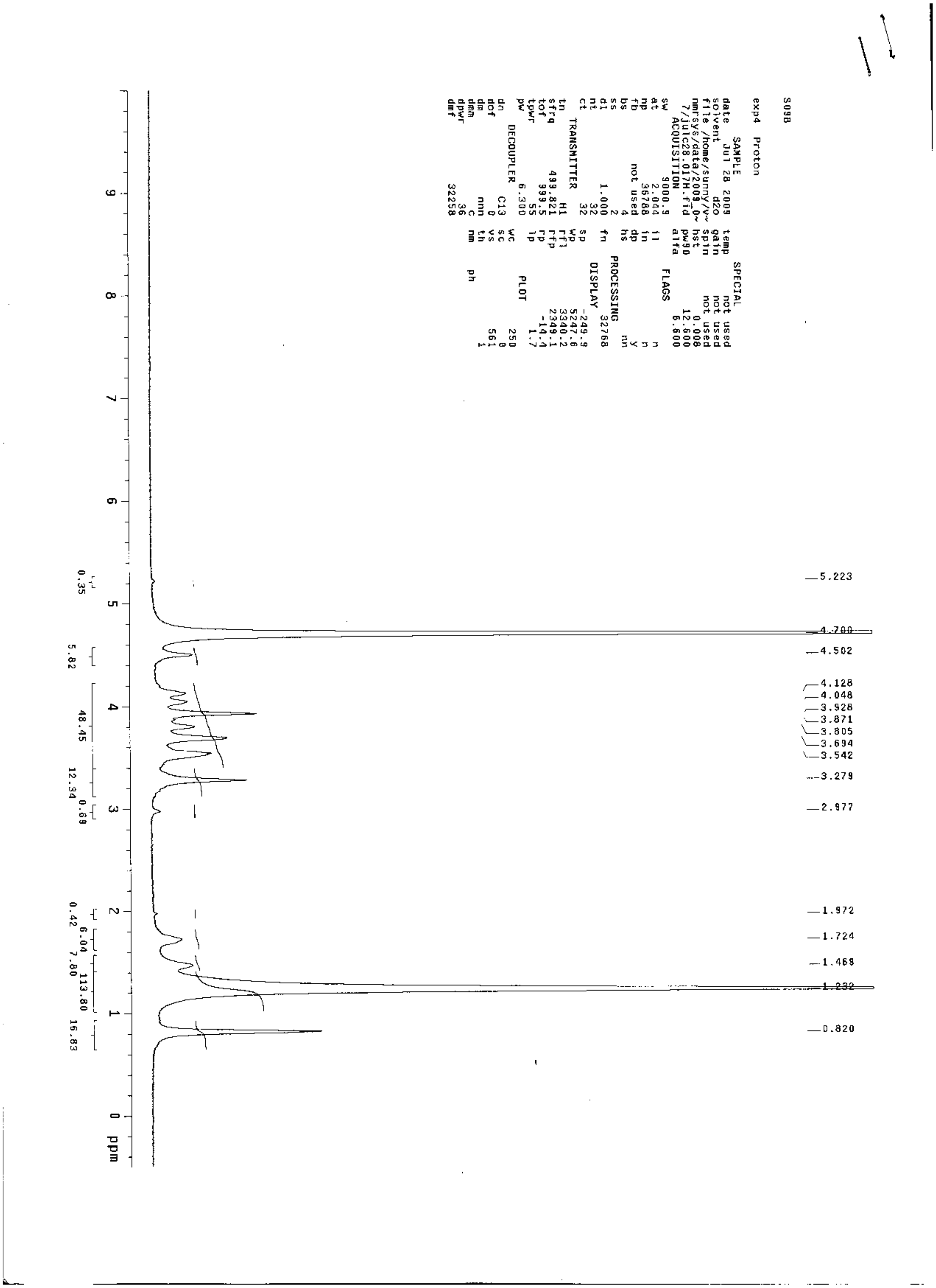
S09B
exp4 Proton
5.223
4.700
4.502
4.128
4.048
3.928
3.871
3.805
3.694
3.542
3.279
2.977
1.972
1.724
1.469
1.232
0.820
ppm

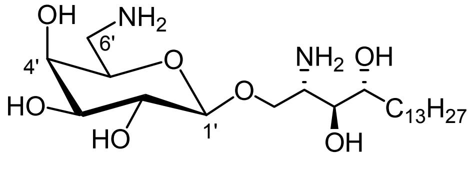
OH
NH2
6'
4'
O
NH2
OH
HO
O
C13H27
HO
1'
OH

17β

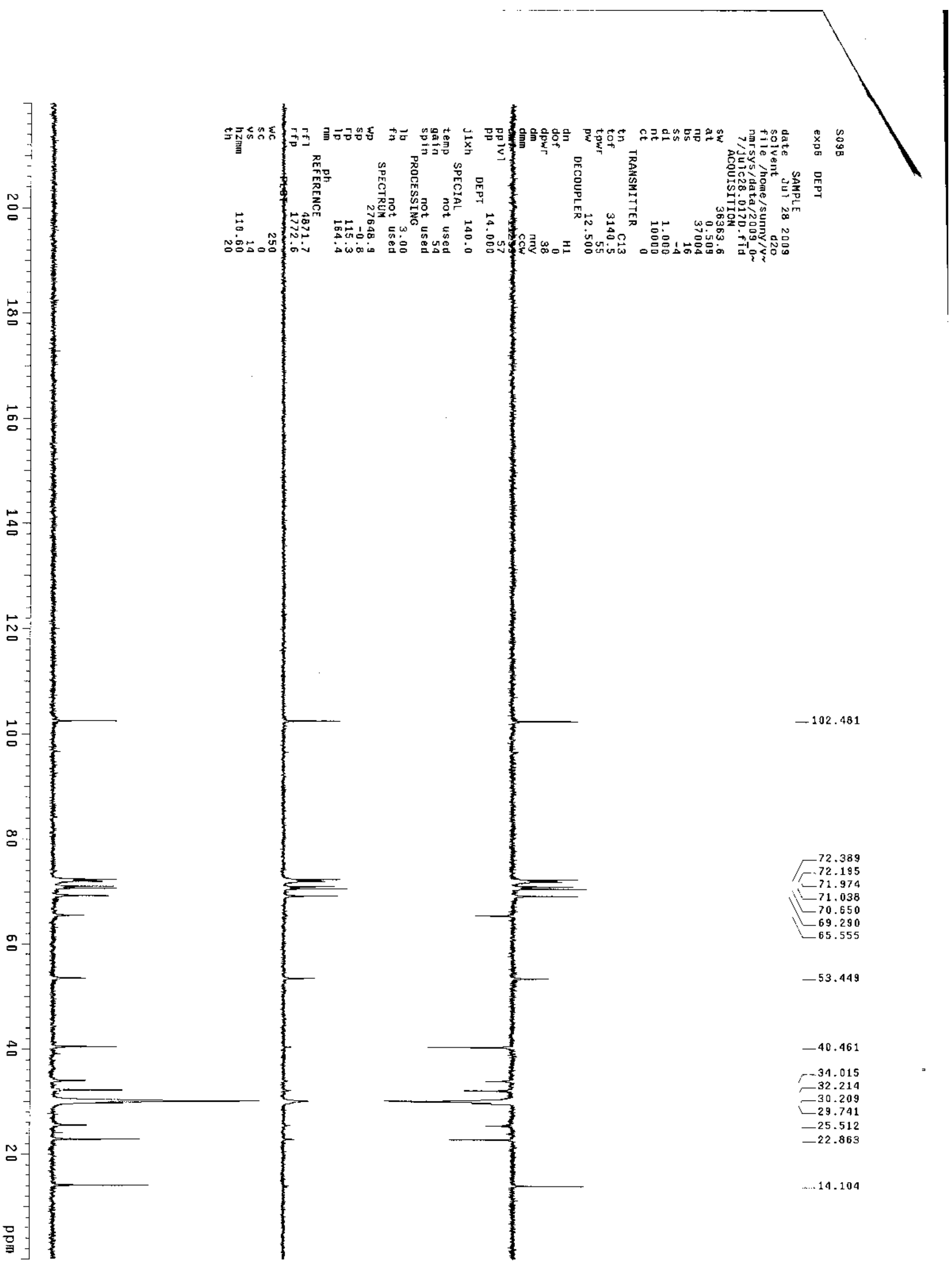

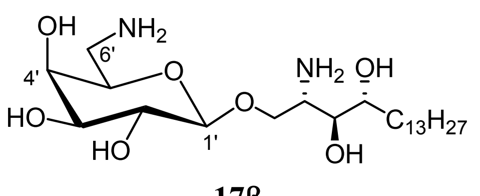

17β

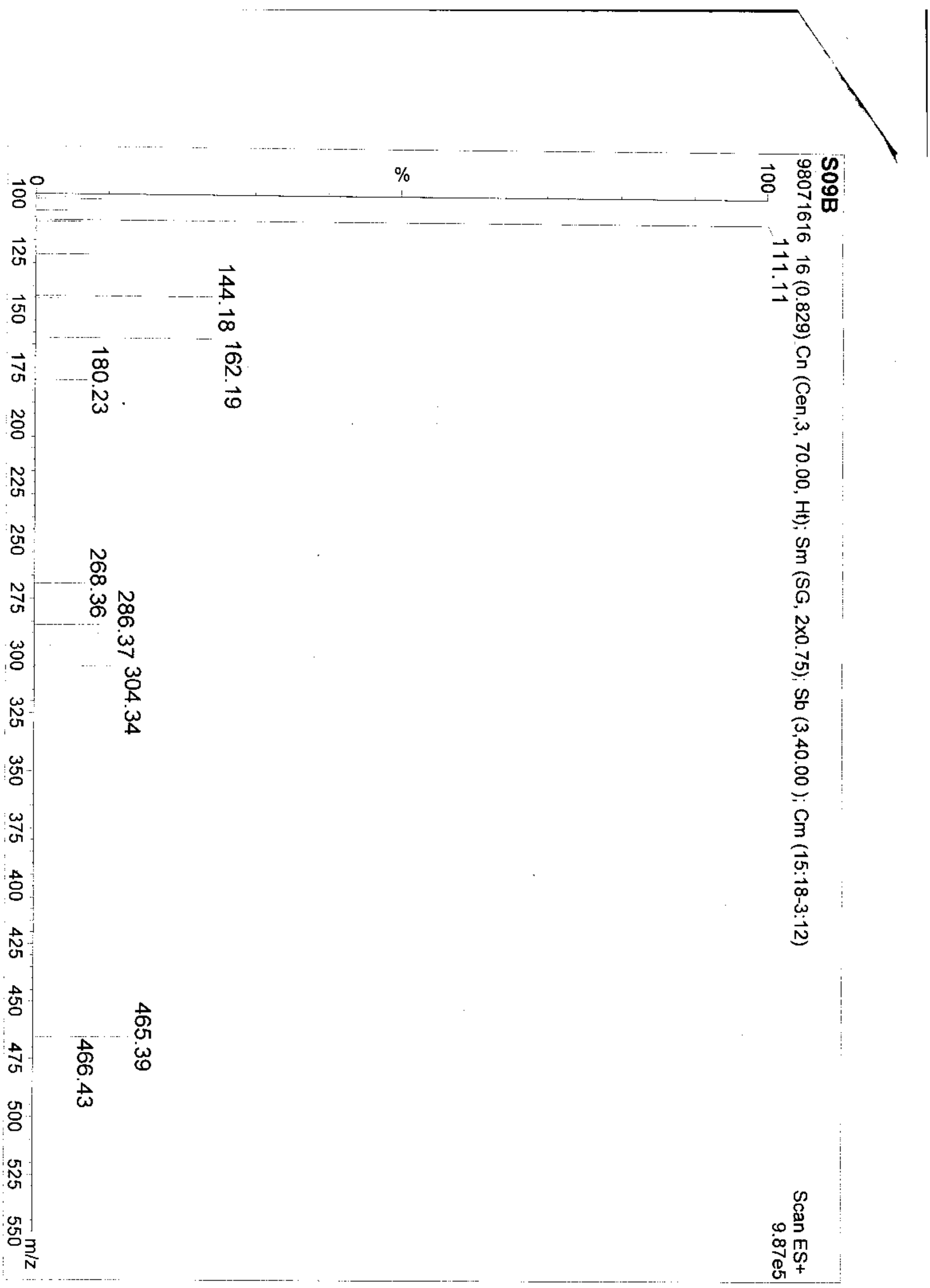
S09B
9807161616 (0.829) Cn (Cen,3, 70.00, Ht); Sm (SG, 2x0.75); Sb (3,40.00); Cm (15:18-3:12)
Scan ES+
9.87e5
100
%
0
111.11
144.18
162.19
180.23
268.36
286.37
304.34
465.39
466.43
100
125
150
175
200
225
250
275
300
325
350
375
400
425
450
475
500
525
550
m/z

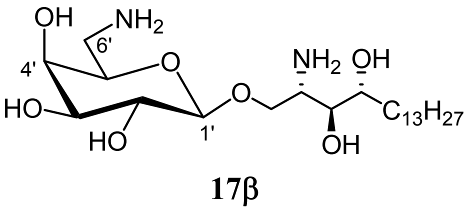
OH
NH2
6'
O
4'
HO
HO
1'
O
NH2
OH
OH
C13H27

17β

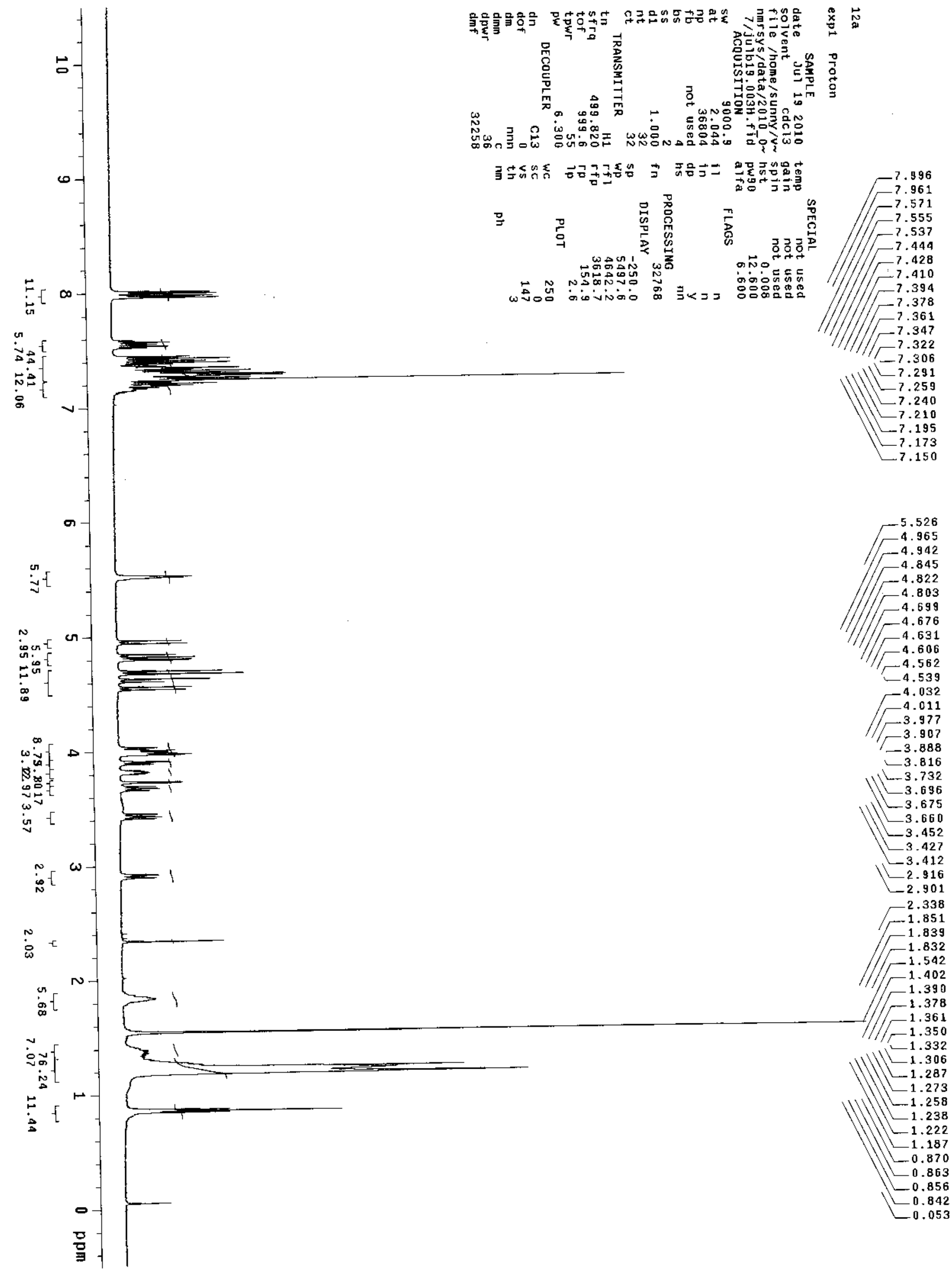

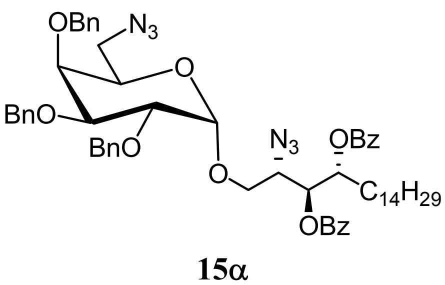

15α

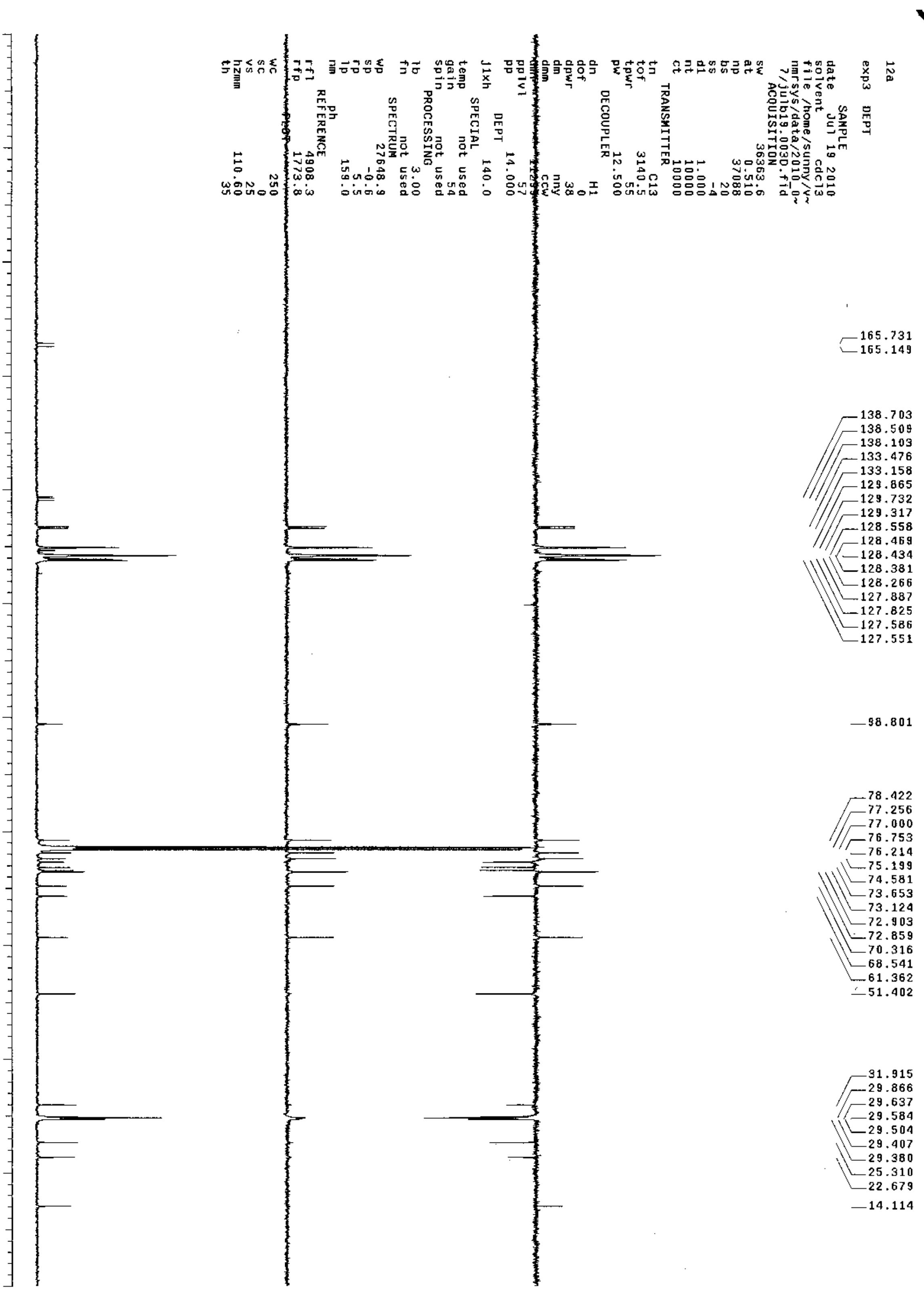

exp3 DEPT
165.731
165.149
138.703
138.509
138.103
133.476
133.158
129.865
129.732
129.317
128.558
128.469
128.434
128.381
128.266
127.887
127.825
127.586
127.551
98.801
78.422
77.256
77.000
76.753
76.214
75.199
74.581
73.653
73.124
72.903
72.859
70.316
68.541
61.362
51.402
31.915
29.866
29.637
29.584
29.504
29.407
29.380
25.310
22.679
14.114
ppm

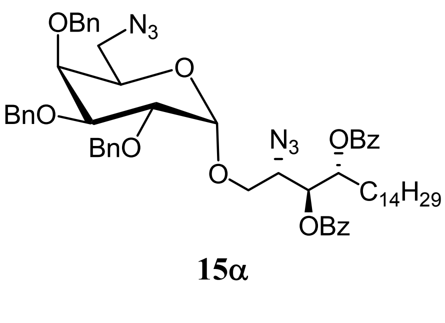

OBn
N3
O
BnO
BnO
N3
OBz
O
C14H29
OBz

15α

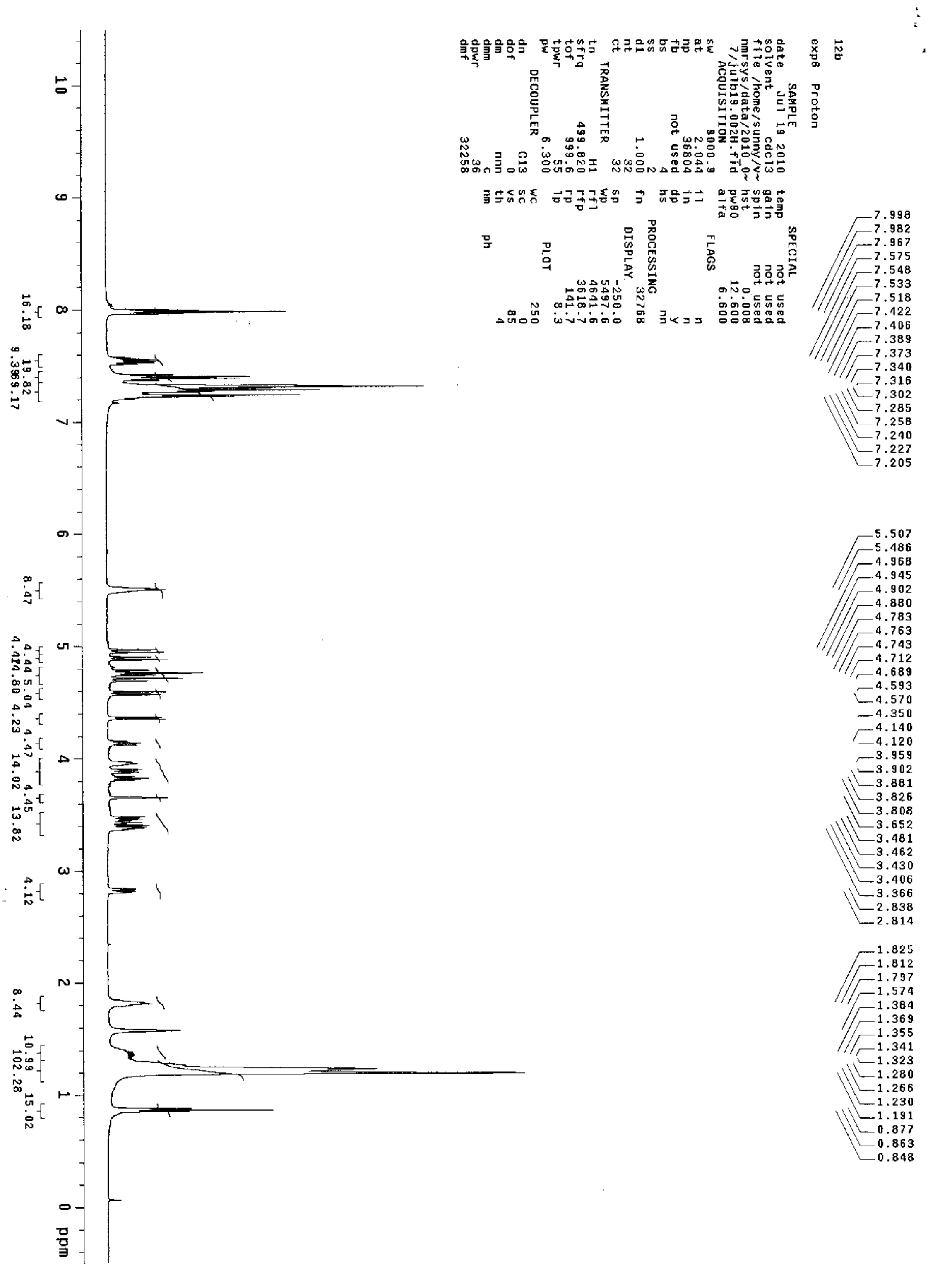

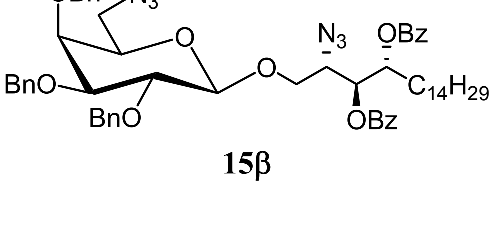

15β

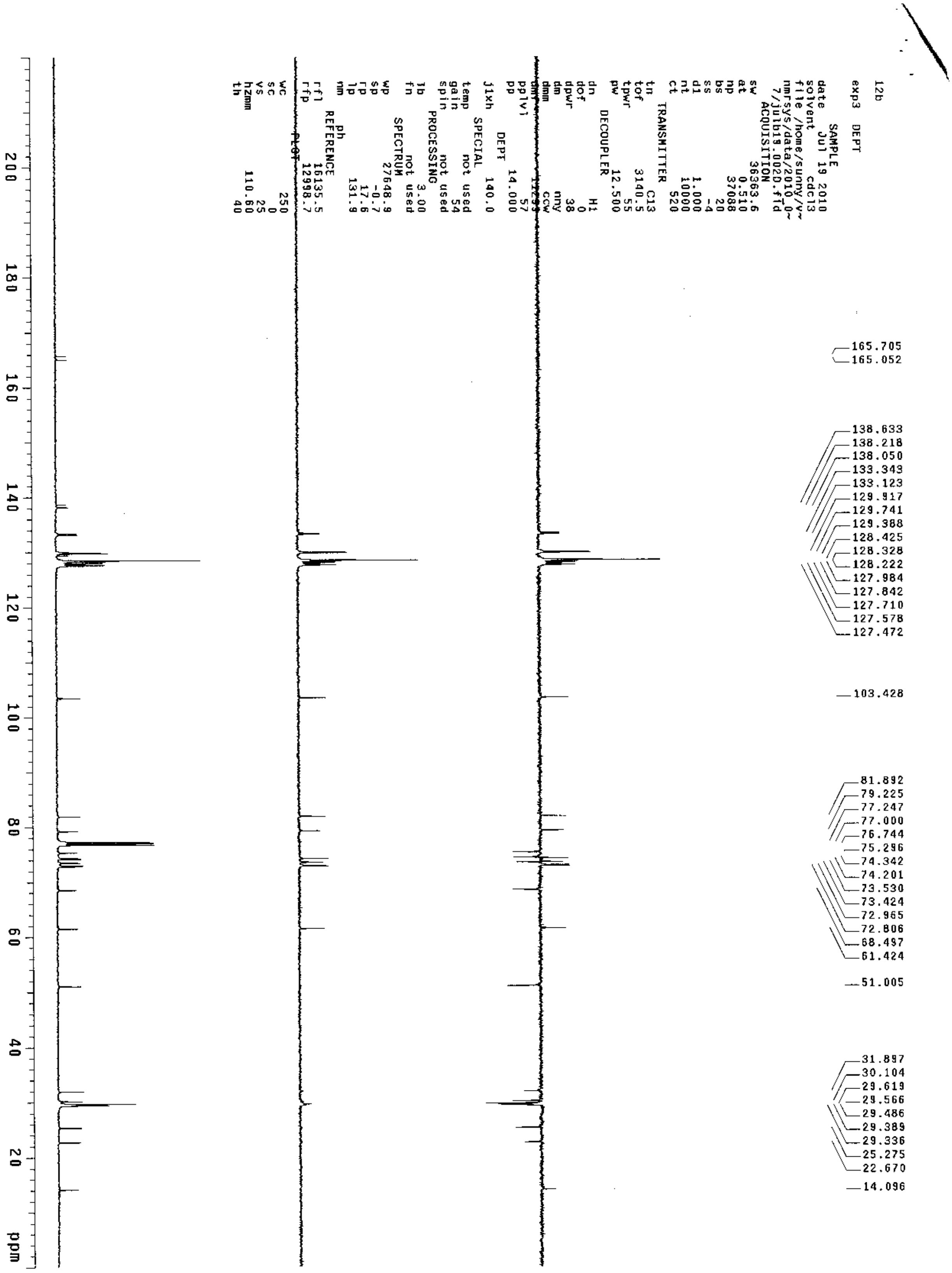
exp3 DEPT
165.705
165.052
138.633
138.218
138.050
133.343
133.123
129.917
129.741
129.388
128.425
128.328
128.222
127.984
127.842
127.710
127.578
127.472
103.428
81.892
79.225
77.247
77.000
76.744
75.296
74.342
74.201
73.530
73.424
72.965
72.806
68.497
61.424
51.005
31.897
30.104
29.619
29.566
29.486
29.389
29.336
25.275
22.670
14.096
ppm

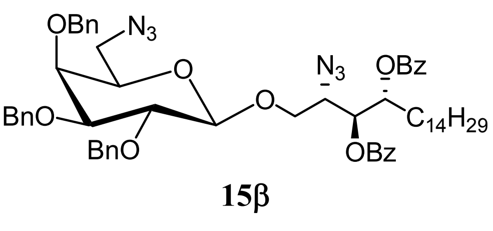
OBn
N_3
O
N_3
OBz
BnO
O
$C_{14}H_{29}$
BnO
OBz

15β

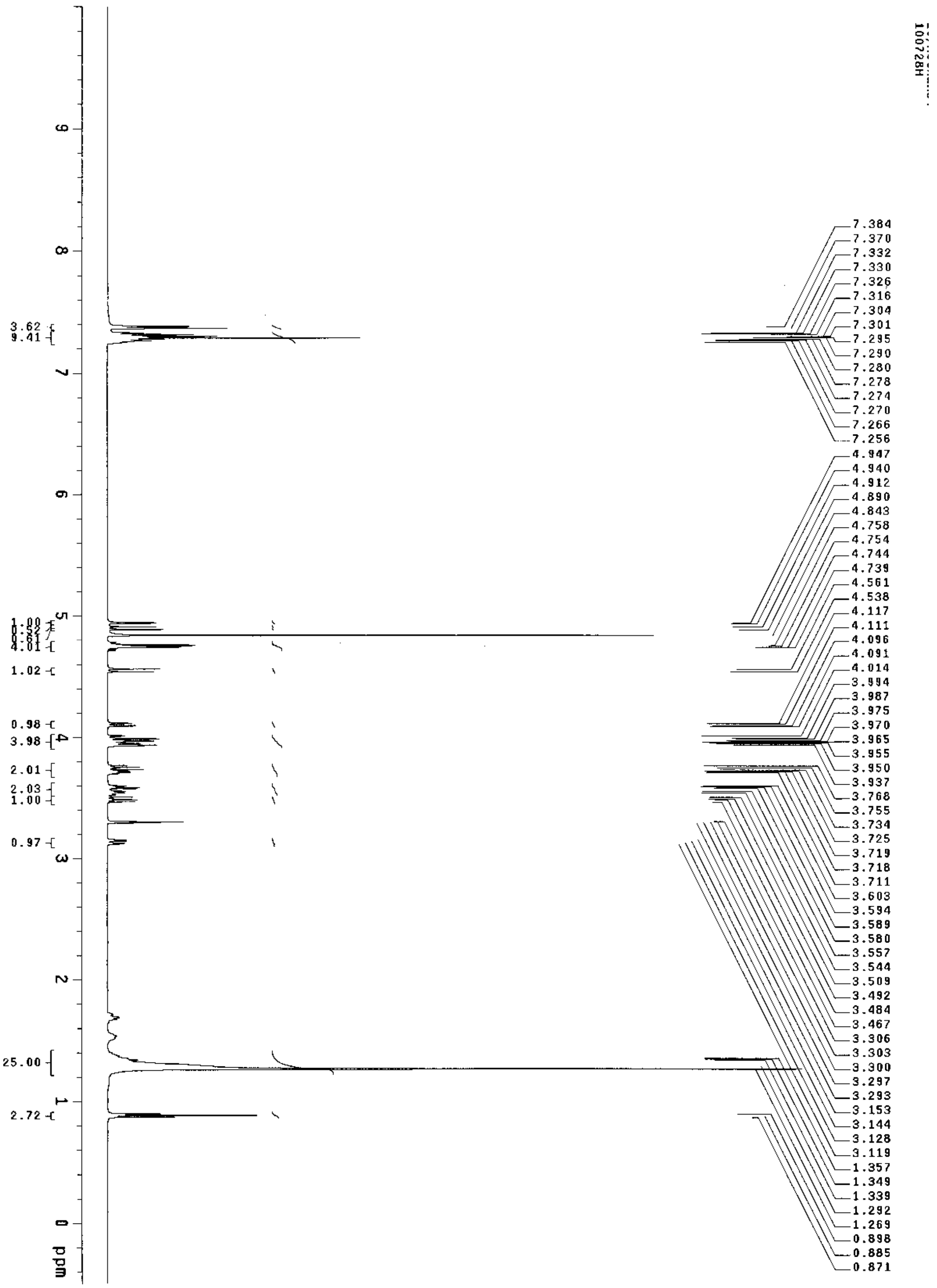
100728H
9
8
7
6
5
4
3
2
1
0
ppm
3.62
9.41
1.00
0.52
0.61
4.01
1.02
0.98
3.98
2.01
2.03
1.00
0.97
25.00
2.72
7.384
7.370
7.332
7.330
7.326
7.316
7.304
7.301
7.295
7.290
7.280
7.278
7.274
7.270
7.266
7.256
4.947
4.940
4.912
4.890
4.843
4.758
4.754
4.744
4.739
4.561
4.538
4.117
4.111
4.096
4.091
4.014
3.994
3.987
3.975
3.970
3.965
3.955
3.950
3.937
3.768
3.755
3.734
3.725
3.719
3.718
3.711
3.603
3.594
3.589
3.580
3.557
3.544
3.509
3.492
3.484
3.467
3.306
3.303
3.300
3.297
3.293
3.153
3.144
3.128
3.119
1.357
1.349
1.339
1.292
1.269
0.898
0.885
0.871

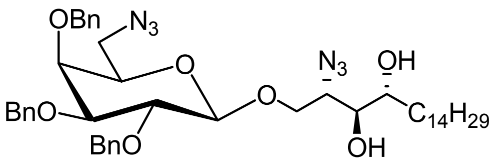
OBn
N3
O
BnO
BnO
O
N3
OH
OH
C14H29

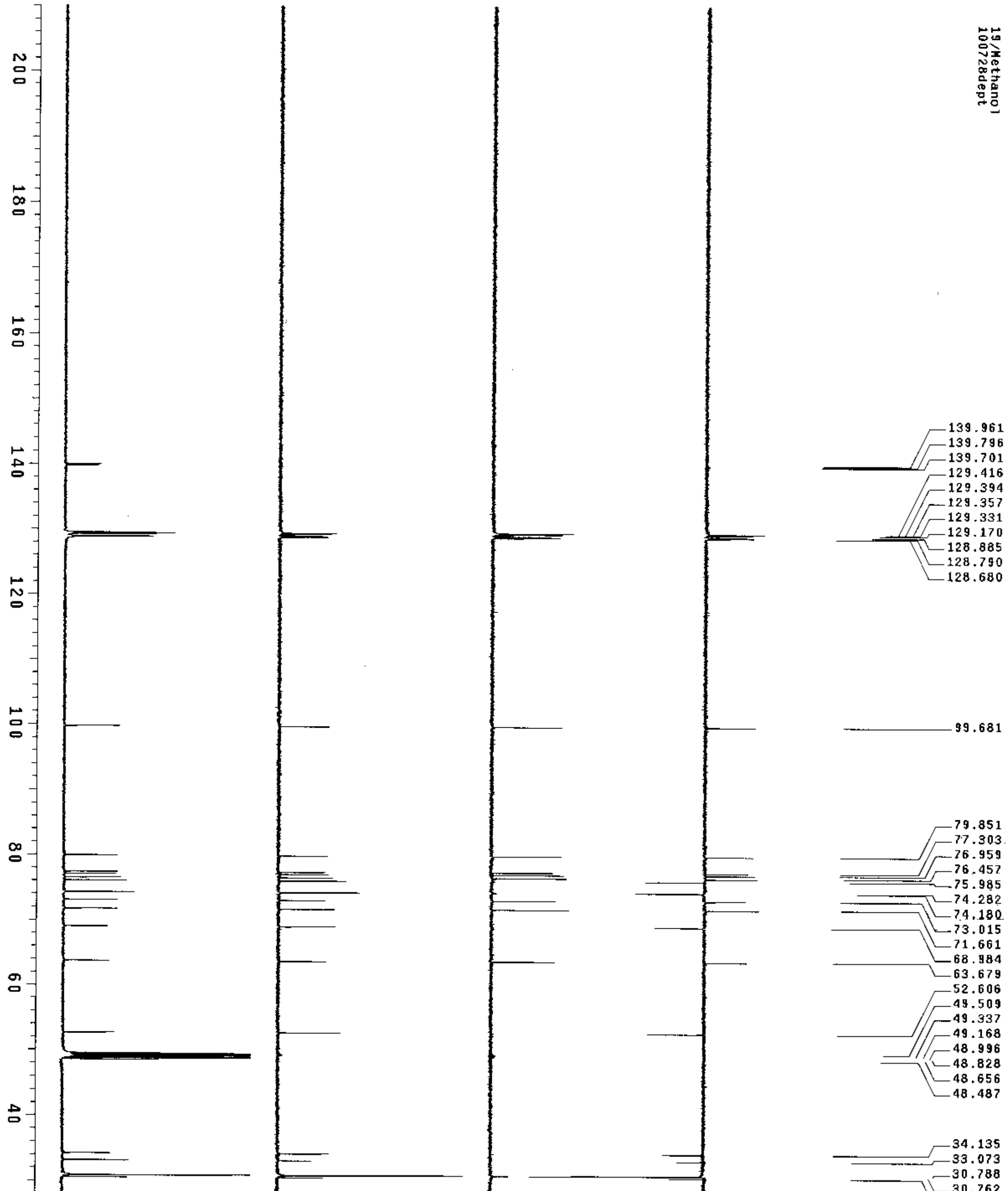
19/Methanol
100728dept
ppm
200
180
160
140
120
100
80
60
40
20
139.961
139.796
139.701
129.416
129.394
129.357
129.331
129.170
128.885
128.790
128.680
99.681
79.851
77.303
76.959
76.457
75.985
74.282
74.180
73.015
71.661
68.984
63.679
52.606
49.509
49.337
49.168
48.996
48.828
48.656
48.487
34.135
33.073
30.788
30.762
30.477
26.742
23.736
14.458

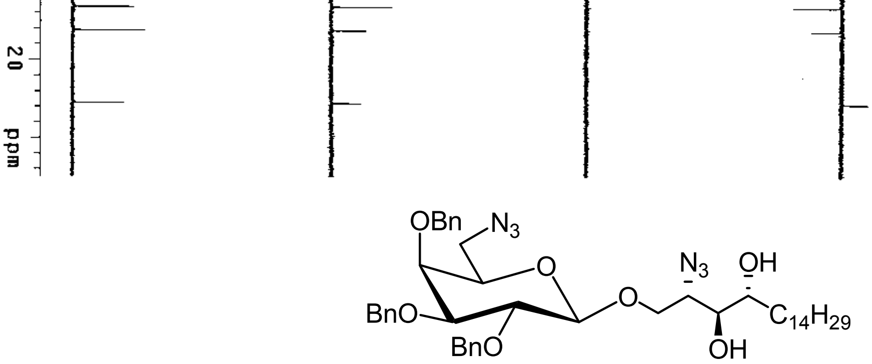
OBn
N3
O
BnO
BnO
O
N3
OH
OH
C14H29

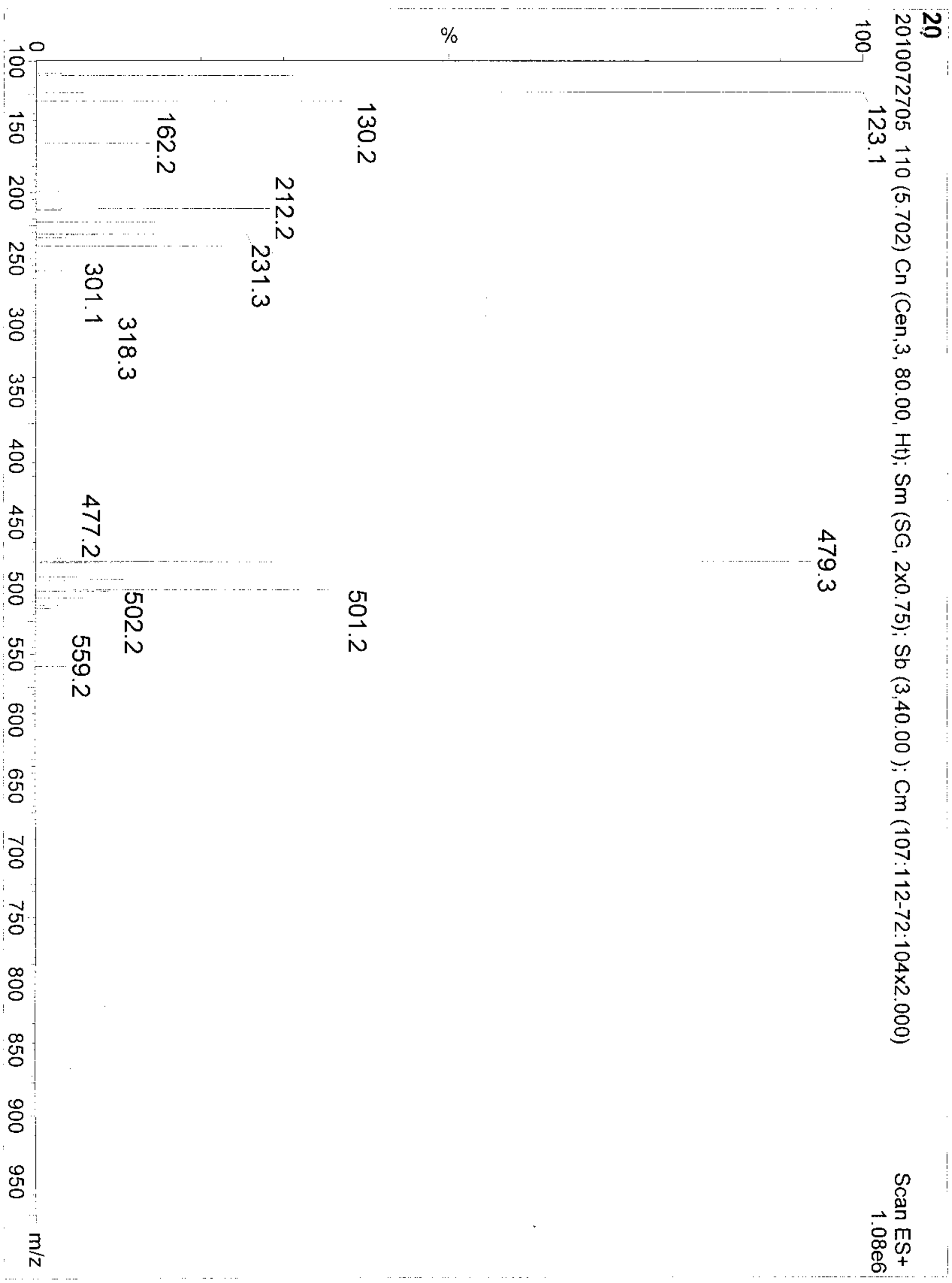
20
2010072705 110 (5.702) Cn (Cen,3, 80.00, Ht); Sm (SG, 2x0.75); Sb (3,40.00); Cm (107:112-72:104x2.000)
Scan ES+
1.08e6
100
%
0
123.1
130.2
162.2
212.2
231.3
301.1
318.3
477.2
479.3
501.2
502.2
559.2
100
150
200
250
300
350
400
450
500
550
600
650
700
750
800
850
900
950
m/z

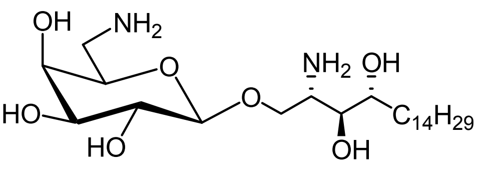
OH
NH2
O
O
NH2
OH
HO
HO
C14H29
OH

2

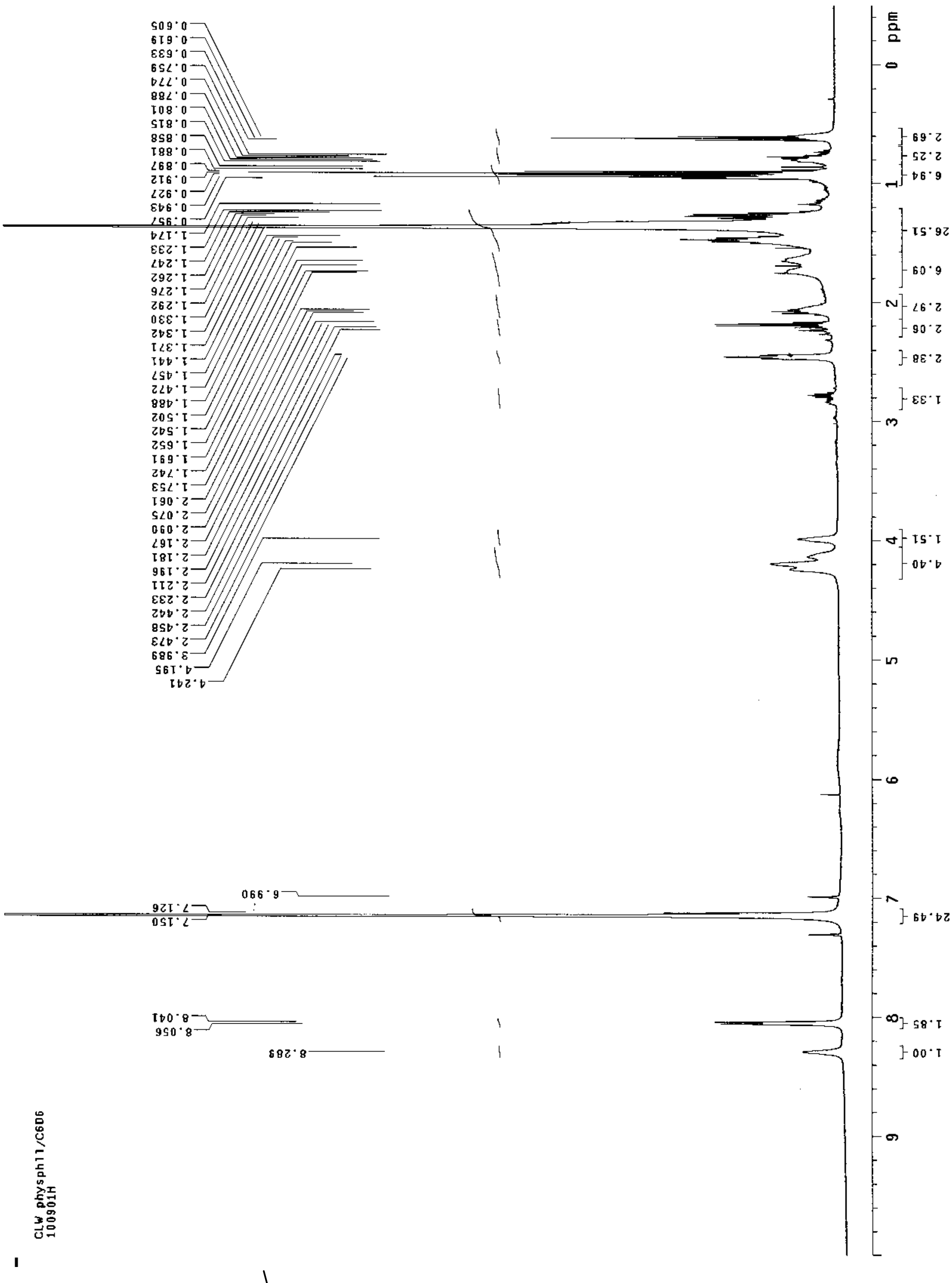

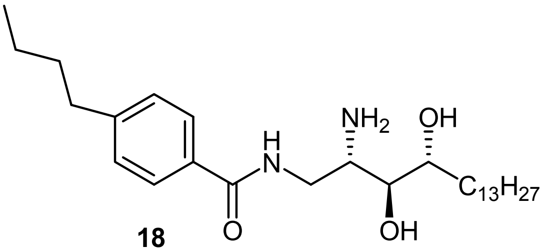

18

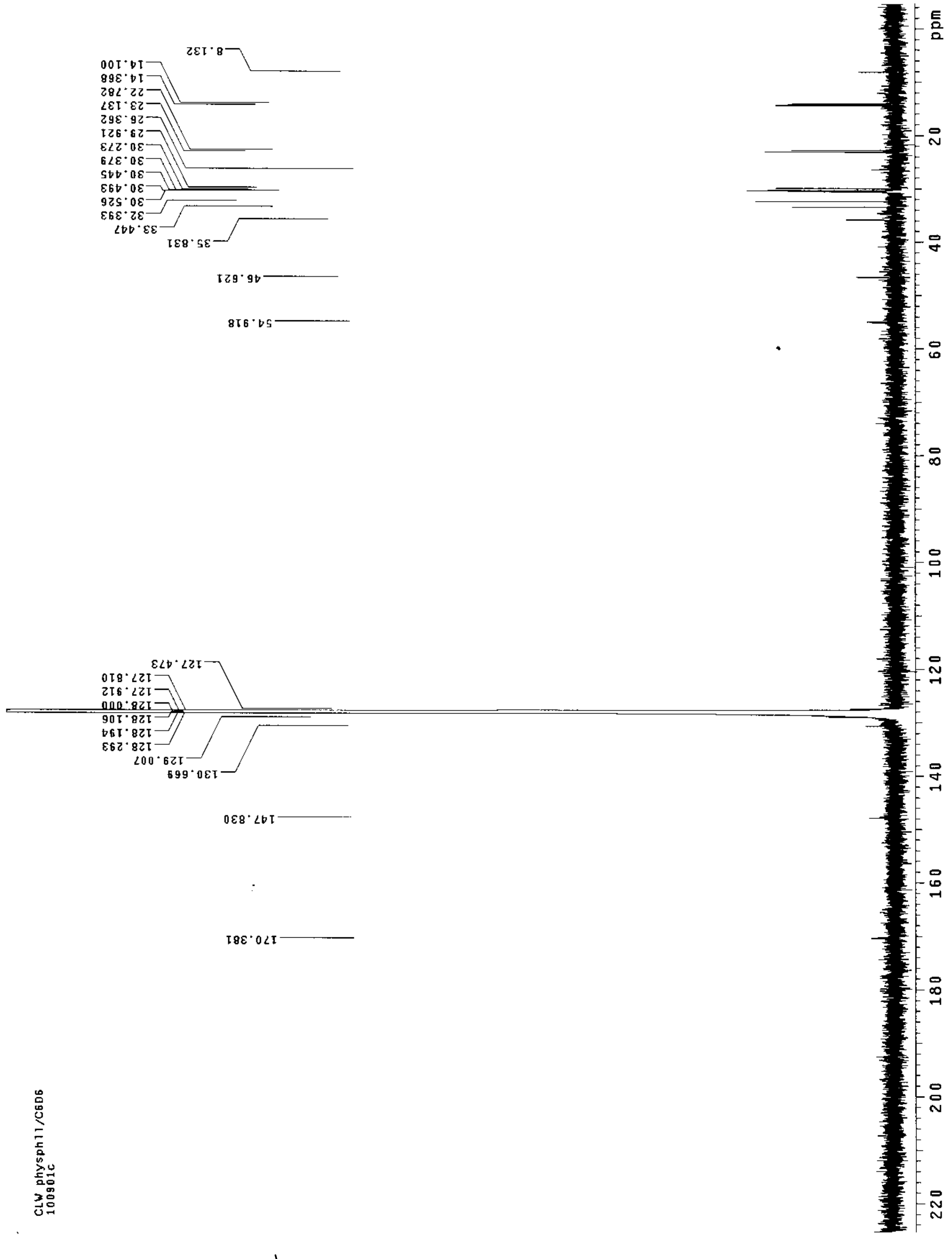
CLW physphll/C6D6
100901c
ppm
8.132
14.100
14.366
22.782
23.137
26.362
29.921
30.273
30.379
30.445
30.493
30.526
32.393
33.447
35.831
46.621
54.918
127.473
127.810
127.912
128.000
128.106
128.194
128.293
129.007
130.669
147.830
170.381
20
40
60
80
100
120
140
160
180
200
220

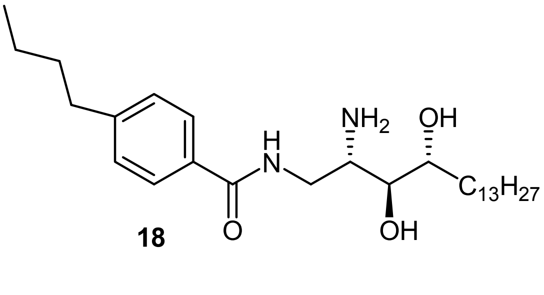
H
N
NH2
OH
OH
O
C13H27

18

CLWphysph11

2010090313 60 (3.110) Cn (Cen,3, 80.00, Ht); Sm (SG, 2x0.75); Sb (3,40.00); Cm (60:62-7:19) Scan ES+ 3.76e7

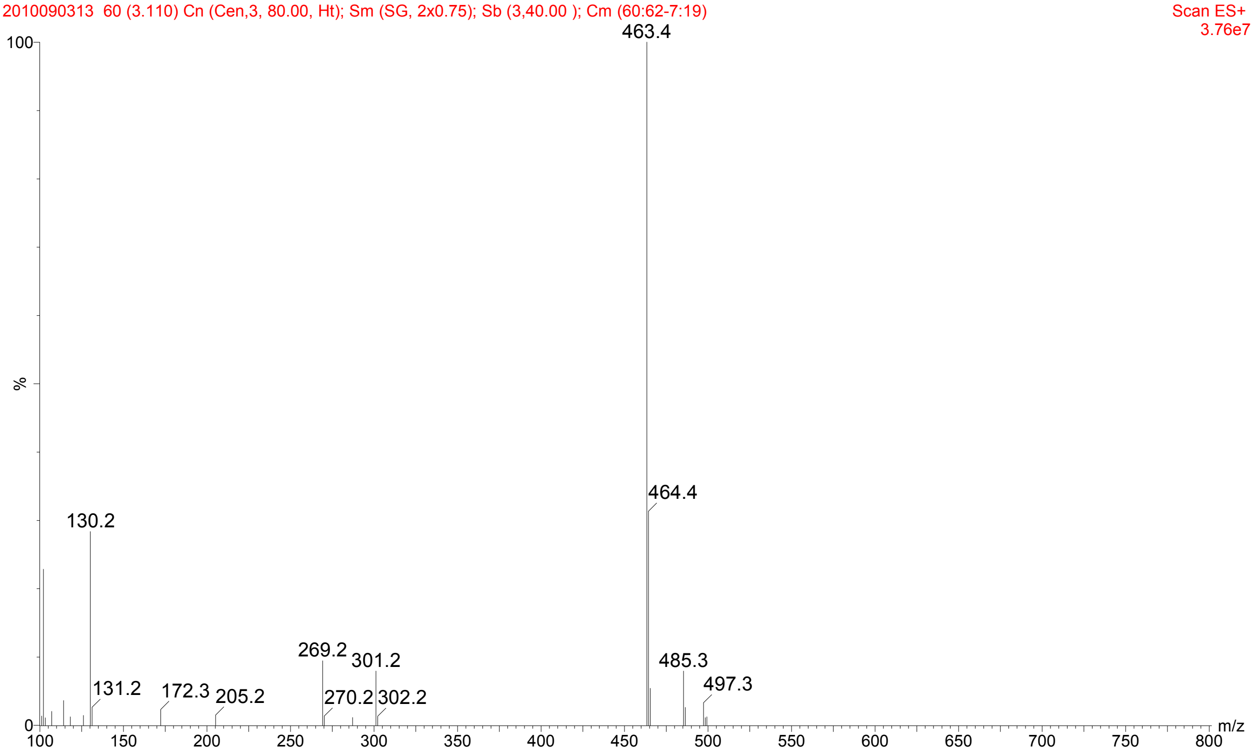

101.0	4.995e5
102.1	8.586e6
103.2	4.086e5
107.0	7.572e5
114.1	1.361e6
118.1	4.668e5
126.0	5.419e5
130.2	1.065e7
131.2	9.776e5
172.3	8.613e5
205.2	5.447e5
269.2	3.542e6
270.2	4.965e5
287.2	4.321e5
301.2	2.977e6
302.2	4.835e5
463.4	3.758e7
464.4	1.177e7
465.4	2.025e6
485.3	2.978e6
486.4	9.812e5
497.3	1.221e6
498.3	4.184e5
499.3	4.683e5

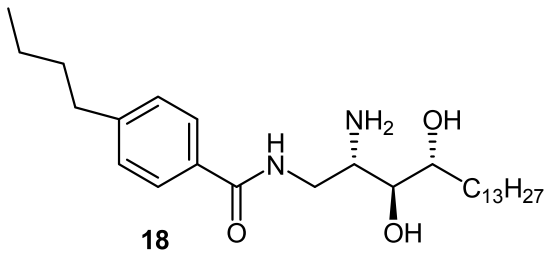

Figure 2. Chromatogram for the mixtures of amide compound **18** after purification with column chromatography.

Figure 3. HPLC chromatogram derived from the reinjection of the collected fraction shown in Fig. 1.

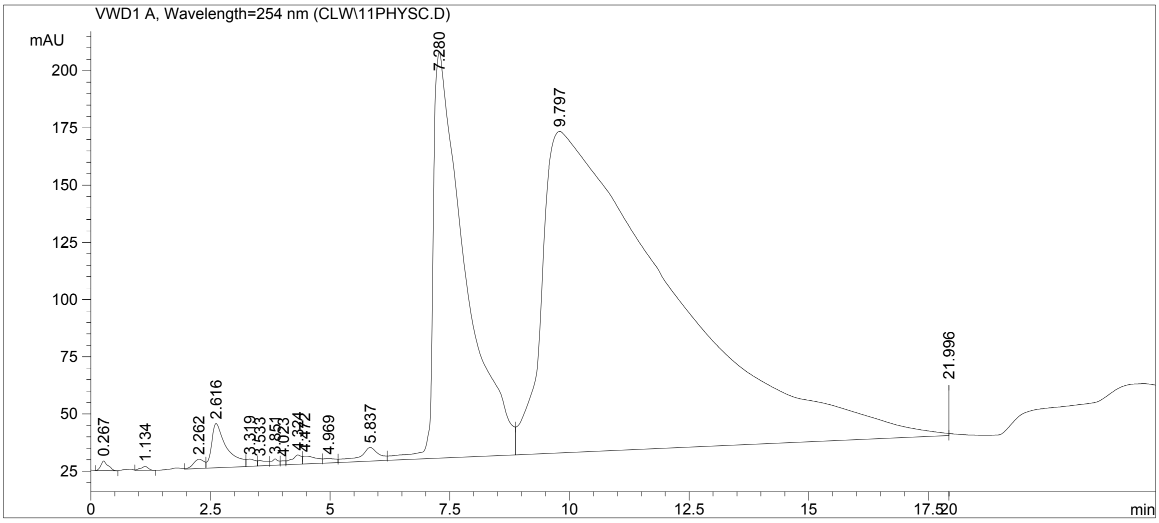

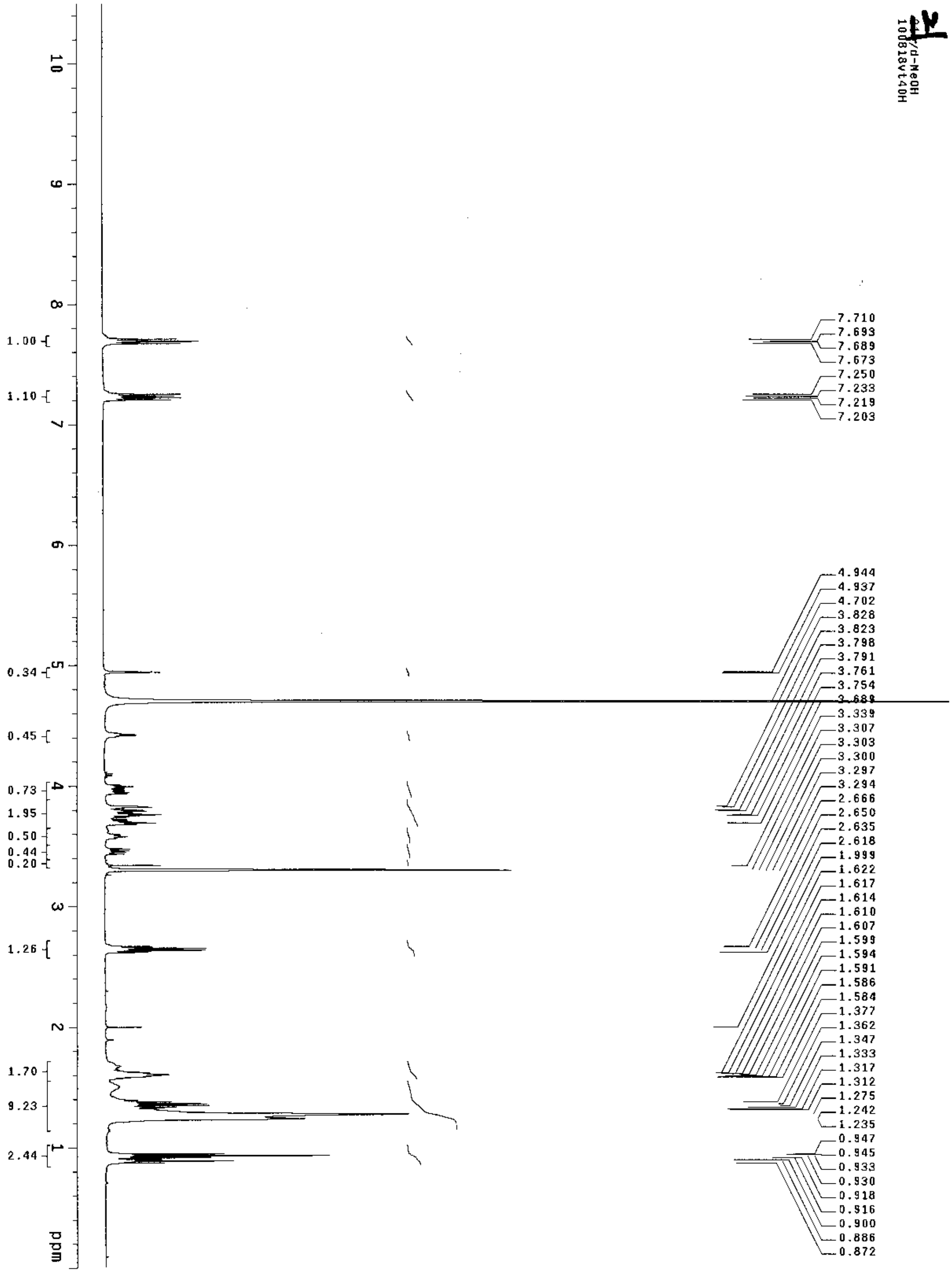
d-MeOH
7.710
7.693
7.689
7.673
7.250
7.233
7.219
7.203
4.944
4.937
4.702
3.828
3.823
3.798
3.791
3.761
3.754
3.689
3.339
3.307
3.303
3.300
3.297
3.294
2.666
2.650
2.635
2.618
1.999
1.622
1.617
1.614
1.610
1.607
1.599
1.594
1.591
1.586
1.584
1.377
1.362
1.347
1.333
1.317
1.312
1.275
1.242
1.235
0.947
0.945
0.933
0.930
0.918
0.916
0.900
0.886
0.872
1.00
1.10
0.34
0.45
0.73
1.95
0.50
0.44
0.20
1.26
1.70
9.23
2.44
10
9
8
7
6
5
4
3
2
1
ppm

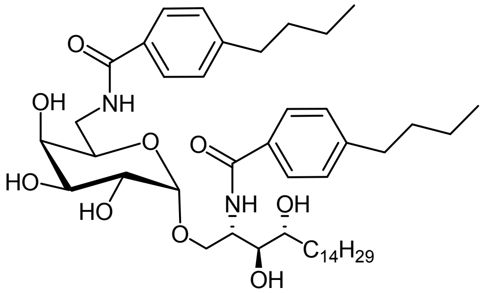
O
OH
NH
O
HO
HO
O
NH
OH
O
C14H29
OH

19

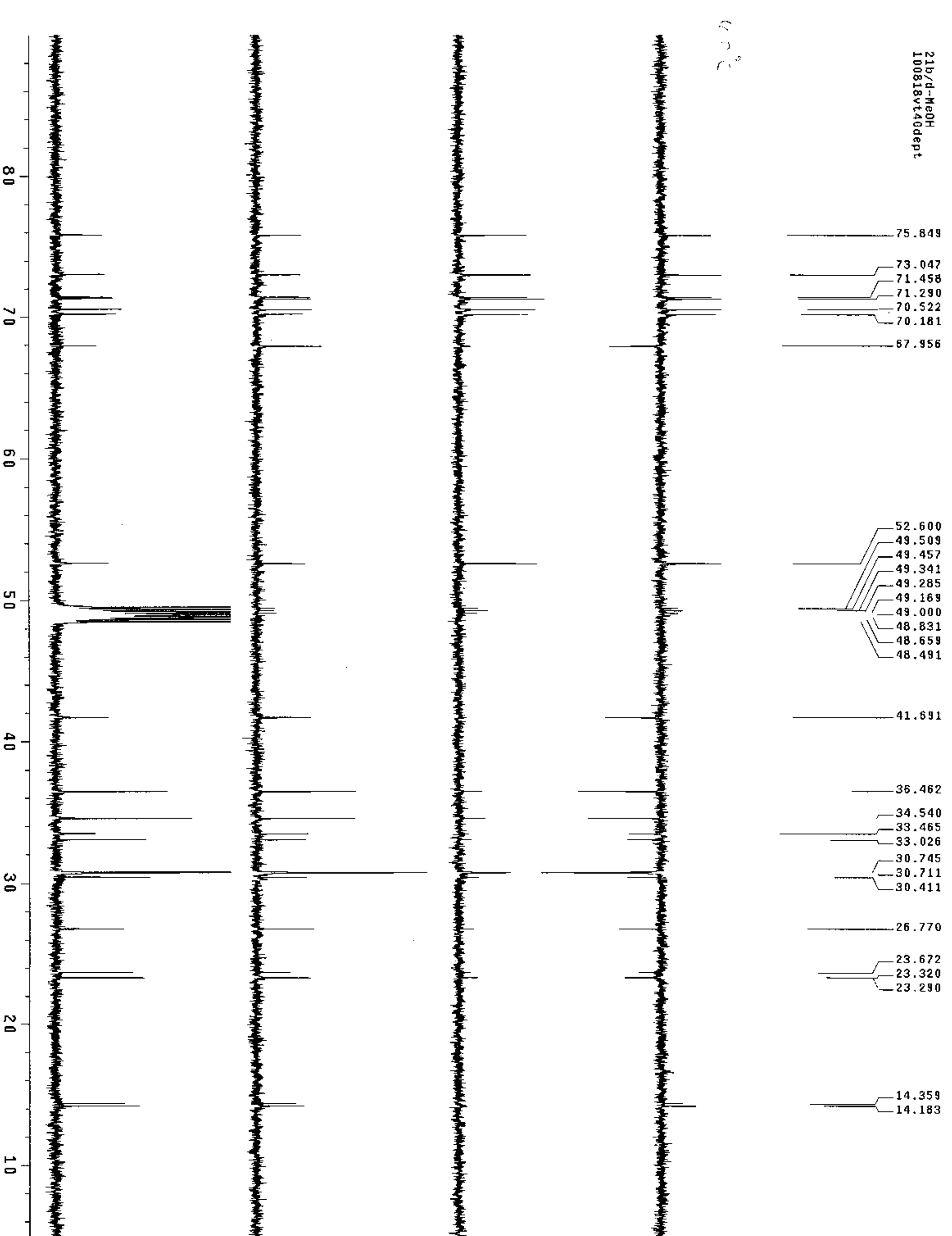
21b/d-MeOH
100818vt40dept
75.849
73.047
71.458
71.290
70.522
70.181
67.956
52.600
49.509
49.457
49.341
49.285
49.169
49.000
48.831
48.659
48.491
41.691
36.462
34.540
33.465
33.026
30.745
30.711
30.411
26.770
23.672
23.320
23.290
14.359
14.183
80
70
60
50
40
30
20
10
ppm

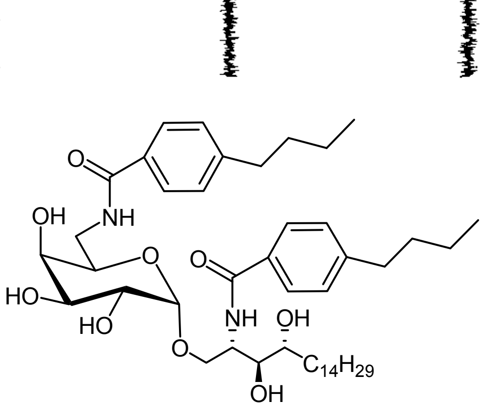
O
NH
OH
O
HO
HO
O
NH
OH
OH
$C_{14}H_{29}$

19

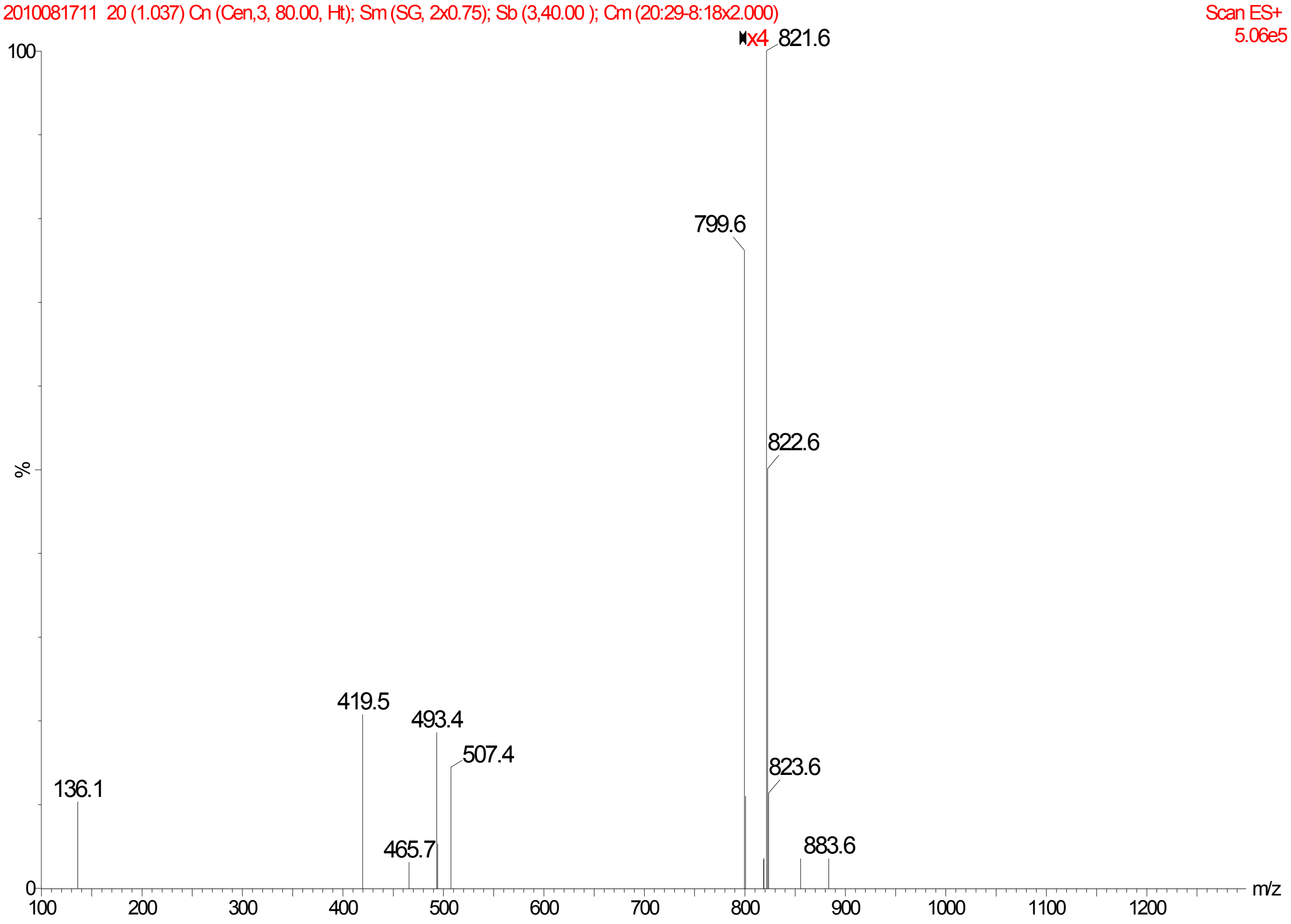

2010081711 20 (1.037) Cn (C

No	Mass	Inten	%BPI	%TIC
1:	136.1	5.21e4	10.30	2.04
2:	419.5	1.05e5	20.73	4.10
3:	465.7	1.56e4	3.09	0.61
4:	493.4	9.40e4	18.59	3.68
5:	494.3	2.68e4	5.30	1.05
6:	507.4	7.31e4	14.44	2.86
7:	508.4	1.22e4	2.41	0.48
8:	799.6	9.63e4	19.04	3.77
9:	800.6	5.56e4	10.99	2.18
10:	818.6	1.72e4	3.40	0.67
11:	819.0	1.80e4	3.56	0.71
12:	819.7	1.31e4	2.60	0.51
13:	821.6	5.06e5	100.00	19.79
14:	822.6	2.53e5	50.09	9.91
15:	823.6	5.73e4	11.33	2.24
16:	835.5	1.02e4	2.03	0.40
17:	855.5	1.78e4	3.51	0.70
18:	883.6	1.78e4	3.51	0.70
19:	889.6	1.18e4	2.34	0.46

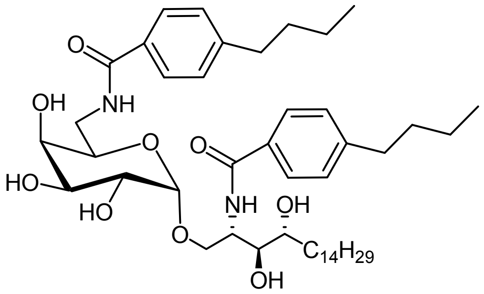

19

Figure 4. HPLC Chromatogram for the mixtures of amide compound **19** obtained from purification through column chromatography.

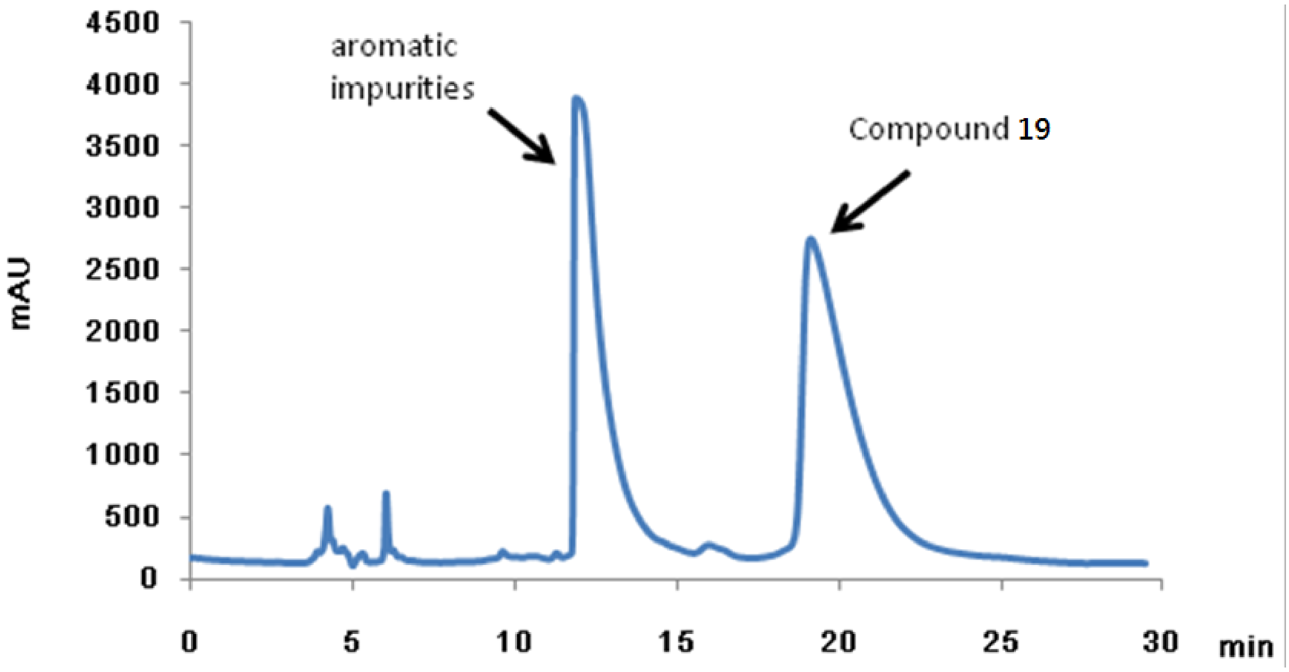

Supplement: Supplementary file 1 [file molecules-17-03058-s001.pdf]
